# Large-Scale In Silico Mapping of Complex Quantitative Traits in Inbred Mice

**DOI:** 10.1371/journal.pone.0000651

**Published:** 2007-07-25

**Authors:** Pengyuan Liu, Haris Vikis, Yan Lu, Daolong Wang, Ming You

**Affiliations:** Department of Surgery and the Alvin J. Siteman Cancer Center, Washington University School of Medicine, St. Louis, Missouri, United States of America; DER Neurogenetics, National Institute of Neurological Disorders and Stroke, United States of America

## Abstract

Understanding the genetic basis of common disease and disease-related quantitative traits will aid in the development of diagnostics and therapeutics. The processs of gene discovery can be sped up by rapid and effective integration of well-defined mouse genome and phenome data resources. We describe here an *in silico* gene-discovery strategy through genome-wide association (GWA) scans in inbred mice with a wide range of genetic variation. We identified 937 quantitative trait loci (QTLs) from a survey of 173 mouse phenotypes, which include models of human disease (atherosclerosis, cardiovascular disease, cancer and obesity) as well as behavioral, hematological, immunological, metabolic, and neurological traits. 67% of QTLs were refined into genomic regions <0.5 Mb with ∼40-fold increase in mapping precision as compared with classical linkage analysis. This makes for more efficient identification of the genes that underlie disease. We have identified two QTL genes, *Adam12* and *Cdh2*, as causal genetic variants for atherogenic diet-induced obesity. Our findings demonstrate that GWA analysis in mice has the potential to resolve multiple tightly linked QTLs and achieve single-gene resolution. These high-resolution QTL data can serve as a primary resource for positional cloning and gene identification in the research community.

## Introduction

Humans and mice last shared a common ancestor 75 million years ago. Of mouse genes, 99% have homologues in humans and 96% are in the same syntenic location. Regions of the genome that are highly conserved between the two species align well with functionality [1]. The mouse has been a powerful force in elucidating the genetic basis of human physiology and pathophysiology [2]. Many genetic alterations identified in mouse models have a common genetic equivalent (or ortholog) in humans. In the past decades, quantitative trait locus (QTL) mapping has identified hundreds of chromosomal regions containing genes affecting cancer, diabetes, hypertension, obesity and other disease-related phenotypes in mice. Most of these complex traits show clear genetic components, yet the underlying genes remain largely elusive.

QTL analysis using mouse models can potentially identify genes that are important for human diseases, which are often difficult to identify in human populations, due to the complexity of human genetic data. However, a major obstacle of identifying QTL genes is the difficulty of resolving these chromosomal regions (10∼20 cM) into sufficiently small intervals to make positional cloning possible. Recent advances in genomic sequence analysis and SNP discovery have provided researchers with the necessary resources to explore a wide range of genetic variation in laboratory inbred mice [3]. The use of dense SNP maps in laboratory inbred mice has proven successful in the refinement of previous QTL regions and the identification of new genetic determinants of complex traits [4]–[8]. Recently, positional cloning of a novel gene underlying a complex trait was done immediately after a genome-wide association (GWA) analysis [4].

**Figure 1:**
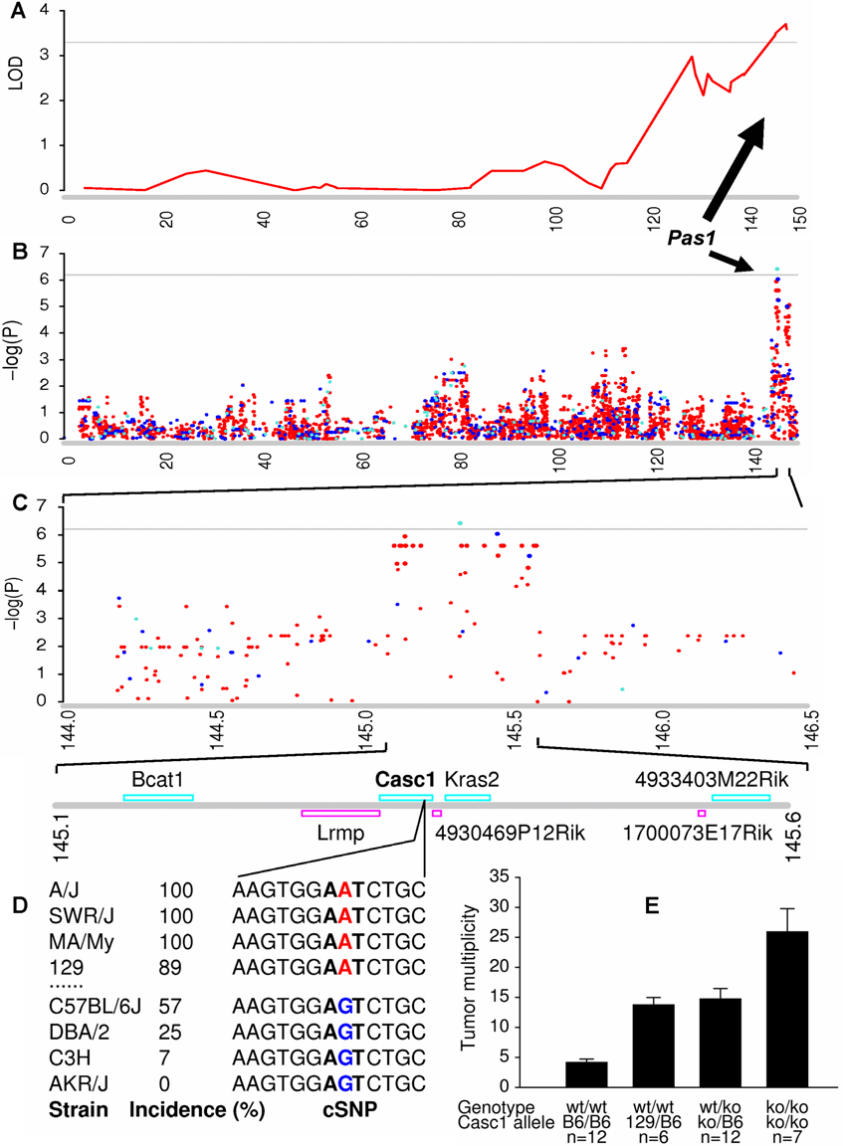
Integration of genomics and phenomics approaches in mapping complex traits in mice. (A) Pulmonary adenoma susceptibility locus 1 (*Pas1*) was previously mapped to the distal region of chromosome 6 by linkage analysis in A×B and B×A recombinant inbred mice. (B) *In silico* LD mapping *Pas1* using existing mouse SNP and tumor incidence data. (C) Enhanced view of LD mapping between 144.0–146.5 Mb. (D) Physical map of the complex multigenic *Pas1* locus. The *Casc1* gene carries a functional polymorphism at codon 60 (N60S) between lung tumor susceptible strains (e.g. A/J, SWR/J, MA/MyJ and 129/J) and resistant strains (e.g. C57BL/6J, DBA/2J, C3H/HeJ and AKR/J). (E) Lung tumorigenesis of *Casc1* knockout mice. Treatment of Casc1^ko/ko^ mice with urethane produced more than six times as many tumors per mouse than did treatment of Casc1^wt-B6/wt-B6^ mice. The horizontal coordinates were plotted using physical distance (Mb). In panel A, the horizontal line indicates lod = 3.3, and tumor multiplicity data were used in the linkage analysis. In panel B and C, the horizontal lines indicate –log(P) = 6.2, and urethane-induced lung adenoma incidence data were used in the analysis [4].

A large and diverse set of traits has been systematically organized in the publicly accessible Mouse Phenome Database (MPD) (www.jax.org/phenome), housed at The Jackson Laboratory (Bar Harbor, Maine, United States). Rapid and effective integration of mouse genome and phenome data resources can dramatically speed up the process of gene discovery (**Figure 1**). Here, we describe an *in silico* gene-discovery strategy through GWA scans to identify high-resolution QTLs in inbred mice. We further demonstrate that GWA analysis in mice has the potential to resolve multiple tightly linked QTLs and achieve single-gene resolution.

## Results

### The mouse haplotype map

We first evaluated the laboratory inbred mouse haplotype map that was used in the analysis. This haplotype map was generated by a joint effort of The Broad Institute of Harvard and MIT, and the Wellcome Trust Center for Human Genetics (**Text S1**). In total, 148,062 evenly distributed single-nucleotide polymorphisms (SNPs), which span the mouse genome at an average density of ∼18 kb per SNP, were genotyped in classical inbred mouse strains (**Figure S1**). Of these SNPs, 96.2% have minor allele frequencies greater than 0.05 and 76.7% of the inter-SNP distances are smaller than 20 kb. LD decay over the physical distance was observed where the mean *r^2^* fell to <0.5 within 2 kb and to less than 0.23 within 200 kb. To capture large-scale patterns of LD, we also measured the number of proxies, i.e. SNPs, showing a strong correlation with one or more others [9], with a window size of 500 kb across the genome (**Figure S2**). Using a stringent threshold of *r^2^* = 1.0 (that is complete LD), the average number of proxies per SNP is 2.5, and one in two SNPs have five or more proxies. Of all the SNPs, 89.5% have an *r^2^* = 1.0 with at least one other SNP. Using a looser threshold of *r^2^*>0.5, the average number of proxies is increased to 6.5, and two in three SNPs have more than five proxies. Of all the SNPs, 91.3% have an *r^2^*>0.5 with at least one other SNP. These data indicate that the mouse haplotype map provides substantial coverage of genetic variation in inbred mice and is useful for high-resolution genetic mapping studies.

### Analysis of 173 mouse phenotypes

We performed a comprehensive survey of 173 complex quantitative traits from the mouse PHENOME projects instituted by the Jackson Laboratory (www.jax.org/phenome). In most PHENOME projects, each phenotype was measured for about 40 strains from a total of 59 inbred strains of mice with a wide range of genetic variation (**Figure S3** and **Text S1**). The strains used for phenotyping varied slightly among different PHENOME projects. Generally, at least 10 males and 10 females from each strain were measured for each trait. These complex traits include models of human disease (such as atherosclerosis, cardiovascular disease, cancer, diabetes, obesity, and osteoporosis) as well as behavioral, biochemical, hematological, immunological, metabolic, neurological, and reproductive traits (**Table 1**). Most of these 173 mouse quantitative traits are highly heritable, with a mean heritability of 0.51, and 74% of these traits have a heritability larger than 0.40 (**Figure S4**). In general, cancer, taste and drinking preferences show less genetic control (*h^2^*<0.3), while body composition and plasma lipid concentrations show larger genetic control (*h^2^*>0.7). Of these mouse traits, 47% display significant phenotypic differences between males and females (P<0.05), contributing to 1–31% of the phenotypic variation in the inbred mouse populations. Notably, obesity-related traits such as body weight and fat mass have larger sex effects and explain more than 20% of the phenotypic variation in inbred mice. Nevertheless, sex-specific genetic variations are generally small (less than 6%) in the inbred mouse populations.

**Table 1:** Summary of the analyzed mouse traits and QTLs

Categories	Description	Traits	QTLs
Blood biochemistry	Blood calcium and pH, coagulation factors, glucose, leptin, insulin, peptides and proteins	13	59
Body composition	Body weight, fat mass and lean mass on normal chow and atherogenic diet	24	118
Cancer	Metastatic progression, tumor latency, and tumor burden	8	2
Cholesterol	Total cholesterol, HDL cholesterol, nonHDL cholesterol, and hepatic cholesterol	20	206
Drinking preference	Drinking preference for ammonium, calcium, potassium, sodium chloride and/or lactate	29	137
Gallbladder and gallstones	Gallbladder volume, mucin, liquid crystals, monohydrate crystals, sandy stones, and gallstones	10	23
Hematology	Complete blood count, red blood cell parameters, and platelets	19	128
Liver pathology	Diet-induced liver pathology including liver weight, alanine aminotransferase, bile salts, microvacuoles, macrovacuoles and inflammation	6	22
Musculoskeletal	Bone mineral content and density	4	59
Neurological	Acoustic startle response, hearing, and prepulse inhibition	18	123
Reproduction	Reproductive performance such as number of litters per dam and number of mice per litter	11	4
Triglycerides	Triglycerides on normal chew and atherogenic diet	4	56
Others	Daily average food and water intake	7	0

### Identification of 937 QTLs

**Figure 2:**
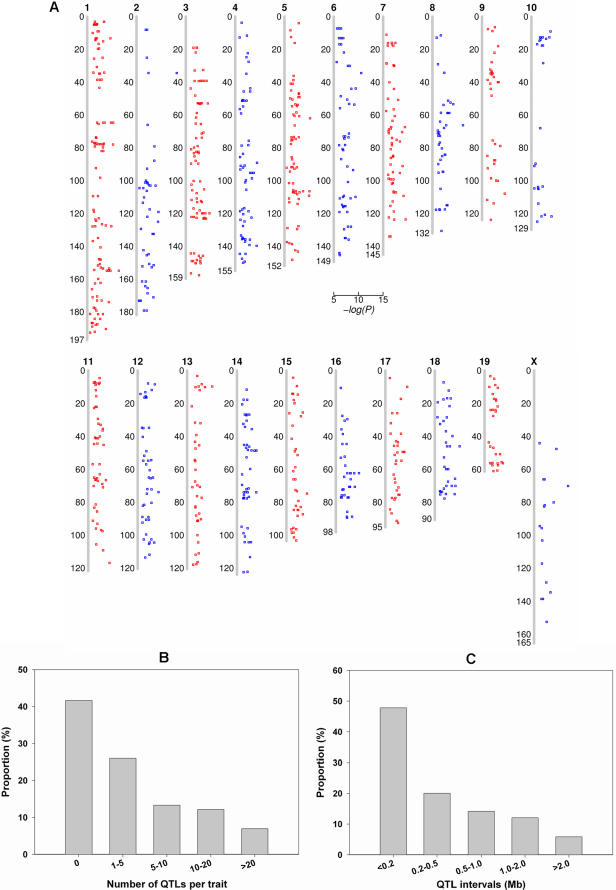
937 QTLs identified by genome-wide association analysis. (A) Distribution of 937 QTLs on the mouse genome. The scatter plots were drawn for –log(P) against the peak of QTLs on the chromosomes. Each square represents an identified QTL. (B) The number of QTLs per trait. (C) Proportion of QTL intervals in Mb.

To identify the genetic basis of this large and diverse set of mouse phenotypes, we designed an *in silico* approach for gene identification through GWA scans in inbred mice. In our approach, GWA scans were implemented in an automatic processing pipeline which constitutes data retrieving, outlier detection, data preprocessing, hypothesis testing, permutation testing and QTL identification. The potential problem of population structure in inbred mice was also inspected (**Figure S5** and **Text S1**). Totally, we identified 937 QTLs from the survey of 173 complex quantitative traits in inbred mice. These QTLs affect almost all types of mouse phenotypes and are distributed unevenly on all of the mouse chromosomes except chromosome Y (**Figure 2A**, and **Table S1** and **S2**). The number of QTLs per trait ranges from 0 to 38, with a mean of 5.4 (**Figure 2B**). The average size of a QTL region is 0.54 Mb and 67% of the QTLs were mapped to genomic regions less than 0.5 Mb (**Figure 2C**). This translates to an extraordinary ∼40-fold increase in mapping precision as compared with clasical linkage analysis. In addition, about 20% of the QTLs were located within genomic regions less than 100 kb away from at least one other QTL, implying the prevalence of pleiotropic effects of genetic manipulation in mice.

**Figure S6** shows a general QTL landscape from our GWA scans of several obesity-related phenotypes, including body weight and lean mass on normal chow and high fat, high cholesterol atherogenic diets. The analyses identified a large number of QTLs, most of which contained multiple significant SNPs clustering in the same genomic region. Several QTLs affecting multiple obesity phenotypes were repeatedly mapped to the identical locations on distal chromosome 1 and proximal chromosome 13 in different datasets. Most of these QTLs are broadly concordant with those detected previously by linkage analyses which, however, normally encompassed 10–20 cM (about 20 to 40 Mb on the mouse genome) [10]. In some scenarios, several separated QTLs from the GWA scans were mapped to a single linkage-defined QTL region. This lack of precision is mainly due to limited recombinants in these linkage mapping populations. Our GWA scans also found several novel QTLs that do not overlap with previous linkage analysis regions (**Figure S6**). For example, we fine mapped a novel obesity QTL to a region of approximately 700 Kb (33.98–34.71 Mb) on proximal chromosome 9, which covers two candidates: Kirrel3 and A130066N16Rik; and mapped a novel QTL to a region of approximately 100 Kb (56.28–56.37 Mb) on distal chromosome 19, which covers Habp2 and Nrap. These novel QTLs identified in the GWA are not unexpected since association mapping detects allelic associations at the level of the population, which is normally comprised of 30–40 genetically diverse inbred mouse strains, rather than allelic difference between two specific parental strains in linkage mapping. Furthermore, all the obesity QTL regions detected by the GWA analyses using natural inbred mouse population samples are much smaller (less than 0.6 Mb on average with the present SNP density of 18 kb per SNP) as compared with linkage analyses. This makes positional cloning feasible immediately after GWA scans without the need for further time-comsuming fine mapping with congenic strains.

### Dissection of multiple tightly linked QTLs

**Figure 3:**
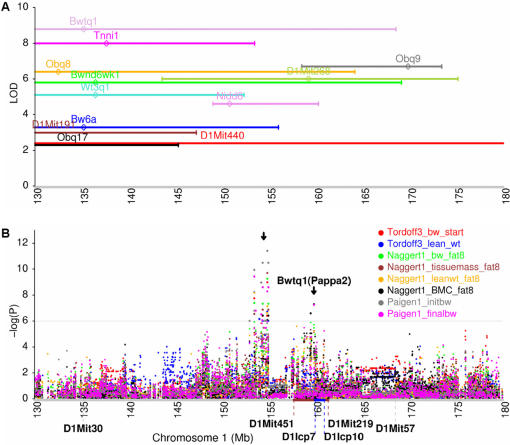
Dissection of multiple tightly linked QTLs. (A) Obesity QTLs were mapped to distal mouse hromosome 1 by linkage analysis from cross-breeding experiments [10]. Approximately one LOD interval was plotted against its LOD at the QTL peak. The diamonds indicate QTL peaks. Each peak lists the names of the defined QTL. (B) LD mapping of several obesity-related phenotypes in distal mouse chromosome 1. LD mapping identified two closely linked QTLs on distal chromosome 1. One is located within 153.5–155.5 Mb; another reproduced the previously refined QTL, Bwtq1. The analyzed phenotypes are body weight at the start of testing (8 weeks) (Tordoff3_bw_start), calculated weight of lean tissue (14 weeks) (Tordoff3_lean_wt), body weight after 8 weeks on an atherogenic diet (Naggert1_bw_fat8), total tissue mass after 8 weeks on an atherogenic diet (Naggert1_tissuemass_fat8), weight of lean portion of tissue mass after 8 weeks on an atherogenic diet (Naggert1_leanwt_fat8), bone mineral content after 8 weeks on an atherogenic diet (Naggert1_BMC_fat8), initial body weight (7–9 weeks) (Paigen1_initbw), and final body weight after 8 weeks on an atherogenic diet (Paigen1_finalbw).

GWA scans help resolve multiple, tightly linked QTLs. We again used obesity QTLs to demonstrate this potential. So far, at least 12 obesity QTLs have been mapped to distal mouse chromosome 1, and most of them overlap with each other and may be linked to the same gene (**Figure 3A**). Among these, Bwtq1 was originally identified in a cross between DBA/2J and C57BL/6J [11] and later refined to an 8-cM region (∼33 Mb) between D1Mit30 and D1Mit57 [12]. This QTL has a general effect on body size, affecting the length of the tail and most bones studied, as well as having a weaker effect on mass. Using congenic strains, Bwtq1 was narrowed down to a 1.4-cM region (∼4 Mb), approximately the region from D1Mit451 to D1Mit219 [13]. The location of Bwtq1 was further refined to 0.91 Mb between D1Icp7 and D1Icp10 using interval-specific subcongenic strains, which led to the identification of a strong candidate, *Pappa2*, encoding an enzyme that cleaves an insulin-like growth factor binding protein (**Figure 3B**) [14]. However, our GWA scans identified two closely linked QTLs within 5 Mb of each other on distal chromosome 1. One is associated with several different obesity phenotypes in three independent datasets and is located within 153.5–155.5 Mb (–log(P) = 8.2–11.4) and the other reproduced the refined Bwtq1 QTL, showing significant effects on body weight and bone mineral content (–log(P) = 7.2). These results illustrate the robustness and reproducibility of GWA scans for complex quantitative traits in inbred mice.

### Murine knockout and/or transgenic models

**Figure 4:**
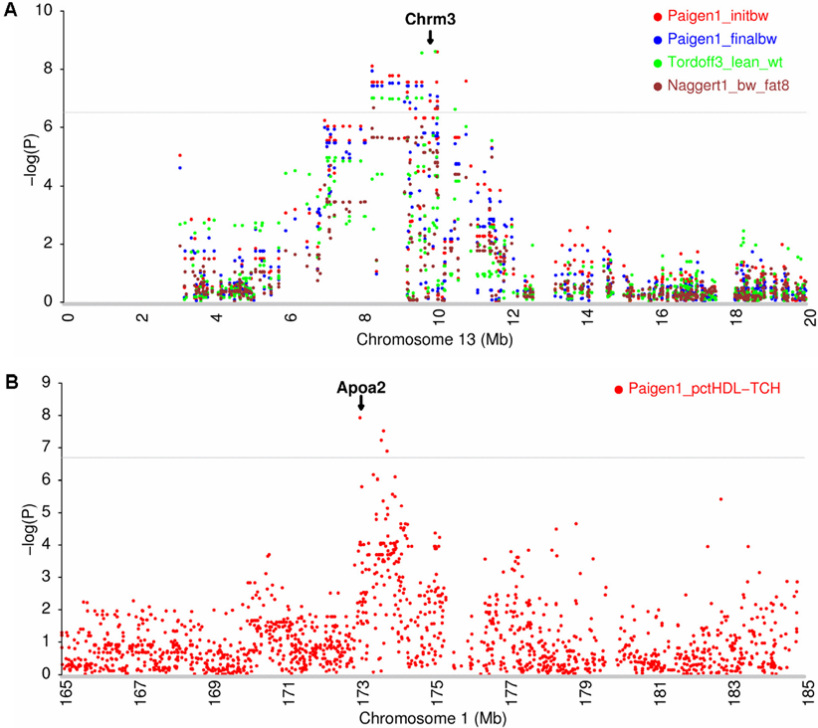
Fine mapping of Chrm3 and Apoa2. (A) Chrm3 is associated with initial body weight (7–9 weeks), day 0 of an atherogenic diet (Paigen1_initbw), final body weight after 8 weeks on atherogenic diet (Paigen1_finalbw), calculated weight of lean tissue (14 weeks) (Tordoff3_lean_wt), and body weight after 8 weeks on an atherogenic diet (Naggert1_bw_fat8) in the GWA scan. The Chrm3 region has been replicated in at least four obesity linkage studies. (B) Apoa2 is associated with percent of total plasma cholesterol in the HDL fraction after 8 weeks on an atherogenic diet (Paigen1_ pctHDL-TCH) in the GWA scan. The Apoa2 region has been repeatedly detected by linkage analyses in 12 different genetic crosses. Relevant phenotypes for these two genes were already observed in murine knockout models. The horizontal gray lines indicate genome-wide empirical thresholds.

The refined region of previous QTLs was reduced, on average, to 0.54 Mb, which covers on average 6 genes on the mouse genome. Characterization of such a small number of genes in each of these QTLs can greatly accelerate the discovery of genes underlying complex traits. We further performed a detailed survey of QTLs for all obesity-related phenotypes such as body weight, fat mass, lean mass and leptin levels. Of the QTLs detected in the present study, we identified 10 candidate genes within these QTLs that, when mutated or overexpressed as transgenes in the mouse, result in phenotypes affecting body weight and adiposity. These genes include Gpr39 (G protein-coupled receptor 39), Crhr2 (corticotropin releasing hormone receptor 2), Cdkn1b (cyclin-dependent kinase inhibitor 1B), Adam12 (ADAM metallopeptidase domain 12), Rasgrf1 (RAS protein-specific guanine nucleotide-releasing factor 1), Chrm3 (cholinergic receptor, muscarinic 3), Mapk8 (mitogen activated protein kinase 8), Wnt10b (wingless related MMTV integration site 10b), Dusp1 (dual specificity phosphatase 1) and Cdh2 (cadherin 2) (**Table 2**). For example, an obesity QTL on the proximal chromosome 13 has been replicated in at least four linkage studies [15]–[18], and has not yet been narrowed down. Our GWA scans identified a refined QTL (8.0–10.0 Mb) covering the gene Chrm3 to be associated with four obesity phenotypes in three independent datasets (-log(P) = 6.7–8.6) (**Figure 4A**). Chrm3 is a member of the muscarinic acetylcholine receptors that play critical roles in regulating the activity of many important functions of the central and peripheral nervous system [19]. Chrm3 knockout mice had a significant reduction in food intake, body weight (25%), and peripheral fat deposits. The lack of Chrm3 also led to pronounced reductions (∼5–10-fold) in serum leptin and insulin levels [20].

**Table 2:** Candidate genes for obesity validated in murine knockout and/or transgenic models

Gene^a^	Chr	QTL peak^b^	Analyzed trait^c^	*-log(P)*	Phenotypes in KO or TG mice^d^	Ref.
Gpr39	1	127,875,052	Percent of tissue mass that is fat, after 8 wks on atherogenic diet (Naggert1)	*6.1*	Increased body weight and body fat composition (KO)	[27]
Crhr2	6	55,858,855	Leptin, after 18 wks on atherogenic diet (Naggert1)	*6.3*	Lower body fat and plasma lipids, increased insulin sensitivity, and lower feed efficiency on high-fat diet (KO)	[47]
Cdkn1b	6	134,947,686	Percent fat (Tordoff3)	7.4	Increased body fat percentage, annd increased adiposity (KO)	[48]
		134,947,686	Percent lean (Tordoff3)	7.3		
Adam12	7	133,991,842	Percent of tissue mass that is fat, after 8 wks on atherogenic diet (Naggert1)	*6.5*	Moderate resistance to diet-induced obesity due to an impairment in the increase of the number of adipocytes in high-fat-fed mice (TG)	[24]
Rasgrf1	9	89,769,476	Percent fat (Tordoff3)	*7.5*	Reduced body weight, adiposity and ∼20% decreased gonadal fat percentage (KO)	[49]
Chrm3	13	8,305,114	Final body weight after 8 wks on atherogenic diet (Paigen1)	7.9	Reduced adiposity and decreased ∼25% body weight (KO)	[20]
		10,048,424	Calculated weight of lean tissue (Tordoff3)	8.6		
		10,065,064	Initial body weight (Paigen1)	8.6		
		10,065,799	Body weight after 8 wks on atherogenic diet (Naggert1)	6.7		
Mapk8	14	32,376,554	Leptin, after 18 wks on atherogenic diet (Naggert1)	*6.3*	Reduced body weight and increased insulin sensitivity (KO)	[50]
Wnt10b	15	98,434,034	Percent of tissue mass that is fat, after 8 wks on atherogenic diet (Naggert1)	*6.1*	Tesistant to diet-induced obesity and loss of brown adipose tissue (TG)	[51]
Dusp1	17	25,910,596	Percent of tissue mass that is fat, after 8 wks on atherogenic diet (Naggert1)	*7.4*	Resistant to diet-induced obesity but succumb to glucose intolerance on a high fat diet (KO)	[52]
Cdh2	18	16,917,241	Liver weight at sacrifice, after 8 wks on atherogenic diet (Paigen1)	*8.2*	Increased adiposity (TG)	[25]

a Genes were located within the identified QTLs.

b Physical position (bp) was based on the NCBI mouse genome build 36.1.

c Traits were analyzed in the GWA scans; name of the PHENOME projects was indicated in the parentheses.

d Relevant phenotypes were observed in murine knockout (KO) and/or transgenic (TG) models.

Similarly, we performed a survey of QTLs for plasma lipid levels which are correlated with the risk of atherosclerotic coronary heart disease [21]. We identified 11 candidate genes in the refined regions of QTLs affecting levels of high density lipoprotein (HDL) cholesterol, low density lipoprotein (LDL) cholesterol, and triglycerides. These genes are Gpr39 (G protein-coupled receptor 39), Apoa2 (apolipoprotein A-II), Cd36 (CD36 antigen), Cckar (cholecystokinin A receptor), Npy (neuropeptide Y), Pparg (peroxisome proliferator activated receptor gamma), Ccr2 (chemokine (C-C motif) receptor 2), Acaca (acetyl-Coenzyme A carboxylase alpha), Apob (apolipoprotein B), Soat2 (sterol O-acyltransferase 2) and Lipg (endothelial lipase) (**Table 3**). All of these genes were observed to have strong involvement in plasma lipid phenotypes in murine knockout and/or transgenic models. Notably, we identified a QTL containing Apoa2 on chromosome 1 located at 173.0–173.9 Mb, which affects the percent of total plasma cholesterol in the HDL fraction after 8 weeks on an atherogenic diet (-log(P) = 7.9) (**Figure 4B**). Apoa2 is located within a strong HDL QTL, hdlq5, which has been repeatedly detected on distal chromosome 1 by linkage analyses in 12 different genetic crosses [22]. Homozygous Apoa2 knockout mice had 67% and 52% reductions in HDL cholesterol levels in the fasted and fed states, respectively, and HDL particle size was also reduced [23].

**Table 3:** Candidate genes for plasma lipid levels validated in murine knockout and/or transgenic models

Gene^a^	Chr	QTL peak	Analyzed trait	*-log(P)*	Phenotypes in KO or TG mice	Ref.
Gpr39	1	127,218,849	Total cholesterol, after 17 wks on atherogenic diet (Paigen2)	8.8	Increased 11% and 42% cholesterol levels in males and females, respectively (KO)	[27]
		127,775,854	Fold change in total cholesterol after 17 wks on atherogenic diet (Paigen2)	6.6		
		127,775,854	Non-HDL cholesterol, after 17 wks on atherogenic diet (Paigen2)	9.9		
Apoa2	1	173,030,894	Percent of total plasma cholesterol in HDL fraction, after 8 wks on atherogenic diet (Paigen1)	*7.9*	Reduced HDL cholesterol levels and HDL particle size (KO)	[23]
Cd36	5	16,119,658	Non-HDL cholesterol, after 17 wks on atherogenic diet (Paigen2)	*7.1*	Increased plasma free fatty acid and triglyceride levels and decreased glucose levels (KO)	[53]
Cckar	5	53,817,933	Triglycerides (Paigen4)	7.5	Increased cholesterol gallstone formation (KO)	[54]
		54,607,854	Triglycerides (Paigen2)	7.7		
Npy	6	50,629,612	Triglycerides (Paigen4)	*7.7*	Decreased 18% and 27% Triglycerides in fasted and refed mice (KO)	[55]
Pparg	6	116,114,107	Hepatic cholesterol concentration, after 8 wks on atherogenic diet (Paigen1)	*6.9*	Reduced cholesterol efflux and HDL levels (KO)	[56]
Ccr2	9	123,799,254	Non-HDL cholesterol, after 17 wks on atherogenic diet (Paigen2)	*6.8*	Decreased 20% and 17% total cholesterol levels in low- and high-fat diet, respectively (KO)	[57]
Acaca	11	85,561,821	Non-HDL cholesterol, after 17 wks on atherogenic diet (Paigen2)	*6.8*	Decrease in the de novo fatty acid synthesis and triglyceride accumulation in the liver (KO)	[58]
Apob	12	7,813,147	Hepatic cholesterol concentration, after 8 wks on atherogenic diet (Paigen1)	*7.1*	Decreased ∼37–39% total cholesterol, LDL, and HDL levels (KO)	[59]
Soat2	15	101,357,816	Hepatic cholesterol concentration, after 8 wks on atherogenic diet (Paigen1)	*6.5*	Resistance to diet-induced hypercholesterolemia and gallstone formation (KO)	[60]
Lipg	18	74,969,883	Triglycerides (Paigen4)	*7.2*	Fasting plasma HDL cholesterol was increased by 57% in Lipg–/– and 25% in Lipg+/– mice and was decreased by 19% in Lipg transgenic mice (KO/TG)	[61]

### Adam12 and Cdh2 mapped with single-gene resolution by GWA scans

**Figure 5:**
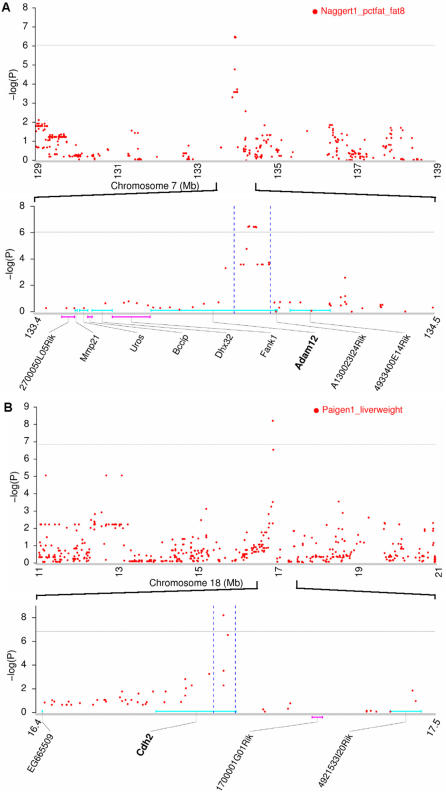
Adam12 and Cdh2 were mapped to single-gene resolution. (A) Adam12 is associated with the percent of fat mass after 8 weeks on an atherogenic diet (Naggert1_pctfat_fat8). (B) Cdh2 is associated with liver weight after 8 weeks on an atherogenic diet (Paigen1_liverweight). Relevant phenotypes for these two genes were already observed in murine knockout or transgenic models. In each subfigure, the upper panel is the enhanced view of LD mapping in a short region around the LD peak, and the lower panel is the physical map of the associated refined QTL. Genes shown on the upper side of the chromosome (turquoise lines) are transcribed in the–orientation (from right to left), and those on the lower side (pink lines) in the+orientation (from left to right). The horizontal gray lines indicate genome-wide empirical thresholds. Physical position was based on the latest NCBI mouse genome build 36.1.

GWA scans have the potential to achieve single-gene resolution, depending on local LD patterns and gene density on the mouse genome. A closer view of the above 10 obesity candidate-gene regions (**Table 2**) further identified two QTLs that were mapped with single-gene resolution. One QTL significantly affects the percent of fat mass after 8 weeks on an atherogenic diet (-log(P) = 6.5) and is located on chromosome 7 spanning less than 100 kb where Adam12 is the only candidate gene (**Figure 5A**). Meltrin alpha (Adam12) is a metalloprotease-disintegrin that belongs to the ADAM (for “a disintegrin and metalloprotease”) family. Compared with wild-type mice, meltrin alpha(-/-) mice displayed moderate resistance to weight gain induced by a high-fat diet, mainly because of an increase in the number of adipocytes [24]. Another QTL is significantly associated with mouse liver weight after 8 weeks on an atherogenic diet (-log(P) = 8.2) and is located on chromosome 18, spanning less than 100 kb, where Cdh2 is the only candidate gene (**Figure 5B**). N-cadherin (Cdh2) is a classical cadherin from the cadherin superfamily. Targeted expression of a dominant-negative N-cadherin in vivo delays peak bone mass and increases adipogenesis when compared with wild-type littermates [25]. Since Adam12 and Cdh2 are the only candadiate gene in each of their QTL regions and further supported by the evidence from knockout and/or transgenic mice, we can safely establish these two QTL genes as casual genetic variants for atherogenic diet-induced obesity in natural inbred mouse populations. It should be noted that evidence solely from knockout or transgenic mice is not sufficient to infer causal relationship between a particular gene and disease in natural populations since the gene that was knocked out or overexpressed in mice may be only one of the several genes involved in the same biological pathway leading to the disease, rather than the true gene that is mutated in natural populations. Only when genetic mapping data, especially from high-resolution QTL analyses that identify allelic variants associated with the disease, are incorporated with murine knockout or transgenic models can the casual relationship be firmly established.

### Gpr39 mapped in a pleiotropic QTL

**Figure 6:**
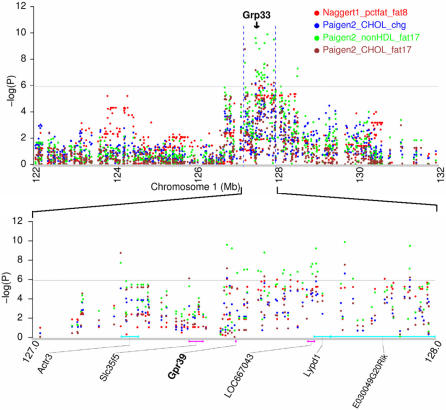
Gpr39 was mapped in a pleiotropic QTL. Gpr39 is associated with percent of tissue mass that is fat after 8 weeks on atherogenic diet (Naggert1_pctfat_fat8), total cholesterol after 17 weeks on atherogenic diet (Paigen2_CHOL_fat17), fold change in total cholesterol after 17 weeks on atherogenic diet (Paigen2_CHOL_chg), and non-HDL cholesterol after 17 wks on atherogenic diet (Paigen2_nonHDL_fat17). Gpr39 has knockout phenotypes on both obesity and blood cholesterol levels.

Among these 21 candidate genes (**Table 2** and **3**), Gpr39 has knockout phenotypes on both obesity and plasma lipid levels. Our GWA scans identified a QTL on chromosome 1 affecting percent fat mass on atherogenic diet (-log(P) = 6.1). This QTL colocalizes with another QTL identified by our GWA scans, which affects total cholesterol and non-HDL cholesterol levels, and fold change in total cholesterol levels after 17 weeks on atherogenic diet (-log(P) = 6.6–9.9) (**Figure 6**). The genomic locations of these two overlapped QTLs are indistinguishable which are very likely controlled by the same gene with pleiotropic effects on obesity and plasma lipid levels. The critical region of this pleiotropic QTL spans about 0.7 Mb (127.2–127.9 Mb) and covers six annotated genes based on the latest NCBI mouse genome build 36.1, including Actr3, Slc35f5, Gpr39, LOC667043, Lypd1 and E030049G20Rik. Gpr39 is a member of a family that includes the receptors for ghrelin and motilin [26]. Mature body weight and percent fat mass were increased by 38% and 5%, respectively, in Gpr39 loss-of-function (Gpr39^−/−^) mice; cholesterol levels were increased by 11% and 42% in male and female Gpr39^−/−^ mice, respectively [27]. This establishes Gpr39 as a candidate for this pleiotropic QTL affecting both obesity and plasma lipid levels, even in light of the fact of recent observations clouding its role [28]–[31]. Nevertheless, these remarkable phenotypic differences between Gpr39^−/−^ and wild-type mice mentioned above were only observed at old age (about 50 weeks for obesity and 25 weeks for cholesterol) [27], suggesting that deleterious mutations in Gpr39 may increase the risk for late-onset obesity and atherosclerotic coronary heart disease.

## Discussion

We have demonstrated the robustness and reproducibility of GWA analysis by comparing QTLs derived from GWA with those derived from previous linkage analyses in inbred mice. In a recent GWA analysis of lung tumor incidence, we reproduced the pulmonary adenoma susceptibility 1 (Pas1) locus identified in previous linkage studies and further narrowed this QTL to a region of less than 0.5 Mb in which at least two genes, Kras2 and Casc1, are strong candidates [4]. The refined region is completely coincident with that from traditional fine mapping by using congenic strains of mice [32]. Casc1 knockout mouse tumor bioassays further confirmed Casc1 as a primary candidate for the *Pas1* locus [4]. In the present study, we again reproduced and narrowed the Bwtq1 locus to a region of less than 1.0 Mb where Pappa2 is a primary candidate. The refined Bwtq1 region is identical to that achieved by developing a series of congenic mice and interval-specific subcongenic mice [13], [14]. These results demonstrate that GWA scans using dense SNP maps in laboratory mice are a powerful tool for the refinement of previously identified QTL regions. These results can be also served as an internal control for the methods that we used in our GWA scans. Given that known refined QTLs were exactly reproduced in the analyses, the newly refined QTLs identified by our GWA scans are expected to be a useful resource for further positional cloning and gene discovery in the mouse. It should be also noted that 937 QTLs identified in our study represent about one half of all QTLs derived from linkage analyses in mouse cross-breeding experiments in the past decades (http://www.informatics.jax.org).

Research tools such as genetrap mutations, transgenes, and standard knockout technology can be used to verify causal variants from highly-refined QTL regions immediately after GWA scans [33], [34]. Using resources from murine gene-deficiency and/or transgenic models, we have identified 10 candidate genes affecting obesity-related phenotypes and 11 candidate genes affecting plasma cholesterol levels from a survey of several highly refined QTLs (**Table 2** and **3**). These QTL genes are most likely the causal genes based on the following evidence. First, the genomic regions where these genes are located are not only highly associated with the analyzed phenotypes in the GWA scans, but are also under the linkage peak from two or more independent linkage studies with crossing-breeding experiments [10], [22]. Second, these candidates were identified from a highly refined genomic region, as narrow as 0.54 Mb (with an average of 6 genes per region), which is extremely more precise than those chosen from a much broader linkage region (about 20 Mb). Third, and more importantly, relevant phenotypes for these genes were observed in murine knockout and/or transgenic models. Notably, due to the single-gene resolution achieved by our GWA scans, we firmly establish two QTL genes, Adam12 and Cdh2, as causal variants for obesity in inbred mice. It is also worth noting that in the present study we only focused on 28 obesity and cholesterol QTLs out of 937 QTLs, which have available data from murine knockout and/or transgenic models (**Table 2** and **3**). Other QTL genes identified in this study are now ready to be evaluated for their functional relevance to the analyzed mouse phenotypes. We have summarized this extremely valuable QTL resource in online **Table S1** and **S2**; this resource can greatly facilitate positional cloning and the identification of new genetic determinants of complex traits.

Finally, several caveats for our findings should be mentioned. Firstly, selection and inbreeding play important roles in the formation of the genetic architecture of mouse inbred strains. Potential population structure may arise in these mouse strains, which inflates type I error rates and may lead to spurious associatons in the analysis [3]. Wild-derived inbred mice have divergent evolutionary histories and thus have strong potential to generate spurious assocations. There are systematic differences in obesity-related trait values between wild-derived and other inbred strains. Therefore, the population structure was carefully inspected in our association analysis, and we removed wild-derived inbred strains from the analysis when population structure was detected. Spurious associations were largely reduced in the analysis after the removal of wild-derived inbred mouse strains (**Figure S7** and **Text S1**). Several methods have been developed that adjust for the effect of population structure on spurious assocations [35]–[38], but these may not be sufficient for the analysis of a small number of mouse inbred strains (∼30–40 strains) [39]. Secondly, we used permutations to establish stringent genome-wide thresholds for declaring significant association for each phenotype. Although most genomic regions show correct type I error rates, some regions may show increased error rates due to unequal relatedness among the strains [40]. The risk of these spurious associations can be alleviated when incorporating prior linkage evidence into the analysis. The identified QTL genes located within previous linkages that have been replicated in two or more independent studies should be prioritized for further investigation. The associated QTL alleles need to be checked to see if they are segregated in linkage mapping populations. We have demonstrated this strategy in a recent study in which the positional cloning of a novel QTL gene, from a previous linkage-defined region, was done immediately after a GWA analysis [4]. Thirdly, we estimated power of association analysis using classical inbred mouse strains through simulation studies (**Figure S8** and **Text S1**). GWA scans have reasonable power to detect QTL genes with major effects, while are limited to detect QTL genes with moderate effects. However, focusing association analysis on linkage-defined regions can dramatically increase statistical power and has sufficient power to detect moderate-effect QTL genes. In our results, the great majority of the QTLs identified in the GWA scans overlapped with previous linkage-defined regions from mouse crosses which were retrieved from the Mouse Genome Informatics (MGI) at http://www.informatics.jax.org. Nevertheless, association results for small-effect QTLs from non-linkage regions should be interpreted with caution. It should also be noted that statistical power of association analysis in inbred mice can be significantly improved by increasing the number of mice per strain used in phenotype measurement. Fourthly, most SNPs were discovered by comparison of the genomes of several classical inbred laboratory mouse strains (such as C57BL/6J, DBA/2J, A/J and 129S1/SvImJ). Very few SNPs show polymorphisms among wild-derived strains. The ascertainment bias of SNPs will affect population inference including linkage disequilibrium and population structure. This ascertainment bias will also likely erode the power of tests of association between genotype and phenotype. However it is unlikely that the ascertainment bias will introduce false-positive inferences [41].

## Methods

### Mouse phenotypes and SNP data

Mouse phenotypes were generated by the mouse PHENOME projects (http://phenome.jax.org/pub-cgi/phenome/mpdcgi?rtn = docs/home). In most PHENOME projects, each phenotype was measured for about 40 strains from a total of 59 inbred strains of mice. The strains used for phenotyping varied slightly among different projects. Generally, at least 10 males and 10 females from each strain were measured for each phenotype when 10–14 weeks of age. Before statistical analysis was conducted, the phenotypic data were subjected to detection of outliers using box plot (http://www.itl.nist.gov/div898/handbook/) (**Text S1**). The SNP data were obtained from the Wellcome Trust Centre for Human Genetics (WTCHG) at Oxford University (http://www.well.ox.ac.uk/mouse/INBREDS) and the Broad Institute of Harvard and MIT (http://www.broad.mit.edu/personal/claire/MouseHapMap/Inbred.htm). The genomic positions (in bp) of the SNPs for the two data sets were unified based on the latest NCBI mouse genome map build 36.1. These SNPs were further selected by removing SNPs with fewer than 20 strains typed or without genetic mapping information. The resulting SNP data include 148,062 SNPs spanning the mouse genome at an average density of ∼18 kb per SNP. The SNP genotyping accuracy reported by the WTCHG and the Broad Institute is over 99.8%. It is worth noting that several strains used in the analysis only have available WTCHG SNP data. Thirty-seven additional SNPs chosen from Perlegen Sciences were genotyped to fill gaps in QTL regions covering Adam12 and Cdh2.

### Whole-genome LD patterns

The commonly used pairwise LD measure, *r^2^*, which is the squared correlation coefficient of alleles at two loci [42], was calculated for pairwise SNPs on the mouse genome. To capture large-scale patterns of LD, we measured the number of proxies, i.e. SNPs showing a strong correlation with one or more others [9], with a window size of 500 kb across the genome.

### GWA scans

A full description of statistical methods for GWA scans is provided in **Text S1**. Briefly, the associations of a mouse phenotype with SNP markers on the genome were tested by using linear regression models. To maximize power in GWA scans, we examined several competitive models with different variables based on Bayesian information criteria. The log-likelihood-ratio test statistic for the existence of a QTL is calculated by comparing the likelihood values under full model with null model. In the results, the negative 10-base logarithmic p value from *x* ^2^ tests, i.e. –log(P), was presented. Population structure was investigated by the cumulative distribution of p values in genome-wide association analysis for each phenotype [35]. We removed wild-derived inbred strains in the association analysis when population structure was detected.

In the GWA scans, 1,000 permutations were used to establish a genome-wide threshold (a global p value of 0.05) for declaring significant associations for each phenotype. Specifically, the analyzed phenotype was randomly reshuffled among subjects while fixing the genotypes. For each of the 1,000 permutations, the GWA scan was repeated, and the most significant –log(P) was recorded. Sorting the maximum –log(P) from large to small, the 5% quantile of the empirical distribution was taken as the genome-wide threshold (a global p value of 0.05). Since LD extends a very short genomic region (normally less than a few hundred kb) on the mouse genome, once a significant SNP is identified, a causal variant responsible for the QTL should be close to the identified SNP. To define approximate QTL regions, we first identified significant SNPs with an established empirical threshold. If two significant SNPs were less than 1,000 kb from each other, they were treated as one association signal. Then these genomic regions were extended to cover closely linked SNPs that showed suggestive associations with the analyzed traits. We also reported p values, physical positions and genomic domains for each significant SNP. The identified QTLs were compared with those detected by previous linkage analyses from mouse crosses which were retrieved from Mouse Genome Informatics (MGI) at http://www.informatics.jax.org.

### Data transformation

To avoid the violation of assumption of normal distribution in the above statistical analysis, 173 quantitative phenotypes were checked for their normality by Shapiro-Wilk tests [43] before GWA scans. Phenotypes with Shapiro-Wilk tests P<0.05 were converted to approach normality by Box-Cox transformation or normal scores [44], [45]. For those phenotypes still showing marked departure from the normal distribution (p<0.01) after data transformation, the Mann-Whitney rank tests were performed in the GWA scans [46].

### Heritability

The heritability of inbred strains can be estimated by linear regression models with sex, strain and sex-strain interaction as independent variables. Each strain was treated as a categorical variable in the linear model analyses. The difference between the adjusted *R^2^* of the regression models with and without strain and its associated sex-strain interaction is approximately equal to the percentage of genetic variation (that is, heritability) in inbred mouse populations. Similarly, the phenotypic variation due to sex differences can also be estimated.

### Phylogenetic analysis

The phylogenetic tree was constructed with 148,062 SNPs from 59 inbred mouse strains, implemented in the *dnadist* program in the PHYLIP 3.66 package (http://evolution.genetics.washington.edu/phylip.html). Branch length information was used to plot evolutionary distance between strains in the *drawtree* program in the PHYLIP 3.66 package.

## Supporting Information

Text S1 Supporting methods: A detailed description of methods and materials.
(0.11 MB DOC)

Table S1 173 mouse quantitative traits analyzed in genome-wide association analyses. The table gives empirical genome-wide threshold, the number of QTLs and significant SNPs detected in GWA scans for each trait. The table also lists project names, mouse phenome database (MPD) accession numbers, short and full name of traits, and categories of traits.
(0.36 MB DOC)

Table S2 Summary of 937 QTL identified in genome-wide association analyses. The table gives the chromosome, position of QTL peak (bp, based on the NCBI genome build 36), QTL interval, and -log (P) for each QTL identified in GWA scans. The table also provides the SNP ID of the most significant SNP and its dbSNP's annotation if available, and total number of significant and suggestive SNPs for each QTL.
(1.99 MB DOC)

Figure S1 Distribution of SNP positions and LD structure across the mouse genome. For each chromosome, the top panel shows number of SNPs in a window size of 500 kb. The bottom panel shows number of proxies (red lines for rˆ2>0.5 and blue lines for rˆ2>0.8) per SNP in a window size of 500 kb.
(0.41 MB PDF)

Figure S2 Characteristics of the mouse SNP map. (A) Distribution of SNP allele frequency. (B) Distribution of inter-SNP distances. (C) LD decay as a function of physical distance. (D) Number of proxies per SNP in a window size of 500 kb, as a function of the threshold for correlation (rˆ2).
(0.90 MB PDF)

Figure S3 Phylogenetic tree of 59 inbred mouse strains. The phylogenetic tree was constructed with a total of 148,062 SNPs from the WTCHG and Broad Institute, implemented in the dnadist program in the PHYLIP 3.66 package (http://evolution.genetics.washington.edu/phylip.html). The branch length information was used to plot evolutionary distance between strains in the drawtree program. 59 inbred mouse strains are organized into seven groups: Bagg albino derivatives, C57-related strains, Castle's mice, Japanese and New Zealand inbred strains, Little's DBA and related strains, Swiss mice, and wild-derived strains.
(1.41 MB PDF)

Figure S4 Characteristics of 173 complex quantitative traits in inbred mice. (A) Heritability of quantitative traits. (B) Phenotypic variation due to sex effects.
(0.70 MB PDF)

Figure S5 An in silico strategy for high-throughput gene discovery in inbred mice. GWA scans were implemented in an automatic processing pipeline which constitutes data retrieving, outlier detection, data preprocessing, hypothesis testing, permutations and QTL identification.
(0.21 MB PDF)

Figure S6 Genome-wide association analysis of several obesity-related phenotypes in inbred mice. The obesity-relaed phenotypes are (A) body weight at the start of testing (8 weeks) (Tordoff3_bw_start); (B) calculated weight of lean tissue (14 weeks) (Tordoff3_lean_wt); (C) body weight after 8 weeks on an atherogenic diet (Naggert1_bw_fat8); (D) total tissue mass after 8 weeks on an atherogenic diet (Naggert1_tissuemass_fat8); (E) weight of lean portion of tissue mass after 8 weeks on an atherogenic diet (Naggert1_leanwt_fat8); (F) bone mineral content after 8 weeks on an atherogenic diet (Naggert1_BMC_fat8); (G) initial body weight (7–9 weeks), day 0 of an atherogenic diet (Paigen1_initbw); and (H) final body weight after 8 weeks on an atherogenic diet (Paigen1_finalbw). In Paigen1 and Naggert1 projects, mice at 7–9 weeks of age were weighed and then administered a high fat, high cholesterol atherogenic diet. The scatter plots were drawn for -log (P) against the SNP position in the chromosomes. The horizontal gray lines indicate genome-wide empirical thresholds (global p value = 0.05). The horizontal coordinates were plotted using physical distance (Mb) in each panel.
(7.96 MB PDF)

Figure S7 Potential population structure in inbred mice. Body weight after 8 weeks on altherogenic diet (Naggert1_bw_fat8) was used to illustrate the population structure in inbred mice. (A) Cummlative distribution of p values from GWA analysis of samples with (red line) and without (blue line) wild-derived inbred strains. cdf, cumulative distribution function. (B) Comparison of GWA analysis of samples with (upper, red) and without (lower, blue) wild-derived inbred strains. The two green horizontal lines are genome-wide thresholds (a global p value of 0.05). Spurious assocaitons were largely reduced in the analysis after the removal of wild-derived inbred mouse strains.
(1.78 MB PDF)

Figure S8 Power of association analysis in inbred mice. Power was estimated for different trait heritabilities under genome-wide and QTL-wide thresholds (P = 0.05). The genome-wide threshold is used to declare a significant association on the genome without any prior genetic evidence; while the QTL-wide threshold is used to declare a significant association on a privious linkage-defined region.
(0.48 MB PDF)

## References

[1] Waterston RH, Lindblad-Toh K, Birney E, Rogers J, Abril JF (2002). Initial sequencing and comparative analysis of the mouse genome.. Nature.

[2] Peters LL, Robledo RF, Bult CJ, Churchill GA, Paigen BJ (2007). The mouse as a model for human biology: a resource guide for complex trait analysis.. Nat Rev Genet.

[3] Wade CM, Daly MJ (2005). Genetic variation in laboratory mice.. Nat Genet.

[4] Liu P, Wang Y, Vikis H, Maciag A, Wang D (2006). Candidate lung tumor susceptibility genes identified through whole-genome association analyses in inbred mice.. Nat Genet.

[5] Liao G, Wang J, Guo J, Allard J, Cheng J (2004). In silico genetics: identification of a functional element regulating H2-Ealpha gene expression.. Science.

[6] Grupe A, Germer S, Usuka J, Aud D, Belknap JK (2001). In silico mapping of complex disease-related traits in mice.. Science.

[7] Pletcher MT, McClurg P, Batalov S, Su AI, Barnes SW (2004). Use of a dense single nucleotide polymorphism map for in silico mapping in the mouse.. PLoS Biol.

[8] Valdar W, Solberg LC, Gauguier D, Burnett S, Klenerman P (2006). Genome-wide genetic association of complex traits in heterogeneous stock mice.. Nat Genet.

[9] Altshuler D, Brooks LD, Chakravarti A, Collins FS, Daly MJ (2005). A haplotype map of the human genome.. Nature.

[10] Rankinen T, Zuberi A, Chagnon YC, Weisnagel SJ, Argyropoulos G (2006). The human obesity gene map: the 2005 update.. Obesity (Silver Spring).

[11] Morris KH, Ishikawa A, Keightley PD (1999). Quantitative trait loci for growth traits in C57BL/6J×DBA/2J mice.. Mamm Genome.

[12] Christians JK, Bingham VK, Oliver FK, Heath TT, Keightley PD (2003). Characterization of a QTL affecting skeletal size in mice.. Mamm Genome.

[13] Christians JK, Keightley PD (2004). Fine mapping of a murine growth locus to a 1.4-cM region and resolution of linked QTL.. Mamm Genome.

[14] Christians JK, Hoeflich A, Keightley PD (2006). PAPPA2, an enzyme that cleaves an insulin-like growth-factor-binding protein, is a candidate gene for a quantitative trait locus affecting body size in mice.. Genetics.

[15] Brockmann GA, Haley CS, Renne U, Knott SA, Schwerin M (1998). Quantitative trait loci affecting body weight and fatness from a mouse line selected for extreme high growth.. Genetics.

[16] Brockmann GA, Kratzsch J, Haley CS, Renne U, Schwerin M (2000). Single QTL effects, epistasis, and pleiotropy account for two-thirds of the phenotypic F(2) variance of growth and obesity in DU6i×DBA/2 mice.. Genome Res.

[17] Cheverud JM, Vaughn TT, Pletscher LS, Peripato AC, Adams ES (2001). Genetic architecture of adiposity in the cross of LG/J and SM/J inbred mice.. Mamm Genome.

[18] Vaughn TT, Pletscher LS, Peripato A, King-Ellison K, Adams E (1999). Mapping quantitative trait loci for murine growth: a closer look at genetic architecture.. Genet Res.

[19] Bymaster FP, McKinzie DL, Felder CC, Wess J (2003). Use of M1-M5 muscarinic receptor knockout mice as novel tools to delineate the physiological roles of the muscarinic cholinergic system.. Neurochem Res.

[20] Yamada M, Miyakawa T, Duttaroy A, Yamanaka A, Moriguchi T (2001). Mice lacking the M3 muscarinic acetylcholine receptor are hypophagic and lean.. Nature.

[21] Brewer HBJ, Santamarina-Fojo S, Shamburek RD, Fuster V, Topol EJ, Nabel EG (2005). Genetic dyslipoproteinemias.. Atherothrombosis and coronary artery disease.

[22] Rollins J, Chen Y, Paigen B, Wang X (2006). In search of new targets for plasma high-density lipoprotein cholesterol levels: promise of human-mouse comparative genomics.. Trends Cardiovasc Med.

[23] Weng W, Breslow JL (1996). Dramatically decreased high density lipoprotein cholesterol, increased remnant clearance, and insulin hypersensitivity in apolipoprotein A-II knockout mice suggest a complex role for apolipoprotein A-II in atherosclerosis susceptibility.. Proc Natl Acad Sci U S A.

[24] Masaki M, Kurisaki T, Shirakawa K, Sehara-Fujisawa A (2005). Role of meltrin {alpha} (ADAM12) in obesity induced by high- fat diet.. Endocrinology.

[25] Castro CH, Shin CS, Stains JP, Cheng SL, Sheikh S (2004). Targeted expression of a dominant-negative N-cadherin in vivo delays peak bone mass and increases adipogenesis.. J Cell Sci.

[26] Zhang JV, Ren PG, Avsian-Kretchmer O, Luo CW, Rauch R (2005). Obestatin, a peptide encoded by the ghrelin gene, opposes ghrelin's effects on food intake.. Science.

[27] Moechars D, Depoortere I, Moreaux B, de Smet B, Goris I (2006). Altered gastrointestinal and metabolic function in the GPR39-obestatin receptor-knockout mouse.. Gastroenterology.

[28] Chartrel N, Alvear-Perez R, Leprince J, Iturrioz X, Reaux-Le Goazigo A (2007). Comment on “Obestatin, a peptide encoded by the ghrelin gene, opposes ghrelin's effects on food intake”.. Science.

[29] Holst B, Egerod KL, Schild E, Vickers SP, Cheetham S (2007). GPR39 signaling is stimulated by zinc ions but not by obestatin.. Endocrinology.

[30] Lauwers E, Landuyt B, Arckens L, Schoofs L, Luyten W (2006). Obestatin does not activate orphan G protein-coupled receptor GPR39.. Biochem Biophys Res Commun.

[31] Tremblay F, Perreault M, Klaman LD, Tobin JF, Smith E (2007). Normal food intake and body weight in mice lacking the G protein-coupled receptor GPR39.. Endocrinology.

[32] Zhang Z, Futamura M, Vikis HG, Wang M, Li J (2003). Positional cloning of the major quantitative trait locus underlying lung tumor susceptibility in mice.. Proc Natl Acad Sci U S A.

[33] Flaherty L, Herron B, Symula D (2005). Genomics of the future: identification of quantitative trait loci in the mouse.. Genome Res.

[34] Nadeau JH, Frankel WN (2000). The roads from phenotypic variation to gene discovery: mutagenesis versus QTLs.. Nat Genet.

[35] Zhao K, Aranzana MJ, Kim S, Lister C, Shindo C (2007). An Arabidopsis Example of Association Mapping in Structured Samples.. PLoS Genet.

[36] Yu J, Pressoir G, Briggs WH, Vroh Bi I, Yamasaki M (2006). A unified mixed-model method for association mapping that accounts for multiple levels of relatedness.. Nat Genet.

[37] Pritchard JK, Stephens M, Donnelly P (2000). Inference of population structure using multilocus genotype data.. Genetics.

[38] Devlin B, Roeder K (1999). Genomic control for association studies.. Biometrics.

[39] Marchini J, Cardon LR, Phillips MS, Donnelly P (2004). The effects of human population structure on large genetic association studies.. Nat Genet.

[40] Payseur BA, Place M (2007). Prospects for Association Mapping in Classical Inbred Mouse Strains.. Genetics.

[41] Clark AG, Hubisz MJ, Bustamante CD, Williamson SH, Nielsen R (2005). Ascertainment bias in studies of human genome-wide polymorphism.. Genome Res.

[42] Hedrick PW (1987). Gametic disequilibrium measures: proceed with caution.. Genetics.

[43] Royston P (1982). An Extension of Shapiro and Wilk's W Test for Normality to Large Samples.. Applied Statistics.

[44] Altman DG (1991). Practical Statistics for Medical Research: Chapman and Hall..

[45] Box GEP, Cox DR (1964). An analysis of transformations.. Journal of Royal Statistical Society, Series B.

[46] Hollander M, Wolfe DA (1973). Nonparametric statistical inference..

[47] Bale TL, Anderson KR, Roberts AJ, Lee KF, Nagy TR (2003). Corticotropin-releasing factor receptor-2-deficient mice display abnormal homeostatic responses to challenges of increased dietary fat and cold.. Endocrinology.

[48] Naaz A, Holsberger DR, Iwamoto GA, Nelson A, Kiyokawa H (2004). Loss of cyclin-dependent kinase inhibitors produces adipocyte hyperplasia and obesity.. Faseb J.

[49] Font de Mora J, Esteban LM, Burks DJ, Nunez A, Garces C (2003). Ras-GRF1 signaling is required for normal beta-cell development and glucose homeostasis.. Embo J.

[50] Tuncman G, Hirosumi J, Solinas G, Chang L, Karin M (2006). Functional in vivo interactions between JNK1 and JNK2 isoforms in obesity and insulin resistance.. Proc Natl Acad Sci U S A.

[51] Longo KA, Wright WS, Kang S, Gerin I, Chiang SH (2004). Wnt10b inhibits development of white and brown adipose tissues.. J Biol Chem.

[52] Wu JJ, Roth RJ, Anderson EJ, Hong EG, Lee MK (2006). Mice lacking MAP kinase phosphatase-1 have enhanced MAP kinase activity and resistance to diet-induced obesity.. Cell Metab.

[53] Goudriaan JR, Dahlmans VE, Teusink B, Ouwens DM, Febbraio M (2003). CD36 deficiency increases insulin sensitivity in muscle, but induces insulin resistance in the liver in mice.. J Lipid Res.

[54] Miyasaka K, Takata Y, Funakoshi A (2002). Association of cholecystokinin A receptor gene polymorphism with cholelithiasis and the molecular mechanisms of this polymorphism.. J Gastroenterol.

[55] Patel HR, Qi Y, Hawkins EJ, Hileman SM, Elmquist JK (2006). Neuropeptide Y deficiency attenuates responses to fasting and high-fat diet in obesity-prone mice.. Diabetes.

[56] Akiyama TE, Sakai S, Lambert G, Nicol CJ, Matsusue K (2002). Conditional disruption of the peroxisome proliferator-activated receptor gamma gene in mice results in lowered expression of ABCA1, ABCG1, and apoE in macrophages and reduced cholesterol efflux.. Mol Cell Biol.

[57] Weisberg SP, Hunter D, Huber R, Lemieux J, Slaymaker S (2006). CCR2 modulates inflammatory and metabolic effects of high-fat feeding.. J Clin Invest.

[58] Mao J, DeMayo FJ, Li H, Abu-Elheiga L, Gu Z (2006). Liver-specific deletion of acetyl-CoA carboxylase 1 reduces hepatic triglyceride accumulation without affecting glucose homeostasis.. Proc Natl Acad Sci U S A.

[59] Huang LS, Voyiaziakis E, Markenson DF, Sokol KA, Hayek T (1995). apo B gene knockout in mice results in embryonic lethality in homozygotes and neural tube defects, male infertility, and reduced HDL cholesterol ester and apo A-I transport rates in heterozygotes.. J Clin Invest.

[60] Buhman KK, Accad M, Novak S, Choi RS, Wong JS (2000). Resistance to diet-induced hypercholesterolemia and gallstone formation in ACAT2-deficient mice.. Nat Med.

[61] Ishida T, Choi S, Kundu RK, Hirata K, Rubin EM (2003). Endothelial lipase is a major determinant of HDL level.. J Clin Invest.

