# Supplementary material for: Large-Scale In Silico Mapping of Complex Quantitative Traits in Inbred Mice
Source: PLoS One. 2007 Jul 25;2(7):e651. doi: 10.1371/journal.pone.0000651 (PMC1920557; doi:10.1371/journal.pone.0000651)
Supplement: Text S1 — Supporting methods: A detailed description of methods and materials. (0.11 MB DOC) [file pone.0000651.s001.doc]

**Supporting Methods**

**Mouse phenotypes.** Mouse phenotypes were generated by the PHENOME projects and downloaded from the Mouse Phenome Database (MPD) (http://phenome.jax.org/pub-cgi/phenome/mpdcgi?rtn=docs/home). A detailed list of investigators of the PHENOME projects is provided at the end of this section. In most PHENOME projects, each phenotype was measured for about 40 strains from a total of 59 inbred strains of mice. The strains used for phenotyping varied slightly among different PHENOME projects. These strains included 129S1/SvImJ, 129X1/SvJ, A/J, AKR/J, ALR/LtJ, ALS/LtJ, BALB/cByJ, BALB/cJ, BPH/2J, BPL/1J, BPN/3J, BTBR_T+_tf/J, BUB/BnJ, C3HeB/FeJ, C3H/HeJ, C57BL/10J, C57BL/6J, C57BLKS/J, C57BR/cdJ, C57L/J, C58/J, CAST/EiJ, CBA/CaJ, CBA/J, CE/J, CZECHII/EiJ, DBA/1J, DBA/2J, FVB/NJ, I/LnJ, JF1/Ms, KK/HlJ, LG/J, LP/J, MA/MyJ, MOLF/EiJ, MRL/MpJ, MSM/Ms, NOD/LtJ, NON/LtJ, NOR/LtJ, NZB/BlNJ, NZW/LacJ, PERA/EiJ, P/J, PL/J, RBF/DnJ, RF/J, RIIIS/J, SEA/GnJ, SEC/1ReJ, SENCARA/PtJ, SJL/J, SM/J, SPRET/EiJ, ST/bJ, SWR/J, WSB/EiJ and YBR/EiJ. Generally, at least 10 males and 10 females from each strain were measured for each phenotype when 10-14 weeks of age. Before statistical analysis was conducted, the phenotypic data were subjected to detection of outliers using box plot (http://www.itl.nist.gov/div898/handbook/). The box plot was constructed by drawing a box between the upper and lower quartiles, with a solid line drawn across the box to locate the median. The following quantities were calculated in order to identify extreme values in the tails of the distribution: 1) Lower inner fence: Q1 – 1.5IQ; 2) Upper inner fence: Q1 + 1.5IQ; 3) Lower outer fence: Q1 – 3IQ; and 4) Upper outer fence: Q1 + 3IQ, where Q1 and Q2 are the 25^th^ and 75^th^ percentiles and IQ = Q2 – Q1. We used inner fences to detect mild outliers within each strain and used outer fences to detect extreme outliers among strains in the samples.

**SNP data.** The SNP data were obtained from the Wellcome Trust Centre for Human Genetics at Oxford Univeristy (WTCHG) (http:// www.well.ox.ac.uk/mouse/INBREDS) and the Broad Institute of Harvard and MIT and the Massachusetts General Hospital (http:// www.broad.mit.edu/personal/claire/MouseHapMap/Inbred.htm). In initial data sets, the WTCHG SNP database contained 13,374 SNPs and the Broad SNP database contained 138,793 SNPs in commonly used strains. The genomic positions (in bp) of the SNPs for the two data sets were unified based on the latest NCBI mouse genome map build 36.1. These SNPs were further selected by removing SNPs with fewer than 20 strains typed or without genetic mapping information. The resulting SNP data include 148,062 SNPs spanning the mouse genome at an average density of ~18 kb per SNP. The SNP genotyping accuracy reported by the WTCHG and the Broad Institute is over 99.8%. It is worth noting that several strains used in the analysis only have available WTCHG SNP data. Thirty-seven additional SNPs chosen from Perlegen Sciences were genotyped to fill gaps in QTL regions covering Adam12 and Cdh2.

**Genome-wide LD patterns.** The commonly used pairwise LD measure, *r^2^*, which is the squared correlation coefficient of alleles at two loci [1], was calculated for pairwise SNPs on the mouse genome. To capture large-scale patterns of LD, we measured the number of proxies, i.e. SNPs showing a strong correlation with one or more others [2], with a window size of 500 kb across the genome.

**GWA scans.** The associations of a mouse phenotype with SNP markers on the genome were tested by using a linear model,

$y=\mu+\sum x_{i}\beta_{i}+e$ (1)

where $y$ is the trait value of a mouse inbred strain in the population, $\mu$ is the mean in the model, $x_{i}$ is a covariate (e.g. sex and additive effect), $\beta_{i}$ is a regression coefficient associated with $x_{i}$, and $e$ is the random residual. We first examined if there is a sex effect on the trait by using paired Student *t* tests. In the paired *t* tests, male and female mice from the same mouse strains were treated as a pair. If there was no sex effect, the above model was reduced to,

$y=\mu+x_{a}\beta_{a}+e$ (2)

where $\beta_{a}$ is the regression coefficient of additive effect, and $x_{a}$ takes a value of -1 for SNP genotype *M_1_* and 1 for SNP genotype *M_2_*. The log-likelihood-ratio (LR) test statistic for the existence of a QTL is calculated by comparing the likelihood values under the above full model with the null model,

$y=\mu+e$ (3)

LR follows asymptotically a$\chi^{2}$distribution with 1 degree of freedom (df). If there is a sex effect, three possible models are considered. First, the QTL has the same effect in both females and males,

$y=\mu+x_{s}\beta_{s}+x_{a}\beta_{a}+e$ (4)

where $\beta_{s}$ is the regression coefficient for sex effect, and $x_{s}$ takes a value of -1 for females and 1 for males. Second, the QTL also exhibits different effects on the trait in females and males,

$y=\mu+x_{s}\beta_{s}+x_{a}\beta_{a}+x_{sa}\beta_{sa}+e$ (5)

where $\beta_{sa}$ is the regression coefficient for sex-gene interaction, and $x_{sa}=x_{s}x_{a}$. Third, it is possible that the QTL does not have main effect but only has sex-gene interaction,

$y=\mu+x_{s}\beta_{s}+x_{sa}\beta_{sa}+e$ (6)

The existence of a QTL can be examined by comparing the likelihood values under models (4), (5) and (6) with the null model,

$y=\mu+x_{s}\beta_{s}+e$ (7)

The LR has 1 df for model (4) *versus* (7), 2 df for model (5) *versus* (7), and 1 df for model (6) *versus* (7).

The full model (5) allows us to simultaneously model sex effect, genetic effect and sex-gene interaction in a single linear model. Furthermore, the use of the full model (5) obviates the need for modeling QTL in one sex only, a procedure that could decrease the power by halving the sample size, rendering it impossible to detect sex-gene interactions. However, the full model (5) has 2 df for the LR statistic. If there is no significant main genetic effect or sex-gene interaction, the full model (5) actually reduces the power to detect association. To minimize the loss in power of fitting the full model (5), we employed Bayesian information criteria (BIC) to choose the most fitting one among the models (4), (5) and (6),

$BIC=-2\ln L+k\ln(n)$ (8)

where *L* is the maximized value of the likelihood function for the estimated model, *k* is the number of free parameters to be estimated, and *n* is the sample size. Given any two estimated models, the model with the smaller BIC value is the one to be preferred. The chosen model is then compared to the null model (7) for testing the existence of a QTL. In the results, the negative 10-base logarithmic p value from $\chi^{2}$ tests, i.e. –log(P), was presented.

One potential problem that may arise in our association analysis is population structure that inflates type I errors and may lead to spurious associations [3]. Population structure was investigated by the cumulative distribution of p values in genome-wide association analysis for each phenotype [4]. We removed wild-derived inbred strains in the association analysis when population structure was detected. Spurious associations were largely reduced in the analysis after the removal of wild-derived inbred mouse strains.

**Data transformation.** To avoid the violation of assumption of normal distribution in the above statistical analysis, 173 quantitative phenotypes were checked for their normality by Shapiro-Wilk tests [5] before GWA scans. Phenotypes with Shapiro-Wilk tests P <0.05 were converted to approach normality by Box-Cox transformation or normal scores [6,7]. For those phenotypes still showing marked departure from the normal distribution (p < 0.01) after data transformation, the Mann-Whitney rank tests were performed in the GWA scans [8].

**Genome-wide threshold.** In the GWA scans, 1,000 permutations were used to establish a genome-wide threshold (a global p value of 0.05) for declaring significant associations for each phenotype. Specifically, the analyzed phenotype was randomly reshuffled among subjects while fixing the genotypes. For each of the 1,000 permutations, the GWA scan was repeated, and the most significant –log(P) was recorded. Sorting the maximum –log(P) from large to small, the 5% quantile of the empirical distribution was taken as the genome-wide threshold (a global p value of 0.05). Since LD extends a very short genomic region (normally less than a few hundred kb) on the mouse genome, once a significant SNP is identified, a causal variant responsible for the QTL should be close to the identified SNP. To define approximate QTL regions, we first identified significant SNPs with an established empirical threshold. If two significant SNPs were less than 1,000 kb from each other, they were treated as one association signal. Then these genomic regions were extended to cover closely linked SNPs that showed suggestive associations with the analyzed traits. We also reported p values, physical positions and genomic domains for each significant SNP. The identified QTLs were compared with those detected by previous linkage analyses from mouse crosses which were retrieved from Mouse Genome Informatics (MGI) at [http://www.informatics.jax.org](http://www.informatics.jax.org/).

**Heritability.** The heritability of inbred strains can be estimated by linear regression models with sex, strain and sex-strain interaction as independent variables. Each strain was treated as a categorical variable in the linear model analyses. The difference between the adjusted *R^2^* of the regression models with and without strain and its associated sex-strain interaction is approximately equal to the percentage of genetic variation (that is, heritability) in inbred mouse populations. Similarly, the phenotypic variation due to sex differences can also be estimated.

**Phylogenetic analysis.** The phylogenetic tree was constructed with 148,062 SNPs from 59 inbred mouse strains, implemented in the *dnadist* program in the PHYLIP 3.66 package (<http://evolution.genetics.washington.edu/phylip.html>). Branch length information was used to plot evolutionary distance between strains in the *drawtree* program in the PHYLIP 3.66 package.

**Power calculation.** Thirty-four classical inbred mouse strains were used for simulations of association analysis: 129S1/SvImJ, A/J, AKR/J, BALB/cByJ, BTBR_T+_tf/J, BUB/BnJ, C3H/HeJ, C57BL/6J, C57BLKS/J, C57BR/cdJ, C57L/J, C58/J, CBA/J, CE/J, DBA/1J, DBA/2J, FVB/NJ, I/LnJ, KK/HlJ, LG/J, LP/J, MA/MyJ, NOD/LtJ, NON/LtJ, NZB/BlNJ, NZW/LacJ, O20, PL/J, RIIIS/J, SEA/GnJ, SJL/J, SM/J, ST/bJ and SWR/J. SNPs with fewer than 30 strains typed or without genetic mapping information were removed. Phenotypes for individual strains were created from SNP genotypes [9]. Specifically, we randomly chose a SNP on the genome as a functional mutation, and assigned it with a trait heritability (*h^2^*). The chosen SNP was assumed to affect phenotype variation in an additive mode of inheritance and has a minor allele frequency > 0.1. We also assumed that total heritability of a simulated phenotype is 0.5, and 10 mice for each strain were measured for the simulated phenotype and the mean phenotypic value is used for association analysis. For a given heritability due to the functional SNP, its allelic effect was estimated as $\alpha=\sqrt{V_{P}h^{2}/4f(1-f)}$, where *Vp* is the total phenotypic variance in the inbred mouse population, and *f* is the minor allele frequency of the functional SNP. The random effects would include polygenic genetic variations and random environmental effects. Then similar statistical analysis as described above, was used to test associations of the simulated phenotype with SNPs in the whole genome or linkage-defined region. The simulated causal SNP was excluded for the association analysis. For GWA scans, we used genome-wide threshod (P = 0.05) that was established by permutation analysis to declare a significant association. Similarly, for QTL-wide association scans, we restricted the association analysis in a randomly chosen genomic region of 20 Mb where a SNP was simulated as a functional mutation, and QTL-wide threshold (P = 0.05) was used. Given the established threshold, a significant SNP that is within 500 kb from the functional SNP is considered a true association finding. A total of 5,000 simulations were performed to estimate power of association analysis under different trait heritabilities.

**Investigators of the PHENOME projects generated mouse phenotypes used for analysis in this study.** All phenotypes were downloaded from the Mouse Phenome Database Web Site, The Jackson Laboratory, Bar Harbor, Maine, USA, World Wide Web (URL: <http://jax.org/phenome>, June 2006).

1. Hunter KW. Genetic susceptibility to metastatic progression of a mammary tumor. MPD:60. *Hunter1* (1998).
2. The Jackson Laboratory. Reproductive performance survey. MPD:149. *Jax3* (1991).
3. Johnson KR, Erway LC, Zheng QY. Hearing assessment and identification of a gene affecting age-related hearing loss. MPD:14. *Johnson1* (2000).
4. Naggert JK, Svenson KL, Smith RV, Paigen B, Peters LL. Diet effects on bone mineral density and content, body composition, and plasma glucose, leptin, and insulin levels. MPD:143. *Naggert1* (2003).
5. Paigen B, Bouchard G, Carey M. Diet-induced disease: Gallstones, liver pathology, plasma lipids, and pathogen-accelerated atherosclerosis. MPD:29. *Paigen1* (2000).
6. Paigen B, Svenson KL, Peters LL. Diet effects on plasma lipids and susceptibility to atherosclerosis (pathogen-free conditions). MPD:99. *Paigen2* (2002).
7. Paigen B, Svenson KL, Peters LL. Plasma lipids in mice on 6% fat diet. MPD:197. *Paigen4* (2002).
8. Peters LL, Barker JE. Hematology, clotting, and thrombosis. MPD:62. *Peters1* (2001).
9. Tordoff MG, Bachmanov AA. Taste preferences. MPD:61. *Tordoff1* (2001).
10. Tordoff MG, Bachmanov AA. Food intakes, water intakes, and spout side preferences. MPD:63. *Tordoff2* (2001).
11. Tordoff MG, Bachmanov AA. Survey of calcium & sodium intake and metabolism with bone and body composition data. MPD:103. *Tordoff3* (2002).
12. Willott JF, Johnson KR. Startle and prepulse inhibition. MPD:91. *Willott1* (2002).

**References**

1. Hedrick PW (1987) Gametic disequilibrium measures: proceed with caution. Genetics 117: 331-341.

2. Altshuler D, Brooks LD, Chakravarti A, Collins FS, Daly MJ, et al. (2005) A haplotype map of the human genome. Nature 437: 1299-1320.

3. Wade CM, Daly MJ (2005) Genetic variation in laboratory mice. Nat Genet 37: 1175-1180.

4. Zhao K, Aranzana MJ, Kim S, Lister C, Shindo C, et al. (2007) An Arabidopsis Example of Association Mapping in Structured Samples. PLoS Genet 3: e4.

5. Royston P (1982) An Extension of Shapiro and Wilk’s W Test for Normality to Large Samples. Applied Statistics 31: 115-124.

6. Altman DG (1991) Practical Statistics for Medical Research: Chapman and Hall.

7. Box GEP, Cox DR (1964) An analysis of transformations. Journal of Royal Statistical Society, Series B 26: 211-246.

8. Hollander M, Wolfe DA (1973) Nonparametric statistical inference. New York: John Wiley and Sons. 68-75 p.

9. Liu P, Wang Y, Vikis H, Maciag A, Wang D, et al. (2006) Candidate lung tumor susceptibility genes identified through whole-genome association analyses in inbred mice. Nat Genet 38: 888-895.
