# Supplementary material for: Large-Scale In Silico Mapping of Complex Quantitative Traits in Inbred Mice
Source: PLoS One. 2007 Jul 25;2(7):e651. doi: 10.1371/journal.pone.0000651 (PMC1920557; doi:10.1371/journal.pone.0000651)
Supplement: Table S1 — 173 mouse quantitative traits analyzed in genome-wide association analyses. The table gives empirical genome-wide threshold, the number of QTLs and significant SNPs detected in GWA scans for each trait. The table also lists project names, mouse phenome database (MPD) accession numbers, short and full name of traits, and categories of traits. (0.36 MB DOC) [file pone.0000651.s002.doc]

**Table S1. 173 mouse quantitative traits analyzed in genome-wide association analyses**

| **Categories** | **Project** | **Acc#** | **Trait** | **Threshold** | **QTLs** | **SNPs** | **Full_name** |
| --- | --- | --- | --- | --- | --- | --- | --- |
| Blood biochemistry | Naggert1 | MPD:143 | GLU | 6.6 | 0 | 0 | glucose, before beginning atherogenic diet [mg/dL] |
| Blood biochemistry | Naggert1 | MPD:143 | GLU_fat17 | 6.4 | 13 | 44 | glucose, after 17 wks on atherogenic diet [mg/dL] |
| Blood biochemistry | Naggert1 | MPD:143 | GLU_foldchg_fat17 | 5.7 | 0 | 0 | fold change in glucose after 17 wks on atherogenic diet [fold] |
| Blood biochemistry | Naggert1 | MPD:143 | INS_fat18 | 6.6 | 0 | 0 | insulin, after 18 wks on atherogenic diet [ng/mL] |
| Blood biochemistry | Naggert1 | MPD:143 | LEP_fat18 | 5.9 | 36 | 206 | leptin, after 18 wks on atherogenic diet [pg/mL] |
| Blood biochemistry | Peters1 | MPD:62 | Fib | 6.3 | 0 | 0 | fibrinogen [mg/dL] |
| Blood biochemistry | Peters1 | MPD:62 | PT | 6.0 | 1 | 1 | prothrombin time [s] |
| Blood biochemistry | Peters1 | MPD:62 | PTT | 6.8 | 1 | 1 | partial thromboplastin time [s] |
| Blood biochemistry | Tordoff3 | MPD:103 | adj_ionized_Ca | 7.2 | 0 | 0 | blood ionized calcium adjusted to pH 7.4 [mmol/L] |
| Blood biochemistry | Tordoff3 | MPD:103 | bleeding_time | 6.8 | 3 | 8 | time from tail cut to 1/2 tube of blood collected (approx 30 uL) [s] |
| Blood biochemistry | Tordoff3 | MPD:103 | ionized_Ca | 7.1 | 0 | 0 | blood ionized calcium [mmol/L] |
| Blood biochemistry | Tordoff3 | MPD:103 | pH | 7.6 | 2 | 2 | blood pH [pH] |
| Blood biochemistry | Tordoff3 | MPD:103 | total_calcium | 7.5 | 3 | 22 | plasma total calcium [mmol/L] |
| Body composition | Naggert1 | MPD:143 | bw_fat8 | 6.5 | 8 | 17 | body weight after 8 wks on atherogenic diet [g] |
| Body composition | Naggert1 | MPD:143 | fatwt_fat8 | 6.4 | 17 | 49 | weight of fat portion of tissue mass after 8 wks on atherogenic diet [g] |
| Body composition | Naggert1 | MPD:143 | leanwt_fat8 | 6.4 | 4 | 7 | weight of lean portion of tissue mass after 8 wks on atherogenic diet [g] |
| Body composition | Naggert1 | MPD:143 | pctfat_fat8 | 6.0 | 31 | 130 | percent of tissue mass that is fat, after 8 wks on atherogenic diet [%] |
| Body composition | Naggert1 | MPD:143 | tissuemass_fat8 | 6.7 | 3 | 11 | total tissue mass after 8 wks on atherogenic diet [g] |
| Body composition | Paigen1 | MPD:29 | bw_chg | 5.9 | 1 | 1 | fold change in body weight after 8 wks on atherogenic diet [fold] |
| Body composition | Paigen1 | MPD:29 | finalbw | 6.6 | 6 | 32 | final body weight after 8 wks on atherogenic diet [g] |
| Body composition | Paigen1 | MPD:29 | initbw | 7.1 | 7 | 35 | initial body weight, day 0 of diet [g] |
| Body composition | Tordoff1 | MPD:61 | CaCl2_bwavg | 5.9 | 0 | 0 | average body weight during CaCl2 test [g] |
| Body composition | Tordoff1 | MPD:61 | KCl_bwavg | 5.6 | 0 | 0 | average body weight during KCl test [g] |
| Body composition | Tordoff1 | MPD:61 | NaCl_bwavg | 5.4 | 0 | 0 | average body weight during NaCl test [g] |
| Body composition | Tordoff1 | MPD:61 | NH4Cl_bwavg | 5.9 | 0 | 0 | average body weight during NH4Cl test [g] |
| Body composition | Tordoff2 | MPD:63 | bwavg | 5.4 | 0 | 0 | body weight average [g] |
| Body composition | Tordoff2 | MPD:63 | bwend | 5.4 | 0 | 0 | body weight at the end of the test [g] |
| Body composition | Tordoff2 | MPD:63 | bwstart | 5.3 | 0 | 0 | body weight at the beginning of the test [g] |
| Body composition | Tordoff3 | MPD:103 | bw_chg | 7.5 | 0 | 0 | fold change in body weight [fold] |
| Body composition | Tordoff3 | MPD:103 | bw_end | 7.7 | 0 | 0 | body weight at end of behavioral tests (before 1st blood sample) [g] |
| Body composition | Tordoff3 | MPD:103 | bw_start | 7.6 | 6 | 8 | body weight at start of testing [g] |
| Body composition | Tordoff3 | MPD:103 | fat_wt | 8.1 | 1 | 2 | calculated weight of fat tissue [g] |
| Body composition | Tordoff3 | MPD:103 | lean_wt | 7.1 | 5 | 7 | calculated weight of lean tissue [g] |
| Body composition | Tordoff3 | MPD:103 | pct_fat | 7.2 | 10 | 23 | percent fat [%] |
| Body composition | Tordoff3 | MPD:103 | pct_lean | 6.9 | 13 | 33 | percent lean [%] |
| Body composition | Tordoff3 | MPD:103 | total_wt | 8.2 | 0 | 0 | total weight (lean + fat) [g] |
| Body composition | Willott1 | MPD:91 | bw | 6.4 | 6 | 7 | body weight at age 5-6 wks [g] |
| Cancer | Hunter1 | MPD:60 | met_area | 6.6 | 0 | 0 | area of pulmonary metastases (x 103) [um2] |
| Cancer | Hunter1 | MPD:60 | met_size | 6.3 | 0 | 0 | area of pulmonary metastases per number of metastases [um2/n] |
| Cancer | Hunter1 | MPD:60 | mets | 7.8 | 0 | 0 | number of pulmonary metastases [n] |
| Cancer | Hunter1 | MPD:60 | ratio_ata | 7.0 | 0 | 0 | area of pulmonary metastases per total lung area |
| Cancer | Hunter1 | MPD:60 | ratio_mta | 5.8 | 1 | 1 | number of pulmonary metastases per total lung area (x 10-9) [n/um2] |
| Cancer | Hunter1 | MPD:60 | tot_area | 6.0 | 0 | 0 | total lung area (x 103) [um2] |
| Cancer | Hunter1 | MPD:60 | tum_burd | 6.4 | 1 | 1 | tumor burden (body weight at sacrifice minus body weight at diagnosis of primary tumor) [g] |
| Cancer | Hunter1 | MPD:60 | tum_lat | 6.0 | 0 | 0 | mammary tumor latency, days after birth [d] |
| Cholesterol | Paigen1 | MPD:29 | estHCC | 6.7 | 8 | 14 | hepatic cholesterol concentration, after 8 wks on atherogenic diet (esterified per g liver) [mg] |
| Cholesterol | Paigen1 | MPD:29 | free_to_est | 6.5 | 8 | 12 | hepatic cholesterol concentration, after 8 wks on atherogenic diet (free to esterified ratio) [mg/mg] |
| Cholesterol | Paigen1 | MPD:29 | freeHCC | 5.9 | 32 | 126 | hepatic cholesterol concentration, after 8 wks on atherogenic diet (free per g liver) [mg] |
| Cholesterol | Paigen1 | MPD:29 | HDL_Ch | 6.4 | 6 | 7 | HDL cholesterol (plasma), after 8 wks on atherogenic diet [mg/dL] |
| Cholesterol | Paigen1 | MPD:29 | nonHDL_Ch | 6.6 | 5 | 5 | plasma cholesterol not in the HDL fraction, after 8 wks on atherogenic diet [mg/dL] |
| Cholesterol | Paigen1 | MPD:29 | pctHDL-TCH | 6.7 | 14 | 43 | percent of total plasma cholesterol in HDL fraction, after 8 wks on atherogenic diet [%] |
| Cholesterol | Paigen1 | MPD:29 | totalCh | 5.9 | 3 | 3 | total cholesterol (plasma), after 8 wks on atherogenic diet [mg/dL] |
| Cholesterol | Paigen1 | MPD:29 | totHCC | 6.5 | 15 | 27 | hepatic cholesterol concentration, after 8 wks on atherogenic diet (total per g liver) [mg] |
| Cholesterol | Paigen2 | MPD:99 | CHOL | 6.5 | 4 | 4 | total cholesterol [mg/dL] |
| Cholesterol | Paigen2 | MPD:99 | CHOL_chg | 6.5 | 4 | 5 | fold change in total cholesterol after 17 wks on atherogenic diet [fold] |
| Cholesterol | Paigen2 | MPD:99 | CHOL_fat17 | 5.9 | 28 | 49 | total cholesterol, after 17 wks on atherogenic diet [mg/dL] |
| Cholesterol | Paigen2 | MPD:99 | HDLC | 6.9 | 2 | 4 | HDL cholesterol [mg/dL] |
| Cholesterol | Paigen2 | MPD:99 | HDLC_chg | 5.9 | 6 | 16 | fold change in HDL cholesterol after 17 wks on atherogenic diet [fold] |
| Cholesterol | Paigen2 | MPD:99 | HDLC_fat17 | 6.5 | 8 | 17 | HDL cholesterol, after 17 wks on atherogenic diet [mg/dL] |
| Cholesterol | Paigen2 | MPD:99 | nonHDL | 6.2 | 1 | 1 | non-HDL cholesterol [mg/dL] |
| Cholesterol | Paigen2 | MPD:99 | nonHDL_chg | 6.0 | 0 | 0 | fold change in non-HDL cholesterol after 17 wks on atherogenic diet [fold] |
| Cholesterol | Paigen2 | MPD:99 | nonHDL_fat17 | 6.0 | 38 | 109 | non-HDL cholesterol, after 17 wks on atherogenic diet [mg/dL] |
| Cholesterol | Paigen4 | MPD:197 | CHOL | 6.5 | 9 | 11 | total cholesterol [mg/dL] |
| Cholesterol | Paigen4 | MPD:197 | HDLC | 6.5 | 14 | 16 | HDL cholesterol [mg/dL] |
| Cholesterol | Paigen4 | MPD:197 | nonHDL | 6.7 | 1 | 1 | non-HDL cholesterol [mg/dL] |
| Drinking preference | Tordoff1 | MPD:61 | CaCl2_pref10 | 6.3 | 0 | 0 | preference for 10mM CaCl2 solution [%] |
| Drinking preference | Tordoff1 | MPD:61 | CaCl2_pref100 | 6.2 | 0 | 0 | preference for 100mM CaCl2 solution [%] |
| Drinking preference | Tordoff1 | MPD:61 | CaCl2_pref3 | 5.9 | 0 | 0 | preference for 3mM CaCl2 solution [%] |
| Drinking preference | Tordoff1 | MPD:61 | CaCl2_pref30 | 6.0 | 0 | 0 | preference for 30mM CaCl2 solution [%] |
| Drinking preference | Tordoff1 | MPD:61 | KCl_pref10 | 5.6 | 0 | 0 | preference for 10mM KCl solution [%] |
| Drinking preference | Tordoff1 | MPD:61 | KCl_pref100 | 5.6 | 0 | 0 | preference for 100mM KCl solution [%] |
| Drinking preference | Tordoff1 | MPD:61 | KCl_pref200 | 7.3 | 0 | 0 | preference for 200mM KCl solution [%] |
| Drinking preference | Tordoff1 | MPD:61 | KCl_pref30 | 6.5 | 0 | 0 | preference for 30mM KCl solution [%] |
| Drinking preference | Tordoff1 | MPD:61 | KCl_pref300 | 6.7 | 0 | 0 | preference for 300mM KCl solution [%] |
| Drinking preference | Tordoff1 | MPD:61 | NaCl_pref150 | 6.1 | 0 | 0 | preference for 150mM NaCl solution [%] |
| Drinking preference | Tordoff1 | MPD:61 | NaCl_pref300 | 6.7 | 0 | 0 | preference for 300mM NaCl solution [%] |
| Drinking preference | Tordoff1 | MPD:61 | NaCl_pref450 | 7.6 | 1 | 1 | preference for 450mM NaCl solution [%] |
| Drinking preference | Tordoff1 | MPD:61 | NaCl_pref75 | 6.0 | 0 | 0 | preference for 75mM NaCl solution [%] |
| Drinking preference | Tordoff1 | MPD:61 | NH4Cl_pref10 | 6.4 | 0 | 0 | preference for 10mM NH4Cl solution [%] |
| Drinking preference | Tordoff1 | MPD:61 | NH4Cl_pref100 | 5.5 | 0 | 0 | preference for 100mM NH4Cl solution [%] |
| Drinking preference | Tordoff1 | MPD:61 | NH4Cl_pref30 | 6.3 | 0 | 0 | preference for 30mM NH4Cl solution [%] |
| Drinking preference | Tordoff1 | MPD:61 | NH4Cl_pref300 | 7.7 | 0 | 0 | preference for 300mM NH4Cl solution [%] |
| Drinking preference | Tordoff3 | MPD:103 | CaCl2_pref25 | 7.6 | 6 | 8 | preference for 25mM CaCl2 solution [%] |
| Drinking preference | Tordoff3 | MPD:103 | CaCl2_pref7 | 6.9 | 23 | 108 | preference for 7.5mM CaCl2 solution [%] |
| Drinking preference | Tordoff3 | MPD:103 | CaCl2_pref75 | 7.4 | 1 | 1 | preference for 75mM CaCl2 solution [%] |
| Drinking preference | Tordoff3 | MPD:103 | CaLa_pref25 | 6.7 | 15 | 50 | preference for 25mM CaLa solution [%] |
| Drinking preference | Tordoff3 | MPD:103 | CaLa_pref7 | 7.6 | 14 | 61 | preference for 7.5mM CaLa solution [%] |
| Drinking preference | Tordoff3 | MPD:103 | CaLa_pref75 | 7.3 | 15 | 30 | preference for 75mM CaLa solution [%] |
| Drinking preference | Tordoff3 | MPD:103 | NaCl_pref225 | 7.1 | 25 | 69 | preference for 225mM NaCl solution [%] |
| Drinking preference | Tordoff3 | MPD:103 | NaCl_pref25 | 6.9 | 3 | 6 | preference for 25mM NaCl solution [%] |
| Drinking preference | Tordoff3 | MPD:103 | NaCl_pref75 | 6.9 | 12 | 44 | preference for 75mM NaCl solution [%] |
| Drinking preference | Tordoff3 | MPD:103 | NaLa_pref225 | 7.2 | 7 | 15 | preference for 225mM NaLa solution [%] |
| Drinking preference | Tordoff3 | MPD:103 | NaLa_pref25 | 6.6 | 3 | 6 | preference for 25mM NaLa solution [%] |
| Drinking preference | Tordoff3 | MPD:103 | NaLa_pref75 | 6.9 | 12 | 28 | preference for 75mM NaLa solution [%] |
| Gallbladder and gallstones | Paigen1 | MPD:29 | agLC | 6.2 | 0 | 0 | aggregated liquid crystals, after 8 wks on atherogenic diet (score: 0-4) [score] |
| Gallbladder and gallstones | Paigen1 | MPD:29 | ChMC | 5.7 | 1 | 1 | cholesterol monohydrate crystals, after 8 wks on atherogenic diet [score] |
| Gallbladder and gallstones | Paigen1 | MPD:29 | fusedLC | 6.1 | 1 | 2 | fused liquid crystals, after 8 wks on atherogenic diet (score: 0-4) [score] |
| Gallbladder and gallstones | Paigen1 | MPD:29 | gbvol | 6.1 | 19 | 49 | gallbladder volume, after 8 wks on atherogenic diet [uL] |
| Gallbladder and gallstones | Paigen1 | MPD:29 | gs | 8.1 | 0 | 0 | number of true gallstones, after 8 wks on atherogenic diet [n] |
| Gallbladder and gallstones | Paigen1 | MPD:29 | mucin | 6.2 | 2 | 6 | mucin, after 8 wks on atherogenic diet (score: 0-4) [score] |
| Gallbladder and gallstones | Paigen1 | MPD:29 | nstones | 8.3 | 0 | 0 | number of sandy plus true stones, after 8 wks on atherogenic diet [n] |
| Gallbladder and gallstones | Paigen1 | MPD:29 | sandysto | 9.4 | 0 | 0 | number of sandy stones, after 8 wks on atherogenic diet [n] |
| Gallbladder and gallstones | Paigen1 | MPD:29 | smallLC | 7.5 | 0 | 0 | small liquid crystals, after 8 wks on atherogenic diet (score: 0-4) [score] |
| Gallbladder and gallstones | Paigen1 | MPD:29 | st_pres | 7.3 | 0 | 0 | presence of stones, after 8 wks on atherogenic diet [score] |
| Hematology | Peters1 | MPD:62 | BASO | 5.9 | 0 | 0 | % basophils (percent of total number of leukocytes) [%] |
| Hematology | Peters1 | MPD:62 | CHCM | 5.9 | 1 | 2 | red cell hemoglobin concentration mean [g/dL] |
| Hematology | Peters1 | MPD:62 | cHGB | 6.0 | 2 | 2 | calculated hemoglobin (HGB) [g/dL] |
| Hematology | Peters1 | MPD:62 | EOS | 5.7 | 1 | 3 | % eosinophils (percent of total number of leukocytes) [%] |
| Hematology | Peters1 | MPD:62 | HCT | 6.0 | 1 | 1 | hematocrit (HCT) [%] |
| Hematology | Peters1 | MPD:62 | HDW | 6.7 | 0 | 0 | hemoglobin concentration distribution width [g/dL] |
| Hematology | Peters1 | MPD:62 | LUC | 6.0 | 11 | 26 | % large unstained cells (percent of total number of leukocytes) [%] |
| Hematology | Peters1 | MPD:62 | LYM | 6.6 | 17 | 60 | % lymphocytes (percent of total number of leukocytes) [%] |
| Hematology | Peters1 | MPD:62 | MCH | 6.5 | 0 | 0 | mean cell hemoglobin [pg] |
| Hematology | Peters1 | MPD:62 | MCHC | 5.9 | 0 | 0 | mean cell hemoglobin concentration [g/dL] |
| Hematology | Peters1 | MPD:62 | MCV | 6.6 | 18 | 44 | mean cell volume [fL] |
| Hematology | Peters1 | MPD:62 | mHGB | 5.9 | 8 | 14 | measured hemoglobin (HGB) [g/dL] |
| Hematology | Peters1 | MPD:62 | MONO | 6.5 | 15 | 30 | % monocytes (percent of total number of leukocytes) [%] |
| Hematology | Peters1 | MPD:62 | MPV | 6.6 | 0 | 0 | mean platelet volume [fL] |
| Hematology | Peters1 | MPD:62 | PLT | 6.9 | 1 | 2 | platelet count (units per volume x 103) [n/uL] |
| Hematology | Peters1 | MPD:62 | RBC | 6.0 | 21 | 57 | red blood cell count (RBC) (units per volume x 106) [n/uL] |
| Hematology | Peters1 | MPD:62 | RDW | 6.3 | 1 | 3 | red cell distribution width [%] |
| Hematology | Peters1 | MPD:62 | Retic | 5.8 | 2 | 2 | % reticulocytes (percent of total number of erythrocytes) [%] |
| Hematology | Peters1 | MPD:62 | WBC | 6.0 | 29 | 67 | white blood cell count (WBC) (units per volume x 103) [n/uL] |
| Liver pathology | Paigen1 | MPD:29 | ALT | 6.8 | 0 | 0 | alanine aminotransferase (plasma), after 8 wks on atherogenic diet [IU/L] |
| Liver pathology | Paigen1 | MPD:29 | bilesalts | 5.9 | 1 | 1 | bile salts (plasma), after 8 wks on atherogenic diet [umol/L] |
| Liver pathology | Paigen1 | MPD:29 | inflammation | 5.4 | 8 | 26 | inflammation, after 8 wks on atherogenic diet (score: 0-4) [score] |
| Liver pathology | Paigen1 | MPD:29 | liverweight | 6.8 | 10 | 83 | liver weight at sacrifice, after 8 wks on atherogenic diet [g] |
| Liver pathology | Paigen1 | MPD:29 | macrovacs | 7.9 | 2 | 2 | macrovacuoles, after 8 wks on atherogenic diet (score: 0-4) [score] |
| Liver pathology | Paigen1 | MPD:29 | microvacs | 5.4 | 1 | 1 | microvacuoles, after 8 wks on atherogenic diet (score: 0-4) [score] |
| Musculoskeletal | Naggert1 | MPD:143 | BMC_fat8 | 5.8 | 20 | 81 | bone mineral content (BMC) after 8 wks on atherogenic diet [g] |
| Musculoskeletal | Naggert1 | MPD:143 | BMD_fat8 | 6.0 | 28 | 116 | bone mineral density (BMD) after 8 wks on atherogenic diet [g/cm2] |
| Musculoskeletal | Tordoff3 | MPD:103 | BMC | 7.5 | 7 | 10 | bone mineral content [g] |
| Musculoskeletal | Tordoff3 | MPD:103 | BMD | 8.0 | 4 | 28 | bone mineral density [g/cm2] |
| Neurological | Johnson1 | MPD:14 | click_old | 5.5 | 5 | 7 | ABR threshold for click stimulus (SPL) [dB] |
| Neurological | Johnson1 | MPD:14 | click_yng | 6.5 | 0 | 0 | ABR threshold for click stimulus (SPL) [dB] |
| Neurological | Johnson1 | MPD:14 | pip16kHz_old | 5.5 | 11 | 22 | ABR threshold for 16kHz pip stimulus (SPL) [dB] |
| Neurological | Johnson1 | MPD:14 | pip16kHz_yng | 5.9 | 0 | 0 | ABR threshold for 16kHz pip stimulus (SPL) [dB] |
| Neurological | Johnson1 | MPD:14 | pip32kHz_old | 5.5 | 8 | 29 | ABR threshold for 32kHz pip stimulus (SPL) [dB] |
| Neurological | Johnson1 | MPD:14 | pip32kHz_yng | 6.3 | 0 | 0 | ABR threshold for 32kHz pip stimulus (SPL) [dB] |
| Neurological | Johnson1 | MPD:14 | pip8kHz_old | 6.3 | 4 | 10 | ABR threshold for 8kHz pip stimulus (SPL) [dB] |
| Neurological | Johnson1 | MPD:14 | pip8kHz_yng | 6.8 | 1 | 1 | ABR threshold for 8kHz pip stimulus (SPL) [dB] |
| Neurological | Willott1 | MPD:91 | ASR_100 | 6.0 | 21 | 40 | acoustic startle response evoked by 100 dB SPL [amplitude] |
| Neurological | Willott1 | MPD:91 | ASR_70 | 6.0 | 14 | 29 | acoustic startle response evoked by 70 dB SPL [amplitude] |
| Neurological | Willott1 | MPD:91 | ASR_80 | 6.7 | 5 | 12 | acoustic startle response evoked by 80 dB SPL [amplitude] |
| Neurological | Willott1 | MPD:91 | ASR_90 | 6.0 | 19 | 40 | acoustic startle response evoked by 90 dB SPL [amplitude] |
| Neurological | Willott1 | MPD:91 | ASR_habituation | 5.8 | 2 | 4 | ratio of final 4 trials to initial 4 trials (<1.0=diminished ASR) [ratio] |
| Neurological | Willott1 | MPD:91 | ASR_latency | 6.0 | 4 | 5 | mean latency of acoustic startle response evoked by 100 dB SPL [ms] |
| Neurological | Willott1 | MPD:91 | PPI_12 | 6.2 | 6 | 9 | mean prepulse inhibition, 70 dB at 12 kHz |
| Neurological | Willott1 | MPD:91 | PPI_20 | 6.7 | 5 | 11 | mean prepulse inhibition, 70 dB at 20 kHz |
| Neurological | Willott1 | MPD:91 | PPI_4 | 5.9 | 8 | 15 | mean prepulse inhibition, 70 dB at 4 kHz |
| Neurological | Willott1 | MPD:91 | PPI_tot | 6.2 | 10 | 16 | mean prepulse inhibition, 70 dB for all tones |
| Reproduction | Jax3 | MPD:149 | age_dam_lit1 | 5.9 | 4 | 8 | age of dam at birth of first litter [d] |
| Reproduction | Jax3 | MPD:149 | dams_pro | 7.0 | 0 | 0 | number of dams productive [n] |
| Reproduction | Jax3 | MPD:149 | littersborn | 6.7 | 0 | 0 | number of litters per dam [n] |
| Reproduction | Jax3 | MPD:149 | mating_to_retire | 4.3 | 0 | 0 | time from mating to retirement [wks] |
| Reproduction | Jax3 | MPD:149 | mice_per_litter | 7.0 | 0 | 0 | number of mice per litter [n] |
| Reproduction | Jax3 | MPD:149 | miceborn | 6.2 | 0 | 0 | number of mice born per dam [n] |
| Reproduction | Jax3 | MPD:149 | miceweaned | 6.5 | 0 | 0 | number of mice weaned per dam [n] |
| Reproduction | Jax3 | MPD:149 | nmatings | 6.7 | 0 | 0 | number of matings [n] |
| Reproduction | Jax3 | MPD:149 | pct_male_wean | 6.1 | 0 | 0 | percent males at weaning [%] |
| Reproduction | Jax3 | MPD:149 | pct_nonprod | 6.8 | 0 | 0 | percent matings that were nonproductive [%] |
| Reproduction | Jax3 | MPD:149 | pct_wean | 6.6 | 0 | 0 | percent weaned (of total born) [%] |
| Triglycerides | Paigen2 | MPD:99 | TG | 6.7 | 13 | 26 | triglycerides [mg/dL] |
| Triglycerides | Paigen2 | MPD:99 | TG_chg | 5.8 | 6 | 12 | fold change in triglycerides after 17 wks on atherogenic diet [fold] |
| Triglycerides | Paigen2 | MPD:99 | TG_fat17 | 6.0 | 0 | 0 | triglycerides, after 17 wks on atherogenic diet [mg/dL] |
| Triglycerides | Paigen4 | MPD:197 | TG | 6.7 | 37 | 76 | triglycerides [mg/dL] |
| Others | Paigen1 | MPD:29 | aortic_lesion | 5.7 | 0 | 0 | fatty streak aortic lesion size, after 8 wks on atherogenic diet [um2] |
| Others | Paigen2 | MPD:99 | aortic_lesion | 6.7 | 0 | 0 | fatty streak aortic lesion size after 18 wks on atherogenic diet [um2] |
| Others | Tordoff2 | MPD:63 | fiavg | 5.9 | 0 | 0 | daily average food intake [g] |
| Others | Tordoff2 | MPD:63 | fiavgadj | 6.1 | 0 | 0 | daily average food intake adjusted for body weight (30g) [g] |
| Others | Tordoff2 | MPD:63 | pctr | 7.0 | 0 | 0 | percent preference for right spout [%] |
| Others | Tordoff2 | MPD:63 | wiavg | 6.6 | 0 | 0 | daily average water intake [mL] |
| Others | Tordoff2 | MPD:63 | wiavgadj | 6.4 | 0 | 0 | daily average water intake adjusted for body weight (30g) [mL] |
