# Supplementary material for: Large-Scale In Silico Mapping of Complex Quantitative Traits in Inbred Mice
Source: PLoS One. 2007 Jul 25;2(7):e651. doi: 10.1371/journal.pone.0000651 (PMC1920557; doi:10.1371/journal.pone.0000651)
Supplement: Table S2 — Summary of 937 QTL identified in genome-wide association analyses. The table gives the chromosome, position of QTL peak (bp, based on the NCBI genome build 36), QTL interval, and -log (P) for each QTL identified in GWA scans. The table also provides the SNP ID of the most significant SNP and its dbSNP's annotation if available, and total number of significant and suggestive SNPs for each QTL. (1.99 MB DOC) [file pone.0000651.s003.doc]

**Table S2. Summary of 937 QTL identified in genome-wide** association analyses

| **Project** | **Trait** | **Ch** | **Pos** | **-log(P)** | **Left** | **Right** | **SigSNP** | **SugSNP** | **MostSNP** | **ncbi_ann** |
| --- | --- | --- | --- | --- | --- | --- | --- | --- | --- | --- |
| Hunter1 | ratio_mta | 1 | 177424998 | 6.3 | 177424998 | 177424998 | 1 | 0 | 1-175718769 | NA |
| Hunter1 | tum_burd | 4 | 135631310 | 6.4 | 135209220 | 136430582 | 1 | 29 | 4-134817249 | NA |
| Jax3 | age_dam_lit1 | 3 | 65207763 | 7.0 | 65207763 | 65333533 | 3 | 1 | 3-65357827 | Kcnab1:16497:intron:1:9841:156496 |
| Jax3 | age_dam_lit1 | 3 | 144292388 | 6.7 | 144078435 | 144536950 | 2 | 10 | 3-144921858 | NA |
| Jax3 | age_dam_lit1 | 8 | 12849161 | 5.9 | 11570067 | 13375216 | 1 | 14 | 8-12865515 | Atp11a:50770:intron:23:321:911 |
| Jax3 | age_dam_lit1 | 13 | 21689478 | 5.9 | 21689478 | 21725835 | 2 | 0 | 13-21062137 | NA |
| Johnson1 | click_old | 2 | 173193530 | 5.5 | 173193530 | 173193530 | 1 | 0 | 2-173614022 | NA |
| Johnson1 | click_old | 5 | 113120790 | 6.0 | 113120790 | 113120790 | 1 | 0 | 5-110229195 | Myo18b:74376:intron:2:7856:10115 |
| Johnson1 | click_old | 6 | 7222703 | 5.7 | 7222703 | 7559665 | 1 | 5 | 6-6996861 | NA |
| Johnson1 | click_old | 6 | 13135398 | 6.1 | 12660009 | 14484881 | 3 | 9 | 6-12941155 | NA |
| Johnson1 | click_old | 7 | 28595047 | 5.7 | 28595047 | 28595047 | 1 | 0 | 7-17855268 | Capn12:60594:intron:5:390:473 |
| Johnson1 | pip16kHz_old | 1 | 181179443 | 5.6 | 181179443 | 181679704 | 1 | 4 | 1-179365217 | Smyd3:69726:intron:5:62513:310878 |
| Johnson1 | pip16kHz_old | 1 | 192635950 | 5.6 | 192421897 | 194315817 | 5 | 3 | 1-190823854 | Angel2:52477:intron:2:2142:4249 |
| Johnson1 | pip16kHz_old | 2 | 173193530 | 5.8 | 173193530 | 173193530 | 1 | 0 | 2-173614022 | NA |
| Johnson1 | pip16kHz_old | 5 | 113120790 | 6.0 | 113120790 | 113120790 | 1 | 0 | 5-110229195 | Myo18b:74376:intron:2:7856:10115 |
| Johnson1 | pip16kHz_old | 5 | 128894089 | 5.6 | 128894089 | 128894089 | 1 | 0 | 5-126134240 | NA |
| Johnson1 | pip16kHz_old | 6 | 7222703 | 6.5 | 7222703 | 7559665 | 5 | 4 | 6-6996861 | NA |
| Johnson1 | pip16kHz_old | 6 | 13135398 | 6.9 | 12660009 | 14445990 | 4 | 7 | 6-12941155 | NA |
| Johnson1 | pip16kHz_old | 6 | 27696207 | 5.6 | 27696207 | 27696207 | 1 | 0 | 6-27593861 | Grm8:14823:intron:5:13976:141486 |
| Johnson1 | pip16kHz_old | 10 | 91117672 | 5.6 | 91117672 | 91117796 | 1 | 3 | 10-91455667 | NA |
| Johnson1 | pip16kHz_old | 15 | 25953334 | 5.6 | 25953334 | 25953478 | 1 | 1 | 15-26050295 | NA |
| Johnson1 | pip16kHz_old | 18 | 19310200 | 5.6 | 19310200 | 19310200 | 1 | 0 | 18-19548661 | NA |
| Johnson1 | pip32kHz_old | 2 | 104502169 | 5.6 | 104502169 | 104502169 | 1 | 0 | rs3674721 | Tcp11l1:320554:intron:1:5024:6379 |
| Johnson1 | pip32kHz_old | 2 | 173193530 | 5.6 | 173193530 | 173193530 | 1 | 0 | 2-173614022 | NA |
| Johnson1 | pip32kHz_old | 5 | 113120790 | 6.0 | 113120790 | 113120790 | 1 | 0 | 5-110229195 | Myo18b:74376:intron:2:7856:10115 |
| Johnson1 | pip32kHz_old | 6 | 7222703 | 6.1 | 7222703 | 7559665 | 5 | 4 | 6-6996861 | NA |
| Johnson1 | pip32kHz_old | 6 | 13135398 | 6.4 | 12660009 | 14484881 | 9 | 4 | 6-12941155 | NA |
| Johnson1 | pip32kHz_old | 7 | 28595047 | 5.7 | 28595047 | 28595047 | 1 | 0 | 7-17855268 | Capn12:60594:intron:5:390:473 |
| Johnson1 | pip32kHz_old | 10 | 104673784 | 5.6 | 104673784 | 104673784 | 1 | 0 | rs3699498 | Tmtc2:278279:intron:8:1823:16588 |
| Johnson1 | pip32kHz_old | 18 | 28381596 | 5.5 | 28381596 | 29054723 | 10 | 3 | 18-28677476 | NA |
| Johnson1 | pip8kHz_old | 2 | 104502169 | 6.4 | 104502169 | 104502169 | 1 | 0 | rs3674721 | Tcp11l1:320554:intron:1:5024:6379 |
| Johnson1 | pip8kHz_old | 6 | 13135398 | 6.6 | 13135398 | 14484881 | 7 | 4 | 6-12941155 | NA |
| Johnson1 | pip8kHz_old | 10 | 104673784 | 6.4 | 104673784 | 104673784 | 1 | 0 | rs3699498 | Tmtc2:278279:intron:8:1823:16588 |
| Johnson1 | pip8kHz_old | 18 | 28424653 | 7.4 | 28381596 | 29054723 | 1 | 11 | 18-28722028 | NA |
| Johnson1 | pip8kHz_yng | 13 | 104381991 | 7.2 | 104381991 | 104617882 | 1 | 3 | rs3705092 | Mast4:328329:intron:1:70939:160991 |
| Naggert1 | BMC_fat8 | 1 | 13241109 | 7.0 | 12783173 | 13241109 | 1 | 4 | 1-13356759 | Ncoa2:17978:intron:2:30072:61253 |
| Naggert1 | BMC_fat8 | 1 | 19624120 | 7.2 | 19458611 | 19865238 | 16 | 6 | 1-19845359 | NA |
| Naggert1 | BMC_fat8 | 1 | 154392258 | 6.4 | 154234171 | 154997648 | 3 | 1 | 1-152598184 | NA |
| Naggert1 | BMC_fat8 | 1 | 159877223 | 7.2 | 158818134 | 160006196 | 2 | 9 | 1-158043625 | NA |
| Naggert1 | BMC_fat8 | 3 | 52829972 | 8.3 | 52816831 | 53503132 | 8 | 7 | 3-52827373 | NA |
| Naggert1 | BMC_fat8 | 3 | 89649617 | 6.1 | 89649617 | 89719595 | 1 | 2 | 3-89988691 | Kcnn3:140493:intron:1:42284:43452 |
| Naggert1 | BMC_fat8 | 5 | 106405797 | 7.3 | 106405797 | 106405797 | 1 | 0 | 5-103564644 | NA |
| Naggert1 | BMC_fat8 | 7 | 67373720 | 6.6 | 67322670 | 67565349 | 2 | 4 | 7-54531509 | NA |
| Naggert1 | BMC_fat8 | 8 | 71127361 | 6.1 | 71127361 | 71127361 | 1 | 0 | 8-67224185 | NA |
| Naggert1 | BMC_fat8 | 11 | 56872797 | 5.9 | 56800722 | 57006349 | 1 | 6 | 11-56669117 | NA |
| Naggert1 | BMC_fat8 | 12 | 15581276 | 6.6 | 15537662 | 15720179 | 2 | 3 | 12-15680096 | NA |
| Naggert1 | BMC_fat8 | 12 | 34720698 | 5.9 | 34268142 | 34720698 | 1 | 3 | 12-29156844 | NA |
| Naggert1 | BMC_fat8 | 12 | 54757646 | 6.1 | 54757646 | 55221130 | 1 | 2 | rs4135641 | Npas3:27386:intron:5:4233:115375 |
| Naggert1 | BMC_fat8 | 12 | 102592379 | 6.2 | 102306509 | 102594866 | 1 | 2 | 12-96871053 | Slc24a4:238384:intron:2:59707:82239 |
| Naggert1 | BMC_fat8 | 13 | 9647467 | 7.3 | 7027401 | 11534269 | 31 | 31 | 13-9529316 | Dip2c:208440:intron:35:1180:7561 |
| Naggert1 | BMC_fat8 | 14 | 77617802 | 6.4 | 77155749 | 77677543 | 1 | 6 | 14-70656527 | 1300010F03Rik:219189:intron:3:14790:39265 |
| Naggert1 | BMC_fat8 | 15 | 41390293 | 6.1 | 41248908 | 41390293 | 1 | 1 | 15-41534353 | NA |
| Naggert1 | BMC_fat8 | 15 | 82173783 | 6.9 | 82046556 | 82802497 | 5 | 5 | rs13482674 | NA |
| Naggert1 | BMC_fat8 | 16 | 77122426 | 6.2 | 76984585 | 77452383 | 1 | 12 | rs4207150 | NA |
| Naggert1 | BMC_fat8 | 19 | 43629246 | 5.9 | 43629246 | 43629246 | 1 | 0 | 19-42912593 | NA |
| Naggert1 | BMD_fat8 | 1 | 10018635 | 6.1 | 10018635 | 10266857 | 1 | 3 | 1-10102531 | Cops5:26754:intron:3:585:682 |
| Naggert1 | BMD_fat8 | 1 | 13241109 | 9.7 | 12783173 | 13554219 | 2 | 8 | 1-13356759 | Ncoa2:17978:intron:2:30072:61253 |
| Naggert1 | BMD_fat8 | 1 | 19458943 | 7.4 | 19458611 | 21016033 | 21 | 7 | 1-19680103 | NA |
| Naggert1 | BMD_fat8 | 1 | 30730302 | 7.0 | 30496807 | 31168310 | 1 | 12 | 1-31057361 | NA |
| Naggert1 | BMD_fat8 | 1 | 77470824 | 7.0 | 77470824 | 78020515 | 1 | 5 | 1-78041665 | NA |
| Naggert1 | BMD_fat8 | 1 | 92413623 | 6.8 | 92234401 | 93480953 | 2 | 18 | 1-90492455 | NA |
| Naggert1 | BMD_fat8 | 1 | 117755780 | 6.3 | 117663936 | 121001001 | 13 | 16 | 1-115948804 | Caspr5-1:636808:intron:3:12897:144786 |
| Naggert1 | BMD_fat8 | 1 | 171087801 | 6.1 | 170936401 | 171087801 | 1 | 1 | 1-169327052 | NA |
| Naggert1 | BMD_fat8 | 3 | 52829972 | 8.0 | 52816831 | 53105788 | 6 | 8 | 3-52827373 | NA |
| Naggert1 | BMD_fat8 | 4 | 59091383 | 6.2 | 59091383 | 59538592 | 8 | 7 | 4-58265428 | NA |
| Naggert1 | BMD_fat8 | 5 | 45053820 | 6.4 | 44734287 | 45576429 | 22 | 11 | 5-43379390 | Ldb2:16826:intron:1:33789:129901 |
| Naggert1 | BMD_fat8 | 5 | 92825965 | 7.0 | 92825965 | 93186118 | 1 | 7 | rs13478392 | NA |
| Naggert1 | BMD_fat8 | 5 | 106405797 | 8.8 | 106405797 | 108224621 | 4 | 6 | 5-103564644 | NA |
| Naggert1 | BMD_fat8 | 7 | 68814687 | 6.2 | 68814687 | 68814687 | 1 | 0 | 7-55974622 | NA |
| Naggert1 | BMD_fat8 | 9 | 34706768 | 6.7 | 33955531 | 34711139 | 2 | 3 | 9-34936615 | Kirrel3:67703:intron:2:12576:46414 |
| Naggert1 | BMD_fat8 | 10 | 113923530 | 6.4 | 113821844 | 113994828 | 5 | 16 | 10-114295245 | Trhde:237553:intron:6:47705:48928 |
| Naggert1 | BMD_fat8 | 11 | 56872797 | 7.3 | 56800722 | 57006349 | 1 | 7 | 11-56669117 | NA |
| Naggert1 | BMD_fat8 | 12 | 34720698 | 7.3 | 34268142 | 34720698 | 3 | 1 | 12-29156844 | NA |
| Naggert1 | BMD_fat8 | 12 | 102592379 | 7.8 | 101510449 | 102594866 | 3 | 4 | 12-96871053 | Slc24a4:238384:intron:2:59707:82239 |
| Naggert1 | BMD_fat8 | 14 | 77617802 | 6.3 | 77155749 | 77858149 | 1 | 14 | 14-70656527 | 1300010F03Rik:219189:intron:3:14790:39265 |
| Naggert1 | BMD_fat8 | 15 | 9415902 | 7.1 | 9394428 | 9515201 | 2 | 1 | 15-9367469 | NA |
| Naggert1 | BMD_fat8 | 15 | 61130502 | 6.4 | 61130502 | 61130502 | 1 | 0 | 15-61503642 | NA |
| Naggert1 | BMD_fat8 | 16 | 35676508 | 6.1 | 35676508 | 35746382 | 1 | 1 | 16-35596874 | Dirc2:224132:intron:1:12136:18308 |
| Naggert1 | BMD_fat8 | 16 | 77122426 | 6.0 | 77122426 | 77240157 | 1 | 5 | rs4207150 | NA |
| Naggert1 | BMD_fat8 | 17 | 25922487 | 6.4 | 25794280 | 26204203 | 1 | 3 | 17-24780588 | A20in1:12005:intron:10:779:826 |
| Naggert1 | BMD_fat8 | 17 | 45745080 | 6.8 | 45745080 | 45745080 | 1 | 0 | 17-44438087 | Tjap1:74094:intron:2:845:10415 |
| Naggert1 | BMD_fat8 | 17 | 78350303 | 6.8 | 78299516 | 78350303 | 2 | 0 | 17-76988591 | NA |
| Naggert1 | BMD_fat8 | 19 | 56364485 | 7.6 | 56243373 | 56364527 | 8 | 0 | 19-55696244 | Habp2:226243:intron:5:12:447 |
| Naggert1 | bw_fat8 | 1 | 77807006 | 7.8 | 77470824 | 78020515 | 1 | 4 | 1-78378141 | NA |
| Naggert1 | bw_fat8 | 1 | 154888397 | 9.3 | 153497361 | 154997648 | 4 | 3 | 1-153100355 | Lamc2:16782:intron:22:635:2530 |
| Naggert1 | bw_fat8 | 2 | 99954544 | 6.9 | 99892251 | 99954544 | 1 | 1 | 2-100047689 | NA |
| Naggert1 | bw_fat8 | 3 | 52829972 | 7.4 | 52829972 | 53105788 | 6 | 0 | 3-52827373 | NA |
| Naggert1 | bw_fat8 | 3 | 148361920 | 7.2 | 148361920 | 148361920 | 1 | 0 | 3-149001866 | NA |
| Naggert1 | bw_fat8 | 9 | 33977178 | 7.0 | 33766649 | 34012343 | 1 | 6 | 9-34205201 | NA |
| Naggert1 | bw_fat8 | 13 | 10065799 | 6.7 | 8305114 | 10065799 | 2 | 18 | 13-9950895 | Chrm3:12671:intron:3:55673:93726 |
| Naggert1 | bw_fat8 | 13 | 117625910 | 6.9 | 117625910 | 117625910 | 1 | 0 | 13-113467189 | NA |
| Naggert1 | fatwt_fat8 | 1 | 13180027 | 6.7 | 13180005 | 13180027 | 1 | 1 | 1-13295677 | Ncoa2:17978:intron:4:26492:31114 |
| Naggert1 | fatwt_fat8 | 1 | 78020515 | 8.3 | 77470824 | 78020515 | 1 | 3 | 1-78591854 | Pa203:18505:intron:4:55072:60006 |
| Naggert1 | fatwt_fat8 | 3 | 83313131 | 6.6 | 83069188 | 83313131 | 1 | 1 | 3-83623301 | NA |
| Naggert1 | fatwt_fat8 | 4 | 117562749 | 6.4 | 117553014 | 117567590 | 2 | 1 | 4-116410458 | St3gal3:20441:intron:2:42731:52850 |
| Naggert1 | fatwt_fat8 | 5 | 89689019 | 6.9 | 89405591 | 89883289 | 1 | 2 | 5-87392377 | Rufy3:52822:intron:1:30140:30622 |
| Naggert1 | fatwt_fat8 | 5 | 138615546 | 6.5 | 138238617 | 138965375 | 3 | 4 | 5-135765184 | NA |
| Naggert1 | fatwt_fat8 | 6 | 16932017 | 6.5 | 16793554 | 17115919 | 1 | 3 | rs30127354 | NA |
| Naggert1 | fatwt_fat8 | 7 | 16033062 | 7.5 | 15353504 | 16306027 | 12 | 11 | 7-8560374 | NA |
| Naggert1 | fatwt_fat8 | 7 | 85449579 | 7.0 | 82577292 | 86539760 | 4 | 36 | 7-72719884 | LOC620835:620835:intron:1:2064:9331 |
| Naggert1 | fatwt_fat8 | 7 | 107190010 | 7.1 | 106744047 | 107435911 | 13 | 3 | rs3724540 | Syt9:60510:intron:1:27685:53898 |
| Naggert1 | fatwt_fat8 | 12 | 60541374 | 7.4 | 60399708 | 60837201 | 1 | 6 | 12-54821627 | NA |
| Naggert1 | fatwt_fat8 | 15 | 84634030 | 6.5 | 84634030 | 84657099 | 1 | 1 | 15-85161872 | Phf21b:271305:intron:3:1801:2953 |
| Naggert1 | fatwt_fat8 | 16 | 52299815 | 6.8 | 52299815 | 52299815 | 1 | 0 | 16-52358882 | Alcam:11658:intron:1:71925:142479 |
| Naggert1 | fatwt_fat8 | 16 | 77143369 | 7.6 | 76225195 | 77227116 | 1 | 10 | 16-77620139 | NA |
| Naggert1 | fatwt_fat8 | 17 | 63187681 | 6.7 | 62847944 | 63187681 | 1 | 2 | 17-61850057 | NA |
| Naggert1 | fatwt_fat8 | 18 | 65681504 | 9.1 | 65654706 | 66284189 | 4 | 11 | 18-66085861 | NA |
| Naggert1 | fatwt_fat8 | 18 | 73638577 | 6.5 | 73638577 | 73638577 | 1 | 0 | 18-74039828 | NA |
| Naggert1 | GLU_fat17 | 1 | 162275320 | 7.7 | 162275320 | 162275320 | 1 | 0 | 1-160447771 | Rabgap1l:29809:intron:4:3179:49598 |
| Naggert1 | GLU_fat17 | 2 | 92768687 | 7.2 | 92768687 | 92768687 | 1 | 0 | 2-92861841 | NA |
| Naggert1 | GLU_fat17 | 2 | 142347536 | 6.4 | 142347536 | 142347536 | 1 | 0 | 2-142405118 | Kif16b:16558:intron:24:16192:23517 |
| Naggert1 | GLU_fat17 | 2 | 152792694 | 8.1 | 152211378 | 153119457 | 4 | 1 | 2-153164602 | NA |
| Naggert1 | GLU_fat17 | 4 | 122456877 | 8.3 | 122456877 | 122456989 | 2 | 0 | 4-121324889 | Mfsd2:76574:intron:3:2157:4601 |
| Naggert1 | GLU_fat17 | 4 | 124830567 | 6.9 | 124830567 | 124830567 | 1 | 0 | 4-123698579 | NA |
| Naggert1 | GLU_fat17 | 5 | 129556806 | 7.6 | 129556806 | 129556806 | 1 | 0 | 5-126801151 | NA |
| Naggert1 | GLU_fat17 | 8 | 70562472 | 6.4 | 70475165 | 70817423 | 1 | 8 | 8-66613040 | NA |
| Naggert1 | GLU_fat17 | 12 | 8397764 | 8.5 | 8374002 | 8520756 | 4 | 3 | 12-8338238 | NA |
| Naggert1 | GLU_fat17 | 13 | 86883500 | 7.4 | 86883500 | 86884670 | 2 | 0 | 13-83061547 | NA |
| Naggert1 | GLU_fat17 | 13 | 90268698 | 7.8 | 89560757 | 90437440 | 24 | 11 | 13-86452280 | NA |
| Naggert1 | GLU_fat17 | 15 | 47708522 | 6.9 | 47661546 | 48113097 | 1 | 9 | 15-47900025 | Csmd3:239420:intron:28:2896:23362 |
| Naggert1 | GLU_fat17 | 20 | 128725590 | 7.5 | 128725590 | 128725590 | 1 | 0 | 20-123386388 | NA |
| Naggert1 | leanwt_fat8 | 1 | 77807006 | 6.7 | 77807006 | 77807006 | 1 | 0 | 1-78378141 | NA |
| Naggert1 | leanwt_fat8 | 1 | 153497361 | 8.2 | 153497361 | 154888397 | 3 | 0 | 1-151699108 | NA |
| Naggert1 | leanwt_fat8 | 4 | 135740473 | 7.1 | 135209220 | 135740473 | 2 | 0 | 4-134926412 | NA |
| Naggert1 | leanwt_fat8 | 7 | 123693485 | 7.2 | 123693485 | 123693485 | 1 | 0 | 7-111375972 | NA |
| Naggert1 | LEP_fat18 | 1 | 34184913 | 6.2 | 33855115 | 34184913 | 1 | 5 | rs6282096 | Dst:13518:intron:60:11120:13371 |
| Naggert1 | LEP_fat18 | 1 | 148100184 | 7.7 | 147932015 | 148415184 | 1 | 16 | 1-146244337 | NA |
| Naggert1 | LEP_fat18 | 1 | 151655683 | 7.2 | 151643104 | 151655710 | 2 | 1 | 1-149823826 | Pla2g4a:18783:intron:5:7634:13453 |
| Naggert1 | LEP_fat18 | 1 | 190454669 | 8.8 | 186973251 | 190542884 | 37 | 26 | 1-188422580 | Ush2a:22283:intron:44:158:1341 |
| Naggert1 | LEP_fat18 | 2 | 144792586 | 7.5 | 144159522 | 145517504 | 4 | 55 | 2-144853124 | NA |
| Naggert1 | LEP_fat18 | 4 | 51265008 | 7.3 | 50703895 | 51336714 | 5 | 7 | 4-50488953 | NA |
| Naggert1 | LEP_fat18 | 4 | 56443443 | 5.9 | 55995138 | 56848629 | 3 | 20 | 4-55617488 | NA |
| Naggert1 | LEP_fat18 | 4 | 134753036 | 6.5 | 134014717 | 135351167 | 4 | 6 | 4-133938975 | Npal3:74552:intron:3:1510:2762 |
| Naggert1 | LEP_fat18 | 4 | 144622277 | 5.9 | 144595480 | 144622277 | 1 | 3 | 4-143690875 | NA |
| Naggert1 | LEP_fat18 | 5 | 87077989 | 6.6 | 87077989 | 87077989 | 1 | 0 | 5-84796273 | NA |
| Naggert1 | LEP_fat18 | 5 | 110822082 | 5.9 | 110822082 | 110945537 | 1 | 1 | rs3708939 | NA |
| Naggert1 | LEP_fat18 | 5 | 143183336 | 7.7 | 142899735 | 143283031 | 6 | 2 | 5-140398927 | NA |
| Naggert1 | LEP_fat18 | 6 | 44010215 | 8.5 | 43945311 | 45023477 | 33 | 10 | 6-44032535 | NA |
| Naggert1 | LEP_fat18 | 6 | 53681266 | 9.2 | 53256704 | 53681266 | 3 | 7 | 6-53702681 | NA |
| Naggert1 | LEP_fat18 | 6 | 55858855 | 6.3 | 55455674 | 57765934 | 16 | 119 | 6-55893413 | D530004J12Rik:232016:intron:9:27780:65561 |
| Naggert1 | LEP_fat18 | 7 | 17722562 | 5.9 | 17325265 | 17722562 | 1 | 2 | 7-10157042 | LOC545925:545925:intron:2:933:2875 |
| Naggert1 | LEP_fat18 | 7 | 86190109 | 6.6 | 86145613 | 86190109 | 1 | 1 | 7-73475624 | NA |
| Naggert1 | LEP_fat18 | 7 | 90211416 | 6.5 | 90211416 | 90211416 | 1 | 0 | 7-77568790 | NA |
| Naggert1 | LEP_fat18 | 7 | 107114264 | 7.6 | 106390700 | 107435911 | 17 | 13 | 7-94589695 | NA |
| Naggert1 | LEP_fat18 | 8 | 130622889 | 6.8 | 130589412 | 130995015 | 6 | 13 | 8-127143161 | NA |
| Naggert1 | LEP_fat18 | 9 | 38578979 | 6.1 | 38578979 | 38578979 | 1 | 0 | rs13480148 | Olfr923:258812:exon:1:715:953 |
| Naggert1 | LEP_fat18 | 9 | 107873847 | 9.6 | 107579895 | 108032270 | 14 | 3 | 9-108037887 | Ihpk1:27399:intron:1:12923:21278 |
| Naggert1 | LEP_fat18 | 9 | 111703646 | 6.0 | 111703646 | 111703646 | 1 | 0 | rs13480421 | NA |
| Naggert1 | LEP_fat18 | 11 | 96839275 | 7.6 | 96839275 | 96839275 | 1 | 0 | 11-96694308 | Sp6:83395:UTR:2:590:2140 |
| Naggert1 | LEP_fat18 | 12 | 105272488 | 7.3 | 105253528 | 105272488 | 1 | 5 | 12-99583964 | NA |
| Naggert1 | LEP_fat18 | 13 | 91342192 | 7.0 | 90959042 | 91422639 | 7 | 2 | 13-87540375 | NA |
| Naggert1 | LEP_fat18 | 13 | 117911580 | 6.2 | 117565107 | 118500852 | 1 | 4 | 13-113752909 | NA |
| Naggert1 | LEP_fat18 | 14 | 32376554 | 6.3 | 31187853 | 32461556 | 1 | 18 | 14-29590073 | NA |
| Naggert1 | LEP_fat18 | 14 | 48522720 | 7.9 | 48496711 | 49515246 | 4 | 18 | 14-42277483 | NA |
| Naggert1 | LEP_fat18 | 15 | 4324784 | 6.4 | 4043188 | 4324784 | 1 | 8 | 15-4198298 | NA |
| Naggert1 | LEP_fat18 | 15 | 51337145 | 7.3 | 51075707 | 51537463 | 2 | 17 | 15-51528429 | NA |
| Naggert1 | LEP_fat18 | 15 | 95520195 | 6.4 | 93614565 | 95820223 | 1 | 47 | 15-96119647 | NA |
| Naggert1 | LEP_fat18 | 16 | 27504687 | 6.3 | 27466924 | 27888931 | 5 | 7 | 16-27430509 | NA |
| Naggert1 | LEP_fat18 | 16 | 72353476 | 6.3 | 72339471 | 72353476 | 1 | 1 | 16-72820050 | NA |
| Naggert1 | LEP_fat18 | 16 | 89430052 | 7.3 | 88292143 | 90682966 | 15 | 15 | 16-89971269 | NA |
| Naggert1 | LEP_fat18 | 19 | 56282619 | 6.1 | 56243373 | 56364527 | 6 | 7 | 19-55613014 | NA |
| Naggert1 | pctfat_fat8 | 1 | 34184913 | 8.4 | 33855115 | 34257395 | 5 | 5 | rs6282096 | Dst:13518:intron:60:11120:13371 |
| Naggert1 | pctfat_fat8 | 1 | 78020515 | 10.2 | 77408912 | 78020515 | 1 | 7 | 1-78591854 | Pa203:18505:intron:4:55072:60006 |
| Naggert1 | pctfat_fat8 | 1 | 127875052 | 6.1 | 127482309 | 128473432 | 2 | 52 | 1-125878802 | E030049G20Rik:210356:intron:8:55036:68577 |
| Naggert1 | pctfat_fat8 | 1 | 187937839 | 6.7 | 187157724 | 188595578 | 5 | 10 | rs13476288 | NA |
| Naggert1 | pctfat_fat8 | 4 | 3715453 | 6.3 | 3715453 | 3715453 | 1 | 0 | 4-3715453 | Lyn:17096:intron:11:5200:6636 |
| Naggert1 | pctfat_fat8 | 4 | 51311986 | 6.4 | 51219943 | 51336714 | 1 | 10 | 4-50535931 | NA |
| Naggert1 | pctfat_fat8 | 4 | 120389337 | 6.6 | 120301256 | 120428336 | 3 | 3 | 4-119237178 | NA |
| Naggert1 | pctfat_fat8 | 4 | 123803756 | 6.1 | 123803756 | 123803756 | 1 | 0 | 4-122671768 | NA |
| Naggert1 | pctfat_fat8 | 4 | 150052076 | 6.6 | 148359338 | 150056885 | 3 | 3 | 4-149094218 | Camta1:100072:intron:6:81755:126060 |
| Naggert1 | pctfat_fat8 | 5 | 12518756 | 6.3 | 12231581 | 12667344 | 1 | 6 | 5-11558774 | Sema3d:108151:intron:7:4529:16029 |
| Naggert1 | pctfat_fat8 | 5 | 40964302 | 6.3 | 40954530 | 41042306 | 1 | 6 | rs13478204 | NA |
| Naggert1 | pctfat_fat8 | 6 | 16932017 | 6.1 | 16793554 | 17115919 | 1 | 3 | rs30127354 | NA |
| Naggert1 | pctfat_fat8 | 6 | 53280862 | 8.1 | 53157471 | 53883270 | 18 | 5 | 6-53302277 | NA |
| Naggert1 | pctfat_fat8 | 7 | 17722562 | 7.1 | 16862954 | 17722562 | 1 | 6 | 7-10157042 | LOC545925:545925:intron:2:933:2875 |
| Naggert1 | pctfat_fat8 | 7 | 98788799 | 6.1 | 98788799 | 98863244 | 1 | 1 | rs6405344 | Uvrag:78610:intron:13:26283:33306 |
| Naggert1 | pctfat_fat8 | 7 | 106604859 | 7.1 | 105510122 | 107126552 | 23 | 15 | 7-94078602 | NA |
| Naggert1 | pctfat_fat8 | 7 | 133991842 | 6.5 | 133984508 | 134009999 | 5 | 1 | 7-121778040 | Adam12:11489:intron:2:19282:37989 |
| Naggert1 | pctfat_fat8 | 9 | 6455720 | 7.6 | 6180572 | 8972389 | 2 | 17 | 9-6388205 | NA |
| Naggert1 | pctfat_fat8 | 9 | 33257134 | 6.9 | 32651251 | 33257134 | 1 | 5 | 9-33486487 | NA |
| Naggert1 | pctfat_fat8 | 10 | 14026419 | 6.9 | 13787281 | 14026419 | 2 | 2 | 10-14050358 | NA |
| Naggert1 | pctfat_fat8 | 11 | 65006545 | 6.2 | 64827488 | 65366826 | 1 | 5 | 11-64802898 | Myocd:214384:intron:9:4716:7250 |
| Naggert1 | pctfat_fat8 | 11 | 83260067 | 6.2 | 83180629 | 83265612 | 1 | 7 | 11-83045534 | Mmp28:118453:e20on:7:24:137 |
| Naggert1 | pctfat_fat8 | 11 | 93020755 | 6.9 | 92981051 | 93690652 | 5 | 27 | 11-92875788 | Car10:72605:intron:3:19507:119346 |
| Naggert1 | pctfat_fat8 | 12 | 40301722 | 6.0 | 40294395 | 40332860 | 2 | 3 | 12-34809507 | NA |
| Naggert1 | pctfat_fat8 | 12 | 51768770 | 6.3 | 51556325 | 51780111 | 1 | 4 | 12-46050248 | NA |
| Naggert1 | pctfat_fat8 | 15 | 98434034 | 6.1 | 98393515 | 98853132 | 1 | 3 | 15-99067916 | Adcy6:11512:intron:1:1480:2747 |
| Naggert1 | pctfat_fat8 | 16 | 72192644 | 6.5 | 72192644 | 72192644 | 1 | 0 | 16-72659168 | NA |
| Naggert1 | pctfat_fat8 | 16 | 77143369 | 7.4 | 77143369 | 77143369 | 1 | 0 | 16-77620139 | NA |
| Naggert1 | pctfat_fat8 | 17 | 25910596 | 7.4 | 25359864 | 27806071 | 17 | 40 | 17-24768697 | A20in1:12005:intron:4:409:1588 |
| Naggert1 | pctfat_fat8 | 17 | 49751315 | 7.4 | 49381104 | 49751315 | 1 | 4 | 17-48464878 | Dazl:13164:intron:7:501:1202 |
| Naggert1 | pctfat_fat8 | 17 | 72818562 | 7.3 | 72029266 | 73338407 | 21 | 12 | rs4137129 | NA |
| Naggert1 | tissuemass_fat8 | 1 | 77807006 | 7.3 | 77470824 | 78020515 | 2 | 4 | 1-78378141 | NA |
| Naggert1 | tissuemass_fat8 | 1 | 154888397 | 9.7 | 154392258 | 154997648 | 3 | 5 | 1-153100355 | Lamc2:16782:intron:22:635:2530 |
| Naggert1 | tissuemass_fat8 | 3 | 52959233 | 7.5 | 52816831 | 53105788 | 6 | 6 | 3-52955992 | NA |
| Paigen1 | bilesalts | 11 | 7006701 | 6.4 | 6980681 | 7006701 | 1 | 1 | 11-7001491 | Adcy1:432530:intron:3:6009:8310 |
| Paigen1 | bw_chg | 3 | 156508735 | 6.1 | 156345150 | 156671152 | 1 | 3 | 3-157188987 | Negr1:320840:intron:1:11089:297083 |
| Paigen1 | ChMC | 5 | 101468061 | 5.9 | 101468061 | 101482426 | 1 | 1 | 5-98568210 | NA |
| Paigen1 | estHCC | 1 | 3095984 | 8.4 | 3095984 | 3095984 | 1 | 0 | 1-3109918 | NA |
| Paigen1 | estHCC | 1 | 65478253 | 7.3 | 65478253 | 65478253 | 1 | 0 | 1-65942557 | NA |
| Paigen1 | estHCC | 1 | 122926908 | 7.3 | 122918903 | 123429919 | 2 | 5 | 1-120877598 | AI848258:98452:intron:3:273:20768 |
| Paigen1 | estHCC | 8 | 114944590 | 7.9 | 114944527 | 116029070 | 2 | 5 | 8-111399139 | NA |
| Paigen1 | estHCC | 10 | 17632967 | 7.1 | 17313283 | 17705142 | 2 | 3 | rs3712394 | Heca:380629:intron:1:3957:31474 |
| Paigen1 | estHCC | 12 | 104441364 | 7.4 | 104441364 | 104441364 | 1 | 0 | 12-98749306 | Serpina12:68054:intron:1:3947:5873 |
| Paigen1 | estHCC | 13 | 45370345 | 7.4 | 45349450 | 47687152 | 4 | 7 | 13-44823519 | NA |
| Paigen1 | estHCC | 18 | 77739967 | 6.9 | 77739967 | 77739967 | 1 | 0 | 18-77956214 | Gm96:225743:intron:2:29523:80576 |
| Paigen1 | finalbw | 1 | 153497361 | 9.4 | 153497361 | 154997648 | 5 | 8 | 1-151699108 | NA |
| Paigen1 | finalbw | 1 | 159877223 | 7.2 | 159877223 | 159877223 | 1 | 0 | 1-158043625 | NA |
| Paigen1 | finalbw | 4 | 45658185 | 6.7 | 45658185 | 45726104 | 1 | 1 | rs13477679 | NA |
| Paigen1 | finalbw | 4 | 135299463 | 6.6 | 135299463 | 135740473 | 2 | 1 | 4-134485402 | NA |
| Paigen1 | finalbw | 9 | 40887472 | 6.7 | 40887472 | 40887472 | 1 | 0 | 9-41203095 | 2810457I06Rik:72828:intron:1:20894:105927 |
| Paigen1 | finalbw | 13 | 8305114 | 7.9 | 7027401 | 10839186 | 22 | 28 | 13-8160005 | NA |
| Paigen1 | free_to_est | 1 | 14345537 | 6.7 | 14345537 | 14345537 | 1 | 0 | rs13475733 | NA |
| Paigen1 | free_to_est | 1 | 123693482 | 6.7 | 122514868 | 123779579 | 1 | 9 | 1-121650208 | NA |
| Paigen1 | free_to_est | 4 | 34853745 | 7.3 | 34368399 | 35463813 | 3 | 12 | 4-34756491 | Slc35a1:24060:intron:7:4067:4694 |
| Paigen1 | free_to_est | 4 | 54792514 | 6.6 | 54792514 | 54792514 | 1 | 0 | 4-54016459 | NA |
| Paigen1 | free_to_est | 5 | 46816524 | 7.9 | 46183643 | 48203898 | 3 | 25 | 5-45153064 | NA |
| Paigen1 | free_to_est | 9 | 44074248 | 6.8 | 44074248 | 44074248 | 1 | 0 | 9-44427228 | NA |
| Paigen1 | free_to_est | 14 | 73922389 | 7.2 | 73790696 | 73955530 | 1 | 4 | 14-66950365 | Lcp1:18826:intron:2:11300:21142 |
| Paigen1 | free_to_est | 18 | 30594244 | 6.8 | 30594244 | 30923554 | 1 | 6 | 18-30899256 | NA |
| Paigen1 | freeHCC | 1 | 3119136 | 6.6 | 3119110 | 3428294 | 3 | 5 | 1-3133070 | NA |
| Paigen1 | freeHCC | 1 | 76273244 | 6.0 | 75913252 | 76284660 | 1 | 4 | rs3699038 | NA |
| Paigen1 | freeHCC | 1 | 184178515 | 6.5 | 183910973 | 184373349 | 6 | 3 | rs6208459 | NA |
| Paigen1 | freeHCC | 3 | 43848221 | 6.0 | 43368934 | 44332429 | 1 | 18 | 3-43587878 | NA |
| Paigen1 | freeHCC | 3 | 102402952 | 6.1 | 102376279 | 102680697 | 3 | 4 | 3-102205596 | NA |
| Paigen1 | freeHCC | 3 | 105294552 | 8.7 | 105281269 | 106054197 | 11 | 4 | 3-105484341 | NA |
| Paigen1 | freeHCC | 3 | 111719078 | 6.2 | 111669652 | 111743917 | 2 | 4 | 3-111984306 | NA |
| Paigen1 | freeHCC | 3 | 116895135 | 6.6 | 116675714 | 116906664 | 1 | 6 | rs13477334 | Frrs1:20321:exon:17:62:91 |
| Paigen1 | freeHCC | 3 | 144337710 | 6.0 | 144337710 | 144337710 | 1 | 0 | 3-144967138 | NA |
| Paigen1 | freeHCC | 4 | 12723277 | 5.9 | 12723277 | 12723277 | 1 | 0 | 4-12722453 | NA |
| Paigen1 | freeHCC | 4 | 91027391 | 6.1 | 91027391 | 91027391 | 1 | 0 | 4-89865648 | NA |
| Paigen1 | freeHCC | 4 | 93247345 | 6.1 | 93247345 | 93255673 | 2 | 0 | 4-92085602 | NA |
| Paigen1 | freeHCC | 5 | 38712761 | 6.8 | 37186391 | 40408857 | 20 | 30 | 5-36986669 | Slc2a9:117591:intron:6:12027:19231 |
| Paigen1 | freeHCC | 5 | 148205561 | 6.6 | 148159961 | 148221226 | 1 | 2 | 5-145220044 | NA |
| Paigen1 | freeHCC | 6 | 114649833 | 6.7 | 114649833 | 114649833 | 1 | 0 | 6-115073163 | Atg7:74244:intron:5:3970:4160 |
| Paigen1 | freeHCC | 9 | 99901066 | 7.8 | 99555927 | 99985515 | 5 | 3 | rs13480371 | NA |
| Paigen1 | freeHCC | 11 | 63027458 | 7.3 | 62997608 | 64418704 | 7 | 6 | 11-62823811 | NA |
| Paigen1 | freeHCC | 11 | 66473338 | 6.5 | 66473338 | 66487233 | 2 | 2 | 11-66269691 | NA |
| Paigen1 | freeHCC | 11 | 81307680 | 6.5 | 81307680 | 81324169 | 1 | 1 | 11-81104041 | NA |
| Paigen1 | freeHCC | 12 | 7813147 | 7.1 | 7813147 | 8364522 | 3 | 2 | rs13481288 | NA |
| Paigen1 | freeHCC | 12 | 13127533 | 6.5 | 13048617 | 13134858 | 1 | 3 | 12-13227300 | NA |
| Paigen1 | freeHCC | 12 | 48991400 | 6.8 | 48991400 | 49327274 | 2 | 7 | 12-43324158 | NA |
| Paigen1 | freeHCC | 12 | 54399696 | 7.7 | 54379358 | 54546639 | 3 | 1 | 12-48702047 | Npas3:27386:intron:2:123634:146597 |
| Paigen1 | freeHCC | 13 | 54266646 | 6.6 | 53289717 | 55026945 | 1 | 31 | 13-53301774 | NA |
| Paigen1 | freeHCC | 14 | 46916766 | 6.9 | 46916766 | 46916766 | 1 | 0 | 14-40664963 | NA |
| Paigen1 | freeHCC | 15 | 66074305 | 6.8 | 66074305 | 66836172 | 3 | 1 | rs13482620 | Kcnq3:110862:intron:1:41198:254359 |
| Paigen1 | freeHCC | 15 | 74708021 | 9.2 | 71964762 | 75260126 | 28 | 32 | 15-75172835 | 2010109I03Rik:67038:e20on:2:6:97 |
| Paigen1 | freeHCC | 15 | 98464713 | 6.4 | 97462536 | 98596869 | 9 | 20 | 15-99097476 | Cacnb3:12297:intron:1:1464:4273 |
| Paigen1 | freeHCC | 15 | 101357816 | 6.5 | 101246295 | 102040251 | 2 | 6 | 15-102009801 | Krt2-16:16680:intron:3:427:530 |
| Paigen1 | freeHCC | 17 | 4556860 | 5.9 | 4556860 | 4556860 | 1 | 0 | 17-4599406 | NA |
| Paigen1 | freeHCC | 19 | 9911326 | 6.0 | 9518092 | 10132799 | 1 | 5 | 19-8943362 | NA |
| Paigen1 | freeHCC | 20 | 95718919 | 6.5 | 95266780 | 95718919 | 1 | 3 | 20-90919779 | NA |
| Paigen1 | fusedLC | 1 | 43401224 | 7.5 | 43381192 | 44655310 | 2 | 7 | 1-43785101 | Nck2:17974:intron:1:10649:63591 |
| Paigen1 | gbvol | 1 | 77031248 | 6.6 | 77031248 | 77159401 | 1 | 2 | 1-77605363 | NA |
| Paigen1 | gbvol | 4 | 139201429 | 7.9 | 138629266 | 139841280 | 20 | 34 | 4-138385655 | NA |
| Paigen1 | gbvol | 5 | 49856249 | 6.2 | 49856249 | 49856249 | 1 | 0 | rs13478244 | NA |
| Paigen1 | gbvol | 5 | 75495077 | 6.4 | 75441917 | 75495077 | 1 | 2 | 5-73908836 | NA |
| Paigen1 | gbvol | 5 | 77738278 | 6.3 | 77738278 | 77738278 | 1 | 0 | 5-75396910 | NA |
| Paigen1 | gbvol | 6 | 48542123 | 6.4 | 48542123 | 49036367 | 3 | 5 | 6-48524415 | NA |
| Paigen1 | gbvol | 6 | 73032676 | 7.3 | 72348266 | 73237720 | 1 | 3 | rs13478818 | Dnahc6:330355:exon:25:103:124 |
| Paigen1 | gbvol | 6 | 144114305 | 6.1 | 144114305 | 144114305 | 1 | 0 | 6-144307894 | So205:20678:intron:1:52184:54106 |
| Paigen1 | gbvol | 8 | 29083094 | 6.1 | 28964812 | 29083094 | 1 | 1 | rs13479665 | Gm1698:382003:intron:5:4579:7599 |
| Paigen1 | gbvol | 8 | 64022793 | 6.5 | 63139808 | 64405458 | 1 | 7 | 8-60029438 | Nek1:18004:UTR:36:493:1196 |
| Paigen1 | gbvol | 8 | 67929827 | 7.3 | 66894940 | 68101188 | 3 | 8 | rs13479805 | Trim60:234329:exon:1:756:1400 |
| Paigen1 | gbvol | 8 | 71557622 | 6.3 | 71491084 | 71557622 | 2 | 0 | 8-67653849 | 4732435N03Rik:234356:intron:2:17164:88676 |
| Paigen1 | gbvol | 11 | 22779376 | 6.2 | 22779376 | 22790872 | 1 | 1 | 11-22774943 | NA |
| Paigen1 | gbvol | 14 | 59761263 | 7.7 | 58739760 | 59772520 | 3 | 9 | 14-52801333 | Mipep:70478:intron:7:4183:5997 |
| Paigen1 | gbvol | 14 | 73830646 | 6.7 | 73387192 | 73830646 | 1 | 1 | 14-66858622 | 4921509B22Rik:70859:intron:9:1731:11884 |
| Paigen1 | gbvol | 14 | 95784673 | 7.2 | 93238933 | 95969652 | 5 | 31 | rs13482325 | NA |
| Paigen1 | gbvol | 17 | 56681393 | 7.1 | 56647034 | 56715191 | 1 | 6 | rs4231558 | NA |
| Paigen1 | gbvol | 19 | 3413095 | 6.2 | 3413095 | 3413095 | 1 | 0 | rs13483499 | Gal:14419:intron:3:199:1646 |
| Paigen1 | gbvol | 19 | 56056309 | 6.6 | 56056309 | 56056309 | 1 | 0 | 19-55384743 | NA |
| Paigen1 | HDL_Ch | 1 | 170583608 | 7.0 | 170015269 | 171141850 | 1 | 12 | 1-168818379 | NA |
| Paigen1 | HDL_Ch | 1 | 173030894 | 7.0 | 173030894 | 174313446 | 1 | 13 | 1-171265451 | Pcp4l1:66425:intron:2:1790:20384 |
| Paigen1 | HDL_Ch | 1 | 192123469 | 6.5 | 192123469 | 192123469 | 1 | 0 | 1-190298609 | NA |
| Paigen1 | HDL_Ch | 2 | 151526684 | 8.5 | 151526684 | 151882453 | 1 | 2 | rs3662117 | NA |
| Paigen1 | HDL_Ch | 8 | 58660356 | 6.7 | 58660356 | 59468048 | 2 | 5 | 8-54575372 | NA |
| Paigen1 | HDL_Ch | 12 | 54757646 | 7.1 | 54757646 | 55174687 | 1 | 1 | rs4135641 | Npas3:27386:intron:5:4233:115375 |
| Paigen1 | inflammation | 5 | 92156618 | 5.4 | 92156618 | 92254844 | 1 | 11 | 5-89895239 | Ereg:13874:intron:1:6612:11969 |
| Paigen1 | inflammation | 5 | 137380999 | 5.5 | 137266034 | 137380999 | 1 | 3 | 5-134546144 | NA |
| Paigen1 | inflammation | 7 | 11033063 | 5.7 | 11033063 | 11059221 | 2 | 0 | 7-6058978 | NA |
| Paigen1 | inflammation | 7 | 40063399 | 6.3 | 40063399 | 40074652 | 2 | 0 | 7-27725351 | NA |
| Paigen1 | inflammation | 7 | 57586787 | 5.6 | 57114967 | 57963015 | 3 | 2 | 7-45318522 | Gabrb3:14402:intron:4:127083:173583 |
| Paigen1 | inflammation | 7 | 99202459 | 6.5 | 96612374 | 101824216 | 14 | 126 | 7-86603120 | Mtap6:17760:intron:3:10707:18315 |
| Paigen1 | inflammation | 8 | 117675798 | 5.7 | 117468254 | 117675798 | 1 | 2 | 8-114160216 | Wwo20:80707:intron:8:77569:639384 |
| Paigen1 | inflammation | 18 | 75216645 | 6.1 | 75027530 | 75668994 | 2 | 4 | 18-75622455 | Dym:69190:intron:8:8772:32280 |
| Paigen1 | initbw | 1 | 154888397 | 11.4 | 153497361 | 154997648 | 5 | 6 | 1-153100355 | Lamc2:16782:intron:22:635:2530 |
| Paigen1 | initbw | 2 | 34420176 | 7.4 | 34420176 | 34420176 | 1 | 0 | 2-34558158 | Mapkap1:227743:intron:9:606:22283 |
| Paigen1 | initbw | 2 | 132434284 | 8.5 | 132150729 | 132434284 | 1 | 2 | 2-132491866 | 1110034G24Rik:73747:intron:4:50710:58079 |
| Paigen1 | initbw | 4 | 45658185 | 8.0 | 45658185 | 46561599 | 1 | 4 | rs13477679 | NA |
| Paigen1 | initbw | 5 | 3973102 | 7.9 | 3944283 | 4055616 | 7 | 1 | 5-3973101 | Akap9:100986:intron:7:4638:6121 |
| Paigen1 | initbw | 13 | 10065064 | 8.6 | 7027401 | 10839186 | 19 | 18 | rs6329684 | Chrm3:12671:intron:3:56408:93726 |
| Paigen1 | initbw | 20 | 134698943 | 8.3 | 134698943 | 134698943 | 1 | 0 | 20-129359741 | NA |
| Paigen1 | liverweight | 2 | 34445470 | 13.2 | 30234845 | 34846644 | 57 | 37 | rs13476425 | Mapkap1:227743:exon:11:37:125 |
| Paigen1 | liverweight | 4 | 11634316 | 7.4 | 11583171 | 11658612 | 1 | 6 | rs6403469 | Gem:14579:intron:2:927:4895 |
| Paigen1 | liverweight | 5 | 62003440 | 10.2 | 60433124 | 62863940 | 5 | 25 | 5-60365570 | NA |
| Paigen1 | liverweight | 6 | 81430539 | 6.9 | 80588524 | 82033412 | 2 | 16 | 6-81690120 | NA |
| Paigen1 | liverweight | 7 | 50734266 | 8.1 | 50692833 | 50734266 | 1 | 1 | 7-38311812 | NA |
| Paigen1 | liverweight | 7 | 76167746 | 8.4 | 76167746 | 76721657 | 2 | 2 | 7-63362598 | NA |
| Paigen1 | liverweight | 7 | 123603175 | 9.6 | 122495184 | 123712654 | 10 | 8 | rs6275579 | NA |
| Paigen1 | liverweight | 11 | 4552514 | 7.3 | 4137238 | 4611610 | 3 | 4 | 11-4547299 | Ascc2:75452:intron:5:2641:6397 |
| Paigen1 | liverweight | 18 | 16917241 | 8.2 | 16917241 | 16929356 | 1 | 1 | rs3724798 | Cdh2:12558:intron:2:284:124008 |
| Paigen1 | liverweight | 18 | 21618655 | 8.0 | 21618655 | 21618655 | 1 | 0 | rs29770217 | NA |
| Paigen1 | macrovacs | 9 | 18024803 | 8.3 | 18024803 | 18024803 | 1 | 0 | 9-18122743 | NA |
| Paigen1 | macrovacs | 11 | 109188958 | 8.0 | 109188958 | 109188958 | 1 | 0 | 11-109043993 | Gna13:14674:intron:2:7105:26582 |
| Paigen1 | microvacs | 12 | 14226272 | 5.6 | 14185981 | 14226272 | 1 | 1 | 12-14322769 | NA |
| Paigen1 | mucin | 7 | 115314697 | 6.5 | 115065765 | 116033095 | 5 | 6 | 7-102809094 | So206:20679:intron:12:18073:28851 |
| Paigen1 | mucin | 14 | 17693238 | 6.3 | 17693238 | 17693238 | 1 | 0 | 14-15567442 | NA |
| Paigen1 | nonHDL_Ch | 2 | 102722658 | 7.5 | 102722658 | 102854379 | 1 | 2 | 2-102815156 | NA |
| Paigen1 | nonHDL_Ch | 8 | 94625958 | 7.2 | 94625958 | 94841842 | 1 | 1 | 8-91058501 | NA |
| Paigen1 | nonHDL_Ch | 11 | 65391336 | 6.8 | 64827488 | 65391336 | 1 | 2 | 11-65187689 | NA |
| Paigen1 | nonHDL_Ch | 17 | 38356154 | 8.1 | 38057208 | 39400265 | 1 | 9 | 17-37307797 | NA |
| Paigen1 | nonHDL_Ch | 20 | 103213376 | 6.7 | 103213376 | 103213376 | 1 | 0 | 20-98415013 | NA |
| Paigen1 | pctHDL-TCH | 1 | 124458942 | 6.8 | 124427063 | 124459054 | 1 | 2 | 1-122425323 | NA |
| Paigen1 | pctHDL-TCH | 1 | 148100184 | 7.6 | 147367342 | 150652141 | 5 | 24 | 1-146244337 | NA |
| Paigen1 | pctHDL-TCH | 1 | 173030894 | 7.9 | 173030894 | 173975552 | 4 | 6 | 1-171265451 | Pcp4l1:66425:intron:2:1790:20384 |
| Paigen1 | pctHDL-TCH | 4 | 51265008 | 6.8 | 51265008 | 51265008 | 1 | 0 | 4-50488953 | NA |
| Paigen1 | pctHDL-TCH | 5 | 107334949 | 7.2 | 107334949 | 107334949 | 1 | 0 | 5-104529234 | NA |
| Paigen1 | pctHDL-TCH | 6 | 135925723 | 8.0 | 134818925 | 136653496 | 13 | 22 | 6-136019292 | Grin2b:14812:intron:1:83859:120418 |
| Paigen1 | pctHDL-TCH | 7 | 112653214 | 6.9 | 112311149 | 112655155 | 2 | 1 | 7-100132354 | Tead1:21676:intron:9:476:14517 |
| Paigen1 | pctHDL-TCH | 8 | 58788222 | 8.0 | 58660356 | 58788222 | 2 | 0 | 8-54702286 | Adam29:244486:intron:1:10771:33310 |
| Paigen1 | pctHDL-TCH | 8 | 85096340 | 7.0 | 85096340 | 85195538 | 2 | 1 | 8-81443534 | NA |
| Paigen1 | pctHDL-TCH | 8 | 94625958 | 7.1 | 94522459 | 94625958 | 1 | 1 | 8-91058501 | NA |
| Paigen1 | pctHDL-TCH | 11 | 67030852 | 6.9 | 64911792 | 67030852 | 2 | 1 | 11-66827205 | Myh1:17879:intron:25:4:83 |
| Paigen1 | pctHDL-TCH | 13 | 76300033 | 7.0 | 76300033 | 76897914 | 1 | 2 | 13-72286696 | NA |
| Paigen1 | pctHDL-TCH | 14 | 48678201 | 8.5 | 48536850 | 50108039 | 7 | 6 | 14-42433522 | 3632451O06Rik:67419:intron:4:12157:16724 |
| Paigen1 | pctHDL-TCH | 20 | 66120325 | 7.1 | 66094606 | 66120325 | 1 | 1 | 20-61426007 | Aff2:14266:intron:15:131:4390 |
| Paigen1 | totalCh | 2 | 152229161 | 6.2 | 152094989 | 152516660 | 1 | 9 | 2-152601069 | NA |
| Paigen1 | totalCh | 15 | 96433855 | 6.0 | 96403911 | 96433855 | 1 | 1 | 15-97033929 | Slc38a1:105727:intron:3:4034:17273 |
| Paigen1 | totalCh | 18 | 73271165 | 6.1 | 73262577 | 73271165 | 1 | 1 | 18-73675325 | NA |
| Paigen1 | totHCC | 1 | 3095984 | 8.4 | 3051362 | 3468473 | 5 | 14 | 1-3109918 | NA |
| Paigen1 | totHCC | 1 | 9014287 | 7.1 | 8932764 | 9510850 | 5 | 1 | 1-9092983 | Sntg1:71096:intron:2:178825:203928 |
| Paigen1 | totHCC | 3 | 100173183 | 6.6 | 100173183 | 100173183 | 1 | 0 | 3-99981989 | Spag17:74362:intron:11:5428:10439 |
| Paigen1 | totHCC | 4 | 101028246 | 7.0 | 101028246 | 101028246 | 1 | 0 | 4-99875955 | NA |
| Paigen1 | totHCC | 5 | 50546302 | 6.6 | 50546302 | 50546302 | 1 | 0 | 5-48871927 | NA |
| Paigen1 | totHCC | 6 | 91398074 | 7.7 | 91398074 | 91404534 | 1 | 1 | 6-91784244 | NA |
| Paigen1 | totHCC | 6 | 116114107 | 6.9 | 114679582 | 116211040 | 2 | 2 | 6-116535908 | NA |
| Paigen1 | totHCC | 8 | 114944590 | 8.0 | 114237976 | 115274070 | 2 | 4 | 8-111399139 | NA |
| Paigen1 | totHCC | 10 | 17632967 | 7.4 | 16712176 | 17712868 | 3 | 7 | rs3712394 | Heca:380629:intron:1:3957:31474 |
| Paigen1 | totHCC | 11 | 15648344 | 6.6 | 15220364 | 15648344 | 1 | 2 | 11-15643911 | NA |
| Paigen1 | totHCC | 12 | 104441364 | 8.3 | 104441364 | 104441364 | 1 | 0 | 12-98749306 | Serpina12:68054:intron:1:3947:5873 |
| Paigen1 | totHCC | 12 | 113547457 | 6.5 | 113547457 | 113547457 | 1 | 0 | 12-107853312 | Mta1:116870:intron:1:1202:13698 |
| Paigen1 | totHCC | 13 | 39189984 | 6.9 | 39189984 | 39189984 | 1 | 0 | 13-38625063 | NA |
| Paigen1 | totHCC | 15 | 25503874 | 8.3 | 25503874 | 25503874 | 1 | 0 | 15-25601255 | NA |
| Paigen1 | totHCC | 20 | 152459181 | 7.5 | 152428352 | 152459181 | 1 | 1 | 20-147034025 | NA |
| Paigen2 | CHOL | 1 | 64687098 | 8.6 | 64687098 | 64999299 | 1 | 3 | 1-65153845 | NA |
| Paigen2 | CHOL | 10 | 12658511 | 8.6 | 12658511 | 12658511 | 1 | 0 | 10-12679092 | NA |
| Paigen2 | CHOL | 15 | 49069768 | 6.7 | 49069768 | 49069768 | 1 | 0 | 15-49263657 | NA |
| Paigen2 | CHOL | 15 | 58704129 | 6.8 | 58704129 | 58704129 | 1 | 0 | rs3698351 | Trmt12:68260:UTR:1:18:2750 |
| Paigen2 | CHOL_chg | 1 | 127775854 | 6.6 | 127483234 | 127937637 | 1 | 5 | 1-125779604 | E030049G20Rik:210356:intron:14:28616:61903 |
| Paigen2 | CHOL_chg | 7 | 78416551 | 6.8 | 78095482 | 78416551 | 1 | 1 | 7-65605148 | Ntrk3:18213:intron:1:34496:60040 |
| Paigen2 | CHOL_chg | 7 | 80906674 | 6.6 | 80906674 | 81263386 | 1 | 7 | 7-68119773 | Zfp592:233410:intron:4:3533:7720 |
| Paigen2 | CHOL_chg | 14 | 11554222 | 7.0 | 10548061 | 12298656 | 2 | 4 | 14-9364412 | Cadps:27062:intron:1:61347:106906 |
| Paigen2 | CHOL_fat17 | 1 | 4978456 | 6.9 | 4978456 | 4978456 | 1 | 0 | 1-5001401 | Rgs20:58175:intron:2:30934:95320 |
| Paigen2 | CHOL_fat17 | 1 | 127218849 | 8.8 | 126717752 | 127937637 | 8 | 24 | 1-125215583 | NA |
| Paigen2 | CHOL_fat17 | 1 | 166221315 | 6.0 | 166221315 | 166369053 | 1 | 1 | 1-164401681 | Nme7:171567:intron:12:3930:4989 |
| Paigen2 | CHOL_fat17 | 1 | 186718925 | 5.9 | 186718925 | 186718925 | 1 | 0 | 1-184654904 | NA |
| Paigen2 | CHOL_fat17 | 2 | 102987850 | 7.8 | 102452012 | 104187729 | 7 | 16 | 2-103080059 | NA |
| Paigen2 | CHOL_fat17 | 3 | 71358632 | 8.2 | 70772289 | 71358632 | 2 | 3 | 3-71611384 | NA |
| Paigen2 | CHOL_fat17 | 3 | 122080181 | 6.6 | 121776642 | 122080181 | 1 | 1 | 3-122469753 | Abca4:11304:intron:11:666:15182 |
| Paigen2 | CHOL_fat17 | 4 | 118611361 | 6.0 | 118578774 | 118611361 | 1 | 1 | 4-117459070 | Slc2a1:20525:intron:1:4456:11417 |
| Paigen2 | CHOL_fat17 | 5 | 59721911 | 6.8 | 59721911 | 59721911 | 1 | 0 | 5-58083945 | NA |
| Paigen2 | CHOL_fat17 | 6 | 128038835 | 7.9 | 128032613 | 129933124 | 2 | 2 | 6-128726296 | Tspan9:109246:intron:2:10164:49135 |
| Paigen2 | CHOL_fat17 | 6 | 144422018 | 6.1 | 143989104 | 144422018 | 1 | 10 | 6-144616330 | NA |
| Paigen2 | CHOL_fat17 | 7 | 101799070 | 6.8 | 101799070 | 101799070 | 1 | 0 | 7-89269932 | Lrrc51:69358:intron:3:2142:5824 |
| Paigen2 | CHOL_fat17 | 8 | 117525955 | 6.4 | 117525955 | 117553832 | 1 | 1 | 8-114010043 | Wwo20:80707:intron:5:150972:190780 |
| Paigen2 | CHOL_fat17 | 10 | 103515696 | 7.3 | 102802944 | 104718852 | 2 | 30 | 10-103797288 | NA |
| Paigen2 | CHOL_fat17 | 11 | 70080724 | 6.1 | 70080724 | 70080724 | 1 | 0 | 11-69877085 | NA |
| Paigen2 | CHOL_fat17 | 11 | 85561821 | 6.8 | 85199298 | 85561821 | 2 | 1 | 11-85347291 | Bcas3:192197:intron:24:161630:217789 |
| Paigen2 | CHOL_fat17 | 12 | 88914002 | 6.0 | 88914002 | 89255770 | 1 | 4 | 12-82975188 | Adck1:72113:intron:3:28967:30416 |
| Paigen2 | CHOL_fat17 | 14 | 77689499 | 7.0 | 77257163 | 77689499 | 2 | 2 | 14-70728226 | 1300010F03Rik:219189:intron:13:5766:32656 |
| Paigen2 | CHOL_fat17 | 14 | 94836090 | 5.9 | 94392130 | 94966338 | 1 | 4 | 14-88122270 | NA |
| Paigen2 | CHOL_fat17 | 15 | 96377977 | 7.0 | 96377977 | 96377977 | 1 | 0 | 15-96977946 | NA |
| Paigen2 | CHOL_fat17 | 16 | 10541744 | 6.0 | 10541744 | 10541744 | 1 | 0 | 16-10319195 | 4932416N17Rik:74374:intron:14:663:2541 |
| Paigen2 | CHOL_fat17 | 16 | 44583261 | 6.3 | 44583261 | 44583261 | 1 | 0 | 16-44560050 | NA |
| Paigen2 | CHOL_fat17 | 16 | 88746555 | 8.3 | 88746555 | 88764449 | 1 | 1 | 16-89287772 | NA |
| Paigen2 | CHOL_fat17 | 17 | 77384047 | 7.0 | 77384047 | 77384047 | 1 | 0 | 17-76016189 | NA |
| Paigen2 | CHOL_fat17 | 17 | 84354063 | 5.9 | 84025092 | 84354063 | 1 | 1 | 17-83019443 | Thada:240174:intron:10:767:1994 |
| Paigen2 | CHOL_fat17 | 18 | 73660802 | 6.2 | 73660802 | 74263044 | 1 | 11 | 18-74062053 | NA |
| Paigen2 | CHOL_fat17 | 19 | 17574852 | 7.3 | 16876300 | 18027112 | 2 | 14 | 19-16787044 | Pcsk5:18552:intron:25:2333:32250 |
| Paigen2 | CHOL_fat17 | 19 | 24858841 | 7.2 | 23986818 | 25037964 | 3 | 22 | 19-24066552 | Pgm5:226041:intron:6:23912:65176 |
| Paigen2 | HDLC | 1 | 64687098 | 8.2 | 64687098 | 64999299 | 3 | 2 | 1-65153845 | NA |
| Paigen2 | HDLC | 10 | 12658511 | 7.3 | 12658511 | 12658511 | 1 | 0 | 10-12679092 | NA |
| Paigen2 | HDLC_chg | 6 | 78306976 | 7.3 | 78306976 | 78583609 | 1 | 1 | rs13478842 | Reg3d:30053:exon:3:73:117 |
| Paigen2 | HDLC_chg | 8 | 104917370 | 6.6 | 104917370 | 104917370 | 1 | 0 | 8-101377158 | NA |
| Paigen2 | HDLC_chg | 11 | 7358196 | 6.1 | 7358196 | 7358196 | 1 | 0 | 11-7352986 | NA |
| Paigen2 | HDLC_chg | 15 | 66070880 | 6.4 | 64138215 | 66088309 | 2 | 8 | 15-66509343 | Kcnq3:110862:intron:1:44624:254359 |
| Paigen2 | HDLC_chg | 18 | 43701826 | 7.3 | 43701826 | 44963629 | 10 | 2 | 18-44017459 | Jakmip2:76217:intron:4:803:1738 |
| Paigen2 | HDLC_chg | 19 | 23832333 | 6.6 | 23832333 | 23832333 | 1 | 0 | 19-23037952 | Apba1:319924:intron:1:6143:133642 |
| Paigen2 | HDLC_fat17 | 1 | 64687098 | 10.3 | 64616613 | 65010863 | 1 | 6 | 1-65153845 | NA |
| Paigen2 | HDLC_fat17 | 2 | 150743514 | 7.6 | 150743514 | 150743514 | 1 | 0 | 2-151115422 | NA |
| Paigen2 | HDLC_fat17 | 3 | 107598757 | 7.2 | 107598757 | 107605715 | 1 | 1 | rs3684333 | Slc6a17:229706:UTR:12:478:3631 |
| Paigen2 | HDLC_fat17 | 3 | 118114138 | 7.8 | 118114138 | 118114138 | 1 | 0 | 3-118472735 | NA |
| Paigen2 | HDLC_fat17 | 4 | 89180189 | 9.4 | 88853221 | 89256571 | 5 | 2 | 4-88017669 | NA |
| Paigen2 | HDLC_fat17 | 5 | 40970004 | 6.7 | 40970004 | 40970004 | 1 | 0 | 5-39247079 | NA |
| Paigen2 | HDLC_fat17 | 5 | 109113200 | 10.0 | 109113200 | 110945537 | 4 | 1 | 5-106320545 | NA |
| Paigen2 | HDLC_fat17 | 11 | 7657435 | 6.6 | 5855128 | 7971848 | 3 | 6 | 11-7652225 | NA |
| Paigen2 | nonHDL | 4 | 50934202 | 6.5 | 50934202 | 51030916 | 1 | 1 | 4-50158147 | NA |
| Paigen2 | nonHDL_fat17 | 1 | 4978456 | 6.4 | 4978456 | 5315955 | 1 | 2 | 1-5001401 | Rgs20:58175:intron:2:30934:95320 |
| Paigen2 | nonHDL_fat17 | 1 | 109408414 | 6.2 | 109403226 | 109602513 | 1 | 2 | 1-107586088 | NA |
| Paigen2 | nonHDL_fat17 | 1 | 127775854 | 9.9 | 126717752 | 128526806 | 25 | 46 | 1-125779604 | E030049G20Rik:210356:intron:14:28616:61903 |
| Paigen2 | nonHDL_fat17 | 1 | 188957331 | 6.0 | 188329619 | 189124305 | 1 | 6 | 1-186902757 | Gpatc2:67769:intron:5:22716:70502 |
| Paigen2 | nonHDL_fat17 | 2 | 102987850 | 8.9 | 102450929 | 104187729 | 10 | 20 | 2-103080059 | NA |
| Paigen2 | nonHDL_fat17 | 3 | 70772289 | 8.6 | 70772289 | 71358632 | 2 | 2 | 3-71038808 | NA |
| Paigen2 | nonHDL_fat17 | 3 | 80936737 | 6.1 | 80936737 | 80936737 | 1 | 0 | 3-81243756 | Glrb:14658:intron:9:97:5649 |
| Paigen2 | nonHDL_fat17 | 3 | 85229449 | 6.7 | 85137127 | 85230294 | 1 | 2 | 3-85605829 | NA |
| Paigen2 | nonHDL_fat17 | 3 | 122080181 | 6.1 | 122080181 | 122080181 | 1 | 0 | 3-122469753 | Abca4:11304:intron:11:666:15182 |
| Paigen2 | nonHDL_fat17 | 4 | 75711259 | 7.8 | 75618266 | 76289386 | 12 | 23 | 4-74514274 | Ptprd:19266:intron:4:3615:102941 |
| Paigen2 | nonHDL_fat17 | 4 | 85674689 | 6.0 | 85674689 | 85674689 | 1 | 0 | 4-84477908 | Adamtsl1:77739:intron:5:4054:13221 |
| Paigen2 | nonHDL_fat17 | 4 | 118611361 | 7.7 | 118578774 | 118611386 | 1 | 2 | 4-117459070 | Slc2a1:20525:intron:1:4456:11417 |
| Paigen2 | nonHDL_fat17 | 5 | 16119658 | 7.1 | 16119658 | 17467643 | 2 | 0 | 5-14968695 | Hgf:15234:intron:12:2911:5183 |
| Paigen2 | nonHDL_fat17 | 5 | 85711131 | 6.5 | 85703838 | 85711131 | 1 | 3 | 5-83412532 | NA |
| Paigen2 | nonHDL_fat17 | 6 | 119059941 | 7.7 | 119059941 | 119771433 | 3 | 0 | 6-119485751 | Cacna1c:12288:intron:1:13603:50409 |
| Paigen2 | nonHDL_fat17 | 6 | 128038835 | 8.5 | 127989487 | 128212605 | 1 | 2 | 6-128726296 | Tspan9:109246:intron:2:10164:49135 |
| Paigen2 | nonHDL_fat17 | 6 | 145385700 | 6.2 | 145385700 | 145385700 | 1 | 0 | 6-145595030 | 4933403M22Rik:74071:intron:4:1338:2403 |
| Paigen2 | nonHDL_fat17 | 7 | 60701528 | 7.4 | 60636916 | 60905122 | 2 | 9 | 7-47805228 | NA |
| Paigen2 | nonHDL_fat17 | 7 | 63567324 | 6.9 | 63567324 | 63567324 | 1 | 0 | 7-50674280 | Otud7:170711:intron:5:2821:11457 |
| Paigen2 | nonHDL_fat17 | 7 | 118755596 | 7.2 | 117678699 | 118805029 | 3 | 28 | 7-106261274 | LOC668373:668373:intron:7:22750:29736 |
| Paigen2 | nonHDL_fat17 | 7 | 134009999 | 6.3 | 134006760 | 134009999 | 1 | 1 | 7-121796197 | Adam12:11489:intron:2:1126:37989 |
| Paigen2 | nonHDL_fat17 | 8 | 117525955 | 7.5 | 116769296 | 117675798 | 2 | 7 | 8-114010043 | Wwo20:80707:intron:5:150972:190780 |
| Paigen2 | nonHDL_fat17 | 9 | 123799254 | 6.8 | 123791443 | 123961305 | 6 | 5 | 9-123965772 | NA |
| Paigen2 | nonHDL_fat17 | 10 | 103807961 | 6.4 | 103483107 | 103886266 | 1 | 12 | 10-104082560 | NA |
| Paigen2 | nonHDL_fat17 | 11 | 85561821 | 6.8 | 84153678 | 85561821 | 1 | 52 | 11-85347291 | Bcas3:192197:intron:24:161630:217789 |
| Paigen2 | nonHDL_fat17 | 12 | 88972280 | 7.2 | 88914002 | 89255770 | 1 | 6 | 12-83032157 | Adck1:72113:intron:9:1866:2073 |
| Paigen2 | nonHDL_fat17 | 14 | 17765155 | 7.1 | 9397088 | 17942229 | 5 | 276 | 14-15639359 | NA |
| Paigen2 | nonHDL_fat17 | 14 | 69440460 | 6.0 | 69396577 | 69455557 | 1 | 4 | 14-62479996 | 20po7:65246:intron:1:1905:2527 |
| Paigen2 | nonHDL_fat17 | 14 | 77257163 | 6.4 | 77257163 | 77946910 | 2 | 3 | 14-70294259 | NA |
| Paigen2 | nonHDL_fat17 | 15 | 33475777 | 6.2 | 32640776 | 33475777 | 1 | 4 | 15-33583470 | Pgcp:54381:intron:2:33822:96728 |
| Paigen2 | nonHDL_fat17 | 16 | 44583261 | 7.2 | 44583261 | 44583261 | 1 | 0 | 16-44560050 | NA |
| Paigen2 | nonHDL_fat17 | 16 | 72150785 | 6.5 | 72150785 | 72150785 | 1 | 0 | 16-72617304 | NA |
| Paigen2 | nonHDL_fat17 | 16 | 88746555 | 7.2 | 88301221 | 88746555 | 1 | 1 | 16-89287772 | NA |
| Paigen2 | nonHDL_fat17 | 17 | 77384047 | 6.3 | 77384047 | 77384047 | 1 | 0 | 17-76016189 | NA |
| Paigen2 | nonHDL_fat17 | 17 | 86721597 | 6.3 | 86718333 | 86721597 | 1 | 1 | 17-85388602 | Epas1:13819:intron:6:3480:8563 |
| Paigen2 | nonHDL_fat17 | 19 | 17187116 | 6.9 | 16876300 | 18256550 | 5 | 15 | 19-16399308 | A230083H22Rik:353211:e20on:9:2054:6548 |
| Paigen2 | nonHDL_fat17 | 19 | 46123283 | 6.1 | 46100967 | 46123283 | 1 | 1 | 19-45410389 | Pprc1:226169:UTR:10:199:671 |
| Paigen2 | nonHDL_fat17 | 20 | 82593982 | 7.0 | 82322282 | 83201248 | 4 | 5 | 20-77892121 | NA |
| Paigen2 | TG | 2 | 164306300 | 6.8 | 163619163 | 164306300 | 1 | 3 | 2-164678208 | Wfdc6b:433502:intron:2:69:2249 |
| Paigen2 | TG | 3 | 87934227 | 7.0 | 87934227 | 87934227 | 1 | 0 | 3-88339328 | Sh2d2a:27371:intron:2:508:564 |
| Paigen2 | TG | 4 | 116740233 | 6.8 | 116740233 | 116740233 | 1 | 0 | 4-115587942 | Gm1661:381544:intron:1:9450:11406 |
| Paigen2 | TG | 5 | 54607854 | 7.5 | 54607854 | 55120371 | 3 | 3 | 5-52947335 | NA |
| Paigen2 | TG | 5 | 58464822 | 7.4 | 58027587 | 58464822 | 1 | 3 | rs6339023 | NA |
| Paigen2 | TG | 5 | 60732060 | 8.0 | 60688560 | 61039453 | 9 | 6 | rs3704920 | NA |
| Paigen2 | TG | 7 | 94877152 | 7.0 | 94594232 | 94877152 | 3 | 1 | rs13479412 | NA |
| Paigen2 | TG | 9 | 32248777 | 6.7 | 32248777 | 32248777 | 1 | 0 | 9-32485360 | Fli1:14247:intron:1:41977:64415 |
| Paigen2 | TG | 11 | 32267649 | 7.1 | 32267649 | 32651296 | 1 | 3 | 11-32263220 | G431001E03Rik:268396:intron:1:19679:23547 |
| Paigen2 | TG | 11 | 44340911 | 6.9 | 43971070 | 44361711 | 1 | 3 | 11-44137223 | NA |
| Paigen2 | TG | 13 | 77227241 | 7.9 | 77227241 | 77314155 | 2 | 0 | rs13481889 | Mctp1:78771:intron:8:3872:41536 |
| Paigen2 | TG | 15 | 79220475 | 7.4 | 79220475 | 79220643 | 1 | 1 | rs3665030 | 4732495E13Rik:223693:intron:1:9964:24470 |
| Paigen2 | TG | 17 | 65679528 | 6.9 | 65679528 | 65763738 | 1 | 1 | 17-64143579 | NA |
| Paigen2 | TG_chg | 2 | 113433586 | 6.3 | 113143900 | 113513455 | 3 | 6 | 2-113523721 | Fmn1:14260:intron:15:29457:33441 |
| Paigen2 | TG_chg | 3 | 79867406 | 6.7 | 79867406 | 79918801 | 3 | 0 | 3-80175095 | NA |
| Paigen2 | TG_chg | 4 | 133363042 | 6.2 | 133180234 | 133742909 | 1 | 2 | 4-132545592 | Aim1l:230806:UTR:17:1693:1876 |
| Paigen2 | TG_chg | 5 | 54725093 | 6.5 | 54720730 | 54725093 | 1 | 1 | 5-53064339 | NA |
| Paigen2 | TG_chg | 5 | 94474523 | 6.1 | 94008187 | 94804290 | 2 | 8 | 5-92226087 | NA |
| Paigen2 | TG_chg | 17 | 42918321 | 7.2 | 42891868 | 42918321 | 2 | 0 | 17-41603175 | Gpr116:224792:intron:21:513:2088 |
| Paigen4 | CHOL | 1 | 64687098 | 7.0 | 64687098 | 64687098 | 1 | 0 | 1-65153845 | NA |
| Paigen4 | CHOL | 1 | 134932091 | 7.2 | 134932091 | 134932091 | 1 | 0 | 1-132986495 | NA |
| Paigen4 | CHOL | 2 | 101108079 | 7.2 | 101108079 | 101121368 | 2 | 0 | 2-101201224 | NA |
| Paigen4 | CHOL | 10 | 12658511 | 6.8 | 12658511 | 12658511 | 1 | 0 | 10-12679092 | NA |
| Paigen4 | CHOL | 11 | 70914148 | 8.0 | 70910603 | 70941001 | 1 | 2 | 11-70710509 | Nalp1:195046:intron:12:916:1598 |
| Paigen4 | CHOL | 12 | 99551634 | 6.7 | 99355736 | 99551634 | 1 | 1 | 12-93765898 | NA |
| Paigen4 | CHOL | 14 | 30415467 | 7.0 | 30415467 | 30415467 | 1 | 0 | 14-27621763 | NA |
| Paigen4 | CHOL | 14 | 104208980 | 6.6 | 103830649 | 104208980 | 1 | 2 | 14-97621439 | NA |
| Paigen4 | CHOL | 19 | 57525266 | 7.7 | 57525266 | 57528694 | 2 | 0 | 19-56862096 | Trub1:72133:intron:3:7590:8271 |
| Paigen4 | HDLC | 1 | 64687098 | 9.8 | 64687098 | 64999299 | 1 | 1 | 1-65153845 | NA |
| Paigen4 | HDLC | 2 | 101121368 | 6.8 | 101108079 | 101121368 | 1 | 1 | 2-101214513 | NA |
| Paigen4 | HDLC | 2 | 168647354 | 7.2 | 168619639 | 168647354 | 1 | 2 | 2-169019267 | NA |
| Paigen4 | HDLC | 4 | 149367816 | 6.9 | 149367816 | 149742062 | 2 | 0 | 4-148409958 | Rere:68703:intron:9:13976:24806 |
| Paigen4 | HDLC | 5 | 91720123 | 6.6 | 91694908 | 92167453 | 1 | 3 | 5-89455424 | NA |
| Paigen4 | HDLC | 6 | 21893928 | 7.0 | 21893928 | 21893928 | 1 | 0 | rs13478656 | NA |
| Paigen4 | HDLC | 7 | 16003158 | 6.7 | 15923063 | 16214636 | 1 | 4 | 7-8530348 | Prkd2:101540:intron:1:772:1954 |
| Paigen4 | HDLC | 8 | 11414902 | 6.7 | 11414902 | 11414902 | 1 | 0 | 8-11421199 | Col4a2:12827:intron:18:121:553 |
| Paigen4 | HDLC | 9 | 8858845 | 7.1 | 8858845 | 8858845 | 1 | 0 | 9-8896861 | NA |
| Paigen4 | HDLC | 10 | 12658511 | 6.9 | 12658511 | 13866330 | 2 | 0 | 10-12679092 | NA |
| Paigen4 | HDLC | 11 | 8114274 | 7.2 | 8114274 | 8114274 | 1 | 0 | 11-8109064 | NA |
| Paigen4 | HDLC | 14 | 100776862 | 6.6 | 100737798 | 100776862 | 1 | 1 | 14-94120829 | Lmo7:380928:intron:12:1549:7759 |
| Paigen4 | HDLC | 14 | 104208980 | 7.8 | 103830649 | 104208980 | 1 | 1 | 14-97621439 | NA |
| Paigen4 | HDLC | 19 | 57525266 | 6.6 | 57525266 | 57528694 | 1 | 1 | 19-56862096 | Trub1:72133:intron:3:7590:8271 |
| Paigen4 | nonHDL | 18 | 7193721 | 6.8 | 7193721 | 7193721 | 1 | 0 | 18-7403335 | Armc4:74934:intron:18:17674:29548 |
| Paigen4 | TG | 1 | 16633181 | 7.3 | 16633181 | 16633181 | 1 | 0 | rs13475743 | Tceb1:67923:UTR:2:40:47 |
| Paigen4 | TG | 1 | 184247751 | 7.6 | 183733381 | 184247751 | 4 | 5 | 1-182496326 | NA |
| Paigen4 | TG | 2 | 171098930 | 8.4 | 170951901 | 171362249 | 5 | 4 | 2-171470843 | NA |
| Paigen4 | TG | 3 | 21870088 | 7.0 | 21637588 | 22257850 | 3 | 17 | 3-21362933 | NA |
| Paigen4 | TG | 3 | 53366376 | 7.9 | 53215173 | 54168587 | 2 | 5 | 3-53365762 | NA |
| Paigen4 | TG | 3 | 78988973 | 7.4 | 78988973 | 79006275 | 1 | 1 | rs3720421 | NA |
| Paigen4 | TG | 3 | 157667487 | 7.7 | 157516834 | 157667537 | 1 | 2 | 3-158348241 | NA |
| Paigen4 | TG | 4 | 22327176 | 7.6 | 22327176 | 22327176 | 1 | 0 | rs13477601 | NA |
| Paigen4 | TG | 4 | 137161336 | 7.0 | 137161336 | 137161336 | 1 | 0 | 4-136347275 | Ece1:230857:intron:1:26877:42686 |
| Paigen4 | TG | 5 | 53817933 | 7.7 | 53815931 | 53817933 | 1 | 1 | 5-52155702 | NA |
| Paigen4 | TG | 5 | 108217336 | 7.6 | 108132953 | 108217337 | 2 | 1 | rs3669104 | 2900024C23Rik:67266:intron:1:9914:62227 |
| Paigen4 | TG | 5 | 142445658 | 7.2 | 142316800 | 142448922 | 1 | 2 | rs13478567 | Sdk1:330222:intron:39:1185:5745 |
| Paigen4 | TG | 6 | 34437665 | 10.6 | 34418242 | 34552702 | 3 | 4 | rs13478705 | NA |
| Paigen4 | TG | 6 | 50629612 | 7.7 | 49058097 | 50783955 | 8 | 10 | 6-50649458 | NA |
| Paigen4 | TG | 6 | 74502069 | 7.1 | 74476573 | 74689969 | 4 | 0 | 6-74807303 | NA |
| Paigen4 | TG | 7 | 77195443 | 6.8 | 77009053 | 77195443 | 1 | 4 | 7-64390530 | NA |
| Paigen4 | TG | 7 | 84915085 | 7.1 | 84493842 | 85206137 | 2 | 12 | rs3656832 | NA |
| Paigen4 | TG | 7 | 95378101 | 8.4 | 94563566 | 95859139 | 2 | 8 | rs13479414 | NA |
| Paigen4 | TG | 8 | 34291399 | 7.4 | 34089852 | 34291399 | 2 | 1 | 8-31781527 | NA |
| Paigen4 | TG | 9 | 77717155 | 7.6 | 77631873 | 79951462 | 4 | 13 | 9-78295709 | NA |
| Paigen4 | TG | 9 | 98546056 | 6.8 | 98544687 | 98546056 | 1 | 2 | rs3720103 | NA |
| Paigen4 | TG | 10 | 13530579 | 8.0 | 13530579 | 13530579 | 1 | 0 | rs3653545 | Aig1:66253:intron:1:28236:39217 |
| Paigen4 | TG | 11 | 45174288 | 8.1 | 44738906 | 45174288 | 1 | 1 | 11-44970600 | NA |
| Paigen4 | TG | 11 | 97582177 | 7.9 | 97153073 | 97792514 | 2 | 4 | rs6169425 | Ccdc49:67480:intron:1:336:3042 |
| Paigen4 | TG | 12 | 11790056 | 7.8 | 11652871 | 11841603 | 5 | 5 | 12-11882497 | NA |
| Paigen4 | TG | 13 | 69699518 | 7.1 | 69699518 | 69699518 | 1 | 0 | 13-65660270 | NA |
| Paigen4 | TG | 13 | 111849433 | 6.7 | 111822394 | 111854209 | 1 | 2 | 13-107519021 | NA |
| Paigen4 | TG | 14 | 26715446 | 7.5 | 26195919 | 26827847 | 1 | 4 | 14-23929759 | D14Ertd171e:238988:intron:4:11899:21902 |
| Paigen4 | TG | 14 | 76692934 | 7.4 | 76692934 | 77117507 | 1 | 2 | 14-69720046 | Epsti1:108670:intron:6:18842:23694 |
| Paigen4 | TG | 15 | 87304348 | 8.3 | 87304348 | 87304348 | 1 | 0 | rs6405854 | NA |
| Paigen4 | TG | 17 | 67992558 | 7.0 | 67992558 | 67992558 | 1 | 0 | 17-66512925 | NA |
| Paigen4 | TG | 18 | 74969883 | 7.2 | 74969883 | 75339747 | 4 | 5 | 18-75375703 | NA |
| Paigen4 | TG | 19 | 46887813 | 6.8 | 46703822 | 47119113 | 1 | 10 | 19-46178180 | Cnnm2:94219:intron:1:71108:90506 |
| Paigen4 | TG | 19 | 61095458 | 6.8 | 61095458 | 61095458 | 1 | 0 | rs31110714 | Gprk5:14773:intron:3:8929:14211 |
| Paigen4 | TG | 20 | 47619383 | 9.5 | 47619383 | 47619383 | 1 | 0 | rs13483750 | NA |
| Paigen4 | TG | 20 | 70129300 | 11.9 | 67701596 | 70566936 | 3 | 6 | rs13483824 | NA |
| Paigen4 | TG | 20 | 80095324 | 8.9 | 80095324 | 80095324 | 1 | 0 | rs13483864 | Dmd:13405:intron:23:12035:25134 |
| Peters1 | CHCM | 12 | 81564676 | 6.3 | 81550066 | 81579728 | 2 | 2 | 12-75946115 | Slc39a9:328133:intron:1:851:17492 |
| Peters1 | cHGB | 9 | 92561197 | 6.2 | 92519219 | 92696361 | 1 | 5 | 9-92600997 | NA |
| Peters1 | cHGB | 11 | 41148475 | 6.6 | 41148475 | 41811294 | 1 | 2 | 11-40944787 | NA |
| Peters1 | EOS | 18 | 32484937 | 6.1 | 32484937 | 32617091 | 3 | 1 | 18-32799014 | NA |
| Peters1 | HCT | 10 | 105052303 | 6.4 | 103934785 | 105302259 | 1 | 62 | 10-105323638 | NA |
| Peters1 | LUC | 1 | 186878750 | 6.1 | 186878750 | 186878750 | 1 | 0 | rs6154379 | NA |
| Peters1 | LUC | 4 | 78662146 | 6.3 | 78457290 | 78836345 | 2 | 4 | 4-77465161 | NA |
| Peters1 | LUC | 5 | 75065970 | 6.6 | 74088483 | 75529474 | 6 | 16 | 5-73478047 | NA |
| Peters1 | LUC | 7 | 69903652 | 6.1 | 69834987 | 69919774 | 1 | 5 | 7-57069599 | NA |
| Peters1 | LUC | 7 | 96446366 | 6.0 | 96031592 | 96749181 | 1 | 7 | 7-83790495 | Odz4:23966:intron:5:17426:90250 |
| Peters1 | LUC | 7 | 99287479 | 7.1 | 99287479 | 99287479 | 1 | 0 | 7-86687834 | Gdpd5:233552:intron:3:2903:13560 |
| Peters1 | LUC | 7 | 101387846 | 7.0 | 100973794 | 101640893 | 1 | 3 | 7-88859752 | NA |
| Peters1 | LUC | 8 | 76807431 | 6.5 | 76807431 | 77590971 | 1 | 7 | 8-72943489 | NA |
| Peters1 | LUC | 9 | 100079368 | 8.2 | 99683994 | 100079368 | 4 | 0 | 9-100114841 | NA |
| Peters1 | LUC | 14 | 46182278 | 7.5 | 46018090 | 48268418 | 7 | 15 | 14-39929892 | Wdhd1:218973:intron:13:937:2679 |
| Peters1 | LUC | 16 | 52950060 | 6.0 | 52941530 | 52950060 | 1 | 2 | 16-53018300 | NA |
| Peters1 | LYM | 1 | 38461833 | 7.9 | 38461833 | 38461833 | 1 | 0 | rs13475820 | Aff3:16764:intron:6:17617:128360 |
| Peters1 | LYM | 1 | 150796635 | 7.6 | 150796635 | 150796635 | 1 | 0 | 1-148967674 | NA |
| Peters1 | LYM | 2 | 145411778 | 7.1 | 145411778 | 145411778 | 1 | 0 | 2-145472316 | NA |
| Peters1 | LYM | 3 | 32863290 | 6.8 | 32329613 | 32863290 | 1 | 3 | 3-32453110 | NA |
| Peters1 | LYM | 3 | 58772466 | 7.2 | 58632252 | 58772814 | 1 | 3 | 3-58813433 | Siah2:20439:intron:1:6858:14951 |
| Peters1 | LYM | 4 | 144603502 | 7.1 | 144595480 | 144607455 | 1 | 2 | 4-143672100 | NA |
| Peters1 | LYM | 5 | 53527053 | 7.2 | 53527053 | 53609202 | 2 | 9 | 5-51867624 | NA |
| Peters1 | LYM | 6 | 43857506 | 9.4 | 43756420 | 43926366 | 10 | 0 | 6-43879854 | NA |
| Peters1 | LYM | 7 | 75131935 | 7.7 | 74989813 | 78041494 | 7 | 5 | 7-62315910 | NA |
| Peters1 | LYM | 11 | 29296944 | 7.0 | 29296944 | 29624506 | 1 | 2 | 11-29292511 | A430106J12Rik:108686:intron:2:22335:25574 |
| Peters1 | LYM | 11 | 116805134 | 9.3 | 116664331 | 117336589 | 10 | 7 | 11-116660173 | NA |
| Peters1 | LYM | 12 | 111863046 | 7.4 | 111238378 | 111863046 | 1 | 3 | 12-106159808 | NA |
| Peters1 | LYM | 13 | 83125353 | 6.7 | 83125353 | 83560430 | 1 | 2 | 13-79268453 | NA |
| Peters1 | LYM | 13 | 88036325 | 6.8 | 86483139 | 88062829 | 3 | 7 | 13-84232309 | NA |
| Peters1 | LYM | 18 | 36864639 | 7.3 | 36864639 | 36864639 | 1 | 0 | 18-37181865 | 2410015B03Rik:71983:e20on:7:5:84 |
| Peters1 | LYM | 18 | 39804300 | 8.6 | 39804300 | 40115136 | 13 | 1 | 18-40121276 | NA |
| Peters1 | LYM | 19 | 55669722 | 8.4 | 55505556 | 55669722 | 5 | 4 | rs3709671 | Vti1a:53611:intron:7:117167:124511 |
| Peters1 | MCV | 1 | 13883228 | 6.7 | 12716832 | 13883228 | 1 | 9 | 1-14015064 | NA |
| Peters1 | MCV | 2 | 87869070 | 8.6 | 87869070 | 87869070 | 1 | 0 | 2-87962290 | NA |
| Peters1 | MCV | 2 | 106183830 | 7.1 | 106156841 | 108577716 | 4 | 34 | 2-106275723 | NA |
| Peters1 | MCV | 3 | 61567391 | 7.1 | 61567391 | 61688544 | 2 | 2 | 3-61642062 | NA |
| Peters1 | MCV | 3 | 110826543 | 7.2 | 110756465 | 110863808 | 1 | 4 | 3-111091788 | NA |
| Peters1 | MCV | 4 | 78457290 | 7.7 | 78399990 | 78836345 | 4 | 4 | 4-77260305 | NA |
| Peters1 | MCV | 4 | 83696978 | 6.6 | 81193857 | 84111685 | 1 | 32 | 4-82500229 | NA |
| Peters1 | MCV | 4 | 139695461 | 9.4 | 139072785 | 139886047 | 5 | 12 | 4-138879687 | NA |
| Peters1 | MCV | 5 | 74605196 | 7.2 | 74605196 | 75381361 | 1 | 8 | 5-73021532 | NA |
| Peters1 | MCV | 5 | 101624178 | 6.7 | 101436909 | 101808333 | 1 | 8 | 5-98731567 | NA |
| Peters1 | MCV | 7 | 44124913 | 7.2 | 44124913 | 44124913 | 1 | 0 | 7-31650456 | LOC546967:546967:intron:2:42:76 |
| Peters1 | MCV | 10 | 68065937 | 6.8 | 68058329 | 68901285 | 1 | 8 | 10-68448126 | NA |
| Peters1 | MCV | 11 | 105588046 | 6.6 | 105588046 | 105673754 | 1 | 3 | 11-105443081 | Tanc2:77097:intron:3:55965:60814 |
| Peters1 | MCV | 14 | 34215925 | 6.6 | 34215925 | 34223959 | 1 | 1 | 14-31428574 | Grid1:14803:intron:8:63398:120778 |
| Peters1 | MCV | 15 | 19851932 | 6.6 | 19354426 | 20044994 | 4 | 1 | 15-19941866 | NA |
| Peters1 | MCV | 15 | 72518497 | 6.8 | 72267006 | 72518497 | 1 | 11 | 15-72972982 | 1810044A24Rik:76510:intron:3:2262:8129 |
| Peters1 | MCV | 16 | 57687682 | 6.9 | 56331551 | 58153251 | 11 | 14 | 16-57946693 | NA |
| Peters1 | MCV | 18 | 52756908 | 6.8 | 52749932 | 53021778 | 3 | 7 | 18-53114728 | Zfp474:66758:intron:3:6689:13221 |
| Peters1 | mHGB | 5 | 138126408 | 6.0 | 138126408 | 138574830 | 1 | 1 | rs8265855 | Cyp3a13:13113:intron:11:2177:3451 |
| Peters1 | mHGB | 6 | 110759249 | 6.2 | 110609981 | 112238687 | 2 | 12 | 6-111242603 | Grm7:108073:intron:1:147084:267939 |
| Peters1 | mHGB | 7 | 16173873 | 6.0 | 16135968 | 16188145 | 1 | 2 | 7-8701201 | Ppp5c:19060:intron:3:46:5400 |
| Peters1 | mHGB | 7 | 71130717 | 6.1 | 71130717 | 71130717 | 1 | 0 | 7-58294031 | NA |
| Peters1 | mHGB | 10 | 16850791 | 6.2 | 16622412 | 17164669 | 4 | 4 | 10-16855995 | NA |
| Peters1 | mHGB | 11 | 41148475 | 7.0 | 39627152 | 41811294 | 3 | 60 | 11-40944787 | NA |
| Peters1 | mHGB | 14 | 20524892 | 6.1 | 19802110 | 20648999 | 1 | 32 | 14-18023946 | Dusp13:27389:intron:7:3763:4985 |
| Peters1 | mHGB | 18 | 71692881 | 6.8 | 71655190 | 71692881 | 1 | 1 | 18-72095289 | Dcc:13176:intron:9:7558:25917 |
| Peters1 | MONO | 1 | 25057739 | 8.1 | 25057739 | 25308050 | 2 | 0 | 1-25357380 | Bai3:210933:intron:23:4095:5012 |
| Peters1 | MONO | 1 | 177872404 | 6.6 | 177872404 | 177872404 | 1 | 0 | 1-176165975 | Pld5:319455:intron:6:9105:50784 |
| Peters1 | MONO | 2 | 25087947 | 7.1 | 25087947 | 25087947 | 1 | 0 | 2-25225960 | NA |
| Peters1 | MONO | 2 | 161504549 | 6.9 | 161504549 | 161504549 | 1 | 0 | 2-161876457 | Ptprt:19281:intron:9:88338:89310 |
| Peters1 | MONO | 4 | 90846694 | 6.9 | 90834537 | 90846694 | 1 | 3 | rs4136370 | Elavl2:15569:intron:3:17223:63043 |
| Peters1 | MONO | 4 | 127585126 | 6.6 | 127585126 | 127794311 | 1 | 7 | 4-126453138 | NA |
| Peters1 | MONO | 8 | 66246477 | 11.1 | 66240506 | 68795162 | 11 | 26 | 8-62270924 | Spock3:72902:intron:11:1965:2892 |
| Peters1 | MONO | 8 | 80374606 | 6.9 | 80371101 | 80553423 | 2 | 2 | 8-76592597 | Arhgap10:78514:intron:1:39095:66705 |
| Peters1 | MONO | 9 | 27814847 | 6.9 | 27567493 | 27847636 | 1 | 3 | 9-28016429 | Opcml:330908:intron:1:273954:612880 |
| Peters1 | MONO | 11 | 36384182 | 7.9 | 36289832 | 36384182 | 2 | 2 | 11-36221687 | Odz2:23964:intron:2:323908:479337 |
| Peters1 | MONO | 12 | 16209121 | 6.7 | 16209121 | 16209121 | 1 | 0 | 12-16307307 | NA |
| Peters1 | MONO | 15 | 73283548 | 7.1 | 72585170 | 73283548 | 3 | 3 | 15-73741979 | NA |
| Peters1 | MONO | 16 | 85825495 | 7.3 | 85825495 | 85825495 | 1 | 0 | 16-86311112 | NA |
| Peters1 | MONO | 18 | 17545359 | 6.8 | 17031255 | 18313549 | 1 | 18 | rs3090636 | NA |
| Peters1 | MONO | 19 | 18245338 | 7.3 | 18124170 | 18245338 | 1 | 1 | 19-17457490 | NA |
| Peters1 | PLT | 14 | 104313744 | 7.4 | 104313744 | 104440532 | 2 | 0 | 14-97727743 | NA |
| Peters1 | PT | 5 | 8380256 | 6.2 | 8297665 | 8399737 | 1 | 5 | rs3667728 | NA |
| Peters1 | PTT | 7 | 73984764 | 6.8 | 73984764 | 73984764 | 1 | 0 | 7-61157732 | NA |
| Peters1 | RBC | 1 | 15109168 | 8.2 | 15109168 | 15192017 | 3 | 0 | 1-15283013 | NA |
| Peters1 | RBC | 1 | 118961793 | 6.4 | 118961793 | 118961793 | 1 | 0 | 1-117154780 | NA |
| Peters1 | RBC | 1 | 162632688 | 6.6 | 162632688 | 162632688 | 1 | 0 | 1-160804613 | NA |
| Peters1 | RBC | 2 | 124989214 | 9.4 | 124822237 | 127158432 | 9 | 9 | rs6401493 | NA |
| Peters1 | RBC | 3 | 18999888 | 6.5 | 18999888 | 18999888 | 1 | 0 | 3-18474255 | NA |
| Peters1 | RBC | 4 | 99694996 | 7.2 | 99690225 | 99988298 | 11 | 4 | 4-98542705 | Ror1:26563:intron:1:100754:206568 |
| Peters1 | RBC | 4 | 105487275 | 7.2 | 105421595 | 105506531 | 4 | 2 | 4-104334984 | NA |
| Peters1 | RBC | 5 | 60520746 | 7.0 | 60520746 | 60565401 | 3 | 0 | rs3090667 | NA |
| Peters1 | RBC | 5 | 105697837 | 6.8 | 105563172 | 106271824 | 1 | 12 | 5-102852992 | Lrrc8b:433926:intron:1:41621:52883 |
| Peters1 | RBC | 5 | 111209287 | 7.9 | 109518668 | 111294671 | 6 | 2 | 5-108297780 | NA |
| Peters1 | RBC | 5 | 114112124 | 7.7 | 114112124 | 114165935 | 3 | 0 | 5-111242124 | Coro1c:23790:intron:4:1044:1337 |
| Peters1 | RBC | 5 | 127737398 | 7.8 | 127737398 | 127814861 | 1 | 2 | 5-125000806 | 4632425D07Rik:208213:intron:2:87754:102461 |
| Peters1 | RBC | 7 | 50088790 | 6.0 | 50088790 | 50118046 | 2 | 2 | 7-37665070 | B230343H07Rik:338352:intron:7:431:8453 |
| Peters1 | RBC | 10 | 14709884 | 6.6 | 14709884 | 15055840 | 1 | 3 | 10-14732521 | NA |
| Peters1 | RBC | 12 | 73806035 | 9.2 | 72621099 | 74687781 | 3 | 15 | rs13481527 | Six6os1:75801:intron:16:2510:4425 |
| Peters1 | RBC | 12 | 82105574 | 6.0 | 82105574 | 82105574 | 1 | 0 | 12-76489259 | NA |
| Peters1 | RBC | 12 | 92358233 | 6.0 | 92141832 | 92452586 | 1 | 4 | 12-86557805 | NA |
| Peters1 | RBC | 13 | 70815861 | 6.1 | 70633951 | 70815925 | 1 | 3 | 13-66788895 | NA |
| Peters1 | RBC | 14 | 63093140 | 6.0 | 62509383 | 64076398 | 1 | 21 | 14-56126644 | Msra:110265:intron:10:71396:87073 |
| Peters1 | RBC | 19 | 4923357 | 6.9 | 3438270 | 4923357 | 2 | 0 | 19-4711827 | Dpp3:75221:intron:3:326:478 |
| Peters1 | RBC | 19 | 8766199 | 7.2 | 8766199 | 8774745 | 1 | 1 | 19-7798235 | NA |
| Peters1 | RDW | 2 | 110189924 | 6.5 | 110189924 | 112085690 | 3 | 2 | rs3710221 | NA |
| Peters1 | Retic | 11 | 91224510 | 5.9 | 91224510 | 91224510 | 1 | 0 | rs13481157 | NA |
| Peters1 | Retic | 17 | 31982772 | 6.1 | 31364658 | 32511505 | 1 | 10 | rs29504336 | NA |
| Peters1 | WBC | 1 | 25653115 | 6.4 | 25653115 | 25653115 | 1 | 0 | 1-25953037 | Bai3:210933:intron:2:117434:266137 |
| Peters1 | WBC | 1 | 136790022 | 6.1 | 136686564 | 136794460 | 1 | 2 | 1-134862145 | Ube2t:67196:intron:6:1420:1608 |
| Peters1 | WBC | 2 | 117496537 | 7.7 | 117251398 | 117762374 | 3 | 3 | 2-117586672 | NA |
| Peters1 | WBC | 2 | 165461158 | 7.2 | 165461158 | 165782593 | 2 | 9 | 2-165833066 | Eya2:14049:intron:15:626:1706 |
| Peters1 | WBC | 3 | 32581493 | 8.5 | 32536889 | 34167774 | 7 | 6 | 3-32172019 | NA |
| Peters1 | WBC | 3 | 48140368 | 6.9 | 47604049 | 49959871 | 9 | 18 | 3-48053672 | NA |
| Peters1 | WBC | 3 | 82455731 | 7.6 | 82455731 | 82455731 | 1 | 0 | 3-82765607 | 5330427D05Rik:213582:intron:4:6189:7563 |
| Peters1 | WBC | 3 | 105792113 | 6.3 | 105792113 | 105792113 | 1 | 0 | 3-105981898 | Kcnd3:56543:intron:3:5282:6246 |
| Peters1 | WBC | 5 | 36120311 | 6.8 | 36120311 | 36602731 | 1 | 2 | 5-34248948 | Ablim2:231148:intron:3:905:4493 |
| Peters1 | WBC | 5 | 66387486 | 7.1 | 64817323 | 66432935 | 8 | 3 | 5-64708204 | BC013481:245945:intron:1:42164:50965 |
| Peters1 | WBC | 5 | 91951275 | 7.5 | 91945217 | 91955060 | 4 | 0 | 5-89690442 | NA |
| Peters1 | WBC | 6 | 86914766 | 6.5 | 86914766 | 86914766 | 1 | 0 | 6-87282224 | Aak1:269774:intron:7:2137:2547 |
| Peters1 | WBC | 6 | 119082781 | 6.1 | 119082781 | 119082781 | 1 | 0 | rs3724683 | NA |
| Peters1 | WBC | 8 | 69540534 | 6.1 | 69540534 | 69982295 | 1 | 1 | rs13479810 | NA |
| Peters1 | WBC | 8 | 89997044 | 6.2 | 89997044 | 89997044 | 1 | 0 | 8-86401052 | NA |
| Peters1 | WBC | 11 | 8736729 | 6.5 | 8691675 | 9193620 | 4 | 5 | rs3714397 | Pkd1l1:171395:intron:49:7819:8081 |
| Peters1 | WBC | 11 | 11952530 | 6.5 | 11952530 | 12087520 | 3 | 4 | 11-11947320 | NA |
| Peters1 | WBC | 11 | 44658040 | 6.2 | 44658040 | 44658040 | 1 | 0 | 11-44454352 | Ebf1:13591:intron:6:171147:225621 |
| Peters1 | WBC | 11 | 66061882 | 6.5 | 66060493 | 66481153 | 2 | 8 | 11-65858235 | Gm879:380702:intron:2:256964:277658 |
| Peters1 | WBC | 11 | 95981026 | 6.6 | 95981026 | 96351535 | 1 | 1 | 11-95836059 | Ttll6:237930:intron:14:5976:5983 |
| Peters1 | WBC | 12 | 56924340 | 6.7 | 56924340 | 56924340 | 1 | 0 | rs13481466 | NA |
| Peters1 | WBC | 13 | 60286807 | 6.7 | 60283279 | 60720594 | 1 | 6 | 13-59419255 | NA |
| Peters1 | WBC | 13 | 67417987 | 6.6 | 67417987 | 67417987 | 1 | 0 | 13-63990905 | Ptdss1:19210:intron:9:6315:10941 |
| Peters1 | WBC | 13 | 99958447 | 7.1 | 99194858 | 100989237 | 6 | 33 | 13-95596507 | Tnpo1:238799:intron:13:94:1409 |
| Peters1 | WBC | 14 | 98801530 | 6.3 | 98801530 | 98801530 | 1 | 0 | 14-92145464 | Klf12:16597:intron:5:23637:41793 |
| Peters1 | WBC | 17 | 38267910 | 6.1 | 38267910 | 38531331 | 1 | 2 | 17-37219553 | NA |
| Peters1 | WBC | 17 | 51209996 | 6.9 | 51209996 | 51209996 | 1 | 0 | rs13483026 | Satb1:20230:intron:8:22823:25041 |
| Peters1 | WBC | 19 | 10466217 | 7.7 | 10466217 | 10466217 | 1 | 0 | 19-9498253 | Syt7:54525:intron:1:9658:23825 |
| Peters1 | WBC | 20 | 44121397 | 6.1 | 44121397 | 44121397 | 1 | 0 | rs13483737 | Smarca1:93761:intron:6:2674:2903 |
| Tordoff1 | NaCl_pref450 | 18 | 67474564 | 7.7 | 67474564 | 67485133 | 1 | 1 | 18-67874937 | Cidea:12683:intron:1:5354:14868 |
| Tordoff3 | bleeding_time | 3 | 148884697 | 7.9 | 148769204 | 148898101 | 5 | 2 | 3-149524875 | Lphn2:99633:intron:2:7250:63936 |
| Tordoff3 | bleeding_time | 14 | 48179777 | 7.2 | 48179777 | 48180505 | 1 | 1 | 14-41932317 | NA |
| Tordoff3 | bleeding_time | 16 | 66001546 | 8.0 | 65268524 | 66305349 | 2 | 9 | 16-66360151 | NA |
| Tordoff3 | BMC | 2 | 119012421 | 9.4 | 116984238 | 119012421 | 4 | 3 | 2-119102556 | NA |
| Tordoff3 | BMC | 3 | 89649617 | 7.5 | 89649617 | 89719595 | 1 | 2 | 3-89988691 | Kcnn3:140493:intron:1:42284:43452 |
| Tordoff3 | BMC | 10 | 120748549 | 7.7 | 120097023 | 120748549 | 1 | 3 | 10-121088060 | NA |
| Tordoff3 | BMC | 13 | 11877209 | 7.5 | 11534269 | 11877209 | 1 | 2 | 13-11726381 | Ryr2:20191:intron:16:1224:13294 |
| Tordoff3 | BMC | 16 | 76984585 | 7.5 | 76984585 | 77311776 | 1 | 2 | 16-77461355 | Usp25:30940:intron:18:8250:14141 |
| Tordoff3 | BMC | 18 | 16811989 | 7.5 | 16811989 | 16811989 | 1 | 0 | 18-17039238 | Cdh2:12558:intron:2:105535:124008 |
| Tordoff3 | BMC | 19 | 21796849 | 7.6 | 21796849 | 21796849 | 1 | 0 | 19-20998286 | NA |
| Tordoff3 | BMD | 3 | 52959233 | 9.5 | 52816831 | 53105788 | 5 | 2 | 3-52955992 | NA |
| Tordoff3 | BMD | 5 | 106405797 | 10.1 | 106405797 | 106405797 | 1 | 0 | 5-103564644 | NA |
| Tordoff3 | BMD | 7 | 67373720 | 9.4 | 67322670 | 67565349 | 2 | 1 | 7-54531509 | NA |
| Tordoff3 | BMD | 13 | 9647467 | 10.1 | 7087999 | 11534269 | 20 | 10 | 13-9529316 | Dip2c:208440:intron:35:1180:7561 |
| Tordoff3 | bw_start | 1 | 154888397 | 9.0 | 153497361 | 154997648 | 2 | 3 | 1-153100355 | Lamc2:16782:intron:22:635:2530 |
| Tordoff3 | bw_start | 2 | 132434284 | 8.3 | 132434284 | 132453735 | 1 | 1 | 2-132491866 | 1110034G24Rik:73747:intron:4:50710:58079 |
| Tordoff3 | bw_start | 3 | 100368650 | 7.9 | 100368650 | 100368650 | 1 | 0 | 3-100177457 | NA |
| Tordoff3 | bw_start | 4 | 135935521 | 8.0 | 135935521 | 135935521 | 1 | 0 | 4-135121460 | Ephb2:13844:intron:9:8996:10925 |
| Tordoff3 | bw_start | 7 | 109237047 | 9.1 | 109237047 | 109237047 | 1 | 0 | 7-96716230 | NA |
| Tordoff3 | bw_start | 14 | 113284750 | 7.6 | 113284750 | 113448168 | 2 | 2 | rs6359032 | NA |
| Tordoff3 | CaCl2_pref25 | 3 | 119862138 | 9.0 | 119862138 | 119862138 | 1 | 0 | 3-120229856 | NA |
| Tordoff3 | CaCl2_pref25 | 3 | 122656153 | 8.7 | 122656153 | 123103540 | 2 | 0 | 3-123046195 | NA |
| Tordoff3 | CaCl2_pref25 | 6 | 30040008 | 7.9 | 30040008 | 30145614 | 1 | 1 | rs13478688 | Nrf1:18181:exon:2:73:221 |
| Tordoff3 | CaCl2_pref25 | 16 | 62393051 | 8.0 | 61793433 | 64377096 | 1 | 13 | rs4194384 | NA |
| Tordoff3 | CaCl2_pref25 | 17 | 49365605 | 8.5 | 49365605 | 51749291 | 2 | 16 | 17-48079168 | Kif6:319991:intron:19:4154:5367 |
| Tordoff3 | CaCl2_pref25 | 18 | 46025789 | 8.0 | 46025789 | 46181189 | 1 | 2 | rs13483339 | NA |
| Tordoff3 | CaCl2_pref7 | 3 | 39172191 | 8.1 | 38193516 | 39507448 | 2 | 1 | 3-38779929 | Fat4:329628:e20on:9:990:4348 |
| Tordoff3 | CaCl2_pref7 | 3 | 112373470 | 8.1 | 112191153 | 113118118 | 9 | 48 | 3-112635651 | NA |
| Tordoff3 | CaCl2_pref7 | 3 | 119862138 | 7.3 | 119862138 | 119862138 | 1 | 0 | 3-120229856 | NA |
| Tordoff3 | CaCl2_pref7 | 3 | 122656153 | 7.2 | 122656153 | 123103540 | 1 | 2 | 3-123046195 | NA |
| Tordoff3 | CaCl2_pref7 | 3 | 145793460 | 8.5 | 145793460 | 147180026 | 16 | 11 | rs13477472 | Ddah1:69219:intron:4:2796:35805 |
| Tordoff3 | CaCl2_pref7 | 3 | 150333553 | 7.0 | 150322196 | 150365369 | 1 | 6 | rs13477488 | NA |
| Tordoff3 | CaCl2_pref7 | 6 | 30040008 | 8.2 | 29017407 | 30271201 | 22 | 8 | rs13478688 | Nrf1:18181:exon:2:73:221 |
| Tordoff3 | CaCl2_pref7 | 6 | 73115071 | 7.0 | 73099680 | 73115071 | 1 | 1 | 6-73416985 | LOC668088:668088:intron:11:2766:6500 |
| Tordoff3 | CaCl2_pref7 | 7 | 29701931 | 7.1 | 29537077 | 29735630 | 1 | 14 | 7-18972845 | Zfp568:243905:intron:4:4062:8640 |
| Tordoff3 | CaCl2_pref7 | 7 | 56599478 | 7.2 | 56599478 | 56599478 | 1 | 0 | 7-44333938 | Gabrg3:14407:intron:4:3486:5157 |
| Tordoff3 | CaCl2_pref7 | 10 | 9060806 | 8.9 | 9060806 | 9060806 | 1 | 0 | 10-9047424 | NA |
| Tordoff3 | CaCl2_pref7 | 11 | 35382572 | 8.0 | 35188975 | 35774353 | 1 | 7 | 11-35220077 | Slit3:20564:intron:5:30854:36432 |
| Tordoff3 | CaCl2_pref7 | 11 | 39984533 | 7.4 | 39433021 | 40658895 | 3 | 12 | 11-39766949 | NA |
| Tordoff3 | CaCl2_pref7 | 12 | 65438564 | 7.6 | 64959362 | 65870860 | 3 | 9 | 12-59771675 | NA |
| Tordoff3 | CaCl2_pref7 | 14 | 27145151 | 6.9 | 27145151 | 27145151 | 1 | 0 | 14-24356832 | D14Ertd171e:238988:intron:15:15274:172591 |
| Tordoff3 | CaCl2_pref7 | 15 | 88247970 | 7.3 | 88247970 | 88248459 | 2 | 0 | 15-88805899 | NA |
| Tordoff3 | CaCl2_pref7 | 16 | 62393051 | 8.7 | 61745046 | 64000972 | 27 | 12 | rs4194384 | NA |
| Tordoff3 | CaCl2_pref7 | 17 | 49365605 | 8.7 | 48096628 | 52006333 | 3 | 22 | 17-48079168 | Kif6:319991:intron:19:4154:5367 |
| Tordoff3 | CaCl2_pref7 | 17 | 54746131 | 7.2 | 54620725 | 54753587 | 1 | 5 | rs3714226 | NA |
| Tordoff3 | CaCl2_pref7 | 17 | 75396698 | 7.8 | 75113561 | 75409518 | 3 | 2 | 17-73960857 | Rasgrp3:240168:intron:2:2688:2807 |
| Tordoff3 | CaCl2_pref7 | 17 | 91271204 | 7.3 | 91232488 | 91271204 | 2 | 0 | 17-89986651 | NA |
| Tordoff3 | CaCl2_pref7 | 19 | 60741196 | 7.6 | 60675713 | 60778512 | 3 | 2 | 19-60077524 | NA |
| Tordoff3 | CaCl2_pref7 | 20 | 81792762 | 7.2 | 81373306 | 82540796 | 3 | 8 | 20-77089605 | NA |
| Tordoff3 | CaCl2_pref75 | 17 | 17197676 | 8.1 | 17197676 | 17204067 | 1 | 1 | 17-16145181 | NA |
| Tordoff3 | CaLa_pref25 | 3 | 39172191 | 8.3 | 39172191 | 39305812 | 1 | 2 | 3-38779929 | Fat4:329628:e20on:9:990:4348 |
| Tordoff3 | CaLa_pref25 | 3 | 82522093 | 7.1 | 82521979 | 82522093 | 1 | 1 | rs13477228 | NA |
| Tordoff3 | CaLa_pref25 | 3 | 113027980 | 7.3 | 112191153 | 113118118 | 8 | 50 | 3-113288025 | NA |
| Tordoff3 | CaLa_pref25 | 3 | 119862138 | 8.3 | 119862138 | 119862138 | 1 | 0 | 3-120229856 | NA |
| Tordoff3 | CaLa_pref25 | 3 | 123103540 | 8.5 | 122656153 | 123103540 | 1 | 4 | 3-123504724 | Synpo2:118449:UTR:4:883:1437 |
| Tordoff3 | CaLa_pref25 | 3 | 145793460 | 7.6 | 145793460 | 147180026 | 5 | 15 | rs13477472 | Ddah1:69219:intron:4:2796:35805 |
| Tordoff3 | CaLa_pref25 | 3 | 150333553 | 7.0 | 150092496 | 150365369 | 1 | 7 | rs13477488 | NA |
| Tordoff3 | CaLa_pref25 | 4 | 95297052 | 8.0 | 95297052 | 95329389 | 1 | 3 | 4-94135309 | 2310009E04Rik:75578:intron:9:4368:11441 |
| Tordoff3 | CaLa_pref25 | 10 | 121764781 | 7.0 | 121625156 | 122416482 | 5 | 3 | 10-122101435 | NA |
| Tordoff3 | CaLa_pref25 | 12 | 64998700 | 7.3 | 64935624 | 65515662 | 6 | 12 | 12-59333472 | NA |
| Tordoff3 | CaLa_pref25 | 12 | 74796158 | 6.9 | 74728557 | 75244946 | 1 | 8 | 12-69157215 | NA |
| Tordoff3 | CaLa_pref25 | 16 | 62393051 | 7.9 | 61793433 | 64377096 | 11 | 21 | rs4194384 | NA |
| Tordoff3 | CaLa_pref25 | 16 | 70624468 | 7.8 | 70085830 | 72304810 | 5 | 15 | 16-71079970 | NA |
| Tordoff3 | CaLa_pref25 | 18 | 46025789 | 7.5 | 46003047 | 46181189 | 2 | 2 | rs13483339 | NA |
| Tordoff3 | CaLa_pref25 | 18 | 58466910 | 7.0 | 58466910 | 58466910 | 1 | 0 | 18-58856441 | NA |
| Tordoff3 | CaLa_pref7 | 3 | 39172191 | 9.2 | 39172191 | 40497484 | 2 | 4 | 3-38779929 | Fat4:329628:e20on:9:990:4348 |
| Tordoff3 | CaLa_pref7 | 3 | 113019815 | 8.2 | 112191153 | 113118118 | 34 | 23 | 3-113279815 | NA |
| Tordoff3 | CaLa_pref7 | 3 | 119862138 | 8.5 | 119862138 | 119862138 | 1 | 0 | 3-120229856 | NA |
| Tordoff3 | CaLa_pref7 | 3 | 123103540 | 9.1 | 122656153 | 123103540 | 3 | 2 | 3-123504724 | Synpo2:118449:UTR:4:883:1437 |
| Tordoff3 | CaLa_pref7 | 3 | 147146756 | 8.0 | 146741841 | 147180026 | 3 | 13 | 3-147784886 | NA |
| Tordoff3 | CaLa_pref7 | 4 | 95297052 | 8.3 | 95297052 | 95297052 | 1 | 0 | 4-94135309 | 2310009E04Rik:75578:intron:9:4368:11441 |
| Tordoff3 | CaLa_pref7 | 11 | 68925261 | 7.8 | 68925261 | 68925261 | 1 | 0 | rs3658906 | Per1:18626:intron:22:554:567 |
| Tordoff3 | CaLa_pref7 | 16 | 62328067 | 9.7 | 61745046 | 64377096 | 5 | 44 | 16-62648359 | NA |
| Tordoff3 | CaLa_pref7 | 16 | 70624468 | 7.7 | 70624468 | 70624468 | 1 | 0 | 16-71079970 | NA |
| Tordoff3 | CaLa_pref7 | 17 | 49422847 | 8.8 | 49365605 | 49729069 | 1 | 2 | 17-48136410 | NA |
| Tordoff3 | CaLa_pref7 | 17 | 54746131 | 8.3 | 54620725 | 54753587 | 1 | 5 | rs3714226 | NA |
| Tordoff3 | CaLa_pref7 | 17 | 70967083 | 7.6 | 70583580 | 71008667 | 1 | 6 | rs3717152 | Myom1:17929:intron:11:959:3179 |
| Tordoff3 | CaLa_pref7 | 17 | 75409518 | 7.7 | 75395170 | 75409518 | 1 | 2 | 17-73973680 | Rasgrp3:240168:intron:10:1068:6354 |
| Tordoff3 | CaLa_pref7 | 18 | 46025789 | 10.0 | 46003047 | 46200064 | 6 | 2 | rs13483339 | NA |
| Tordoff3 | CaLa_pref75 | 3 | 39172191 | 8.6 | 39172191 | 39182985 | 1 | 1 | 3-38779929 | Fat4:329628:e20on:9:990:4348 |
| Tordoff3 | CaLa_pref75 | 3 | 83296805 | 7.6 | 83296805 | 83324215 | 2 | 2 | 3-83606976 | NA |
| Tordoff3 | CaLa_pref75 | 3 | 119862138 | 9.8 | 119862138 | 119875430 | 1 | 1 | 3-120229856 | NA |
| Tordoff3 | CaLa_pref75 | 3 | 123103540 | 8.9 | 122656153 | 123103540 | 1 | 1 | 3-123504724 | Synpo2:118449:UTR:4:883:1437 |
| Tordoff3 | CaLa_pref75 | 3 | 150333553 | 8.0 | 150322196 | 150752711 | 1 | 5 | rs13477488 | NA |
| Tordoff3 | CaLa_pref75 | 4 | 43788869 | 8.1 | 43462088 | 44050716 | 1 | 6 | 4-43012862 | NA |
| Tordoff3 | CaLa_pref75 | 4 | 95329389 | 8.8 | 95297052 | 95329389 | 4 | 0 | 4-94167646 | NA |
| Tordoff3 | CaLa_pref75 | 4 | 98639563 | 8.4 | 98580006 | 98681640 | 1 | 3 | 4-97477820 | NA |
| Tordoff3 | CaLa_pref75 | 9 | 113763708 | 7.3 | 113592360 | 113763708 | 1 | 1 | rs3721068 | Clasp2:76499:intron:31:3393:3520 |
| Tordoff3 | CaLa_pref75 | 10 | 121764781 | 9.1 | 121621430 | 122199250 | 2 | 14 | 10-122101435 | NA |
| Tordoff3 | CaLa_pref75 | 12 | 64998700 | 8.1 | 64935624 | 65515662 | 9 | 4 | 12-59333472 | NA |
| Tordoff3 | CaLa_pref75 | 12 | 80325797 | 7.8 | 80325797 | 80325797 | 1 | 0 | 12-74705680 | Rad51l1:19363:intron:7:79551:330417 |
| Tordoff3 | CaLa_pref75 | 16 | 62393051 | 7.3 | 62393051 | 62829798 | 1 | 1 | rs4194384 | NA |
| Tordoff3 | CaLa_pref75 | 16 | 70624468 | 8.8 | 70085830 | 72299964 | 3 | 13 | 16-71079970 | NA |
| Tordoff3 | CaLa_pref75 | 18 | 12145966 | 7.8 | 11926038 | 12145966 | 1 | 3 | 18-12366660 | 6030446N20Rik:338363:intron:13:40783:49584 |
| Tordoff3 | fat_wt | 1 | 77470824 | 8.4 | 77470824 | 77598439 | 2 | 1 | 1-78041665 | NA |
| Tordoff3 | lean_wt | 1 | 153497361 | 8.2 | 153497361 | 153497361 | 1 | 0 | 1-151699108 | NA |
| Tordoff3 | lean_wt | 3 | 52959233 | 7.6 | 52816831 | 53020079 | 2 | 4 | 3-52955992 | NA |
| Tordoff3 | lean_wt | 6 | 129607112 | 7.1 | 129607112 | 129607112 | 1 | 0 | 6-130302836 | Klrc3:58179:e20on:3:152:175 |
| Tordoff3 | lean_wt | 9 | 36898753 | 7.4 | 36898753 | 36898753 | 1 | 0 | 9-37163643 | NA |
| Tordoff3 | lean_wt | 13 | 10048424 | 8.6 | 8305229 | 10839186 | 2 | 13 | 13-9933190 | Chrm3:12671:intron:3:73048:93726 |
| Tordoff3 | NaCl_pref225 | 1 | 147845343 | 7.4 | 147845343 | 147845343 | 1 | 0 | 1-145989496 | NA |
| Tordoff3 | NaCl_pref225 | 1 | 152903055 | 7.8 | 152903002 | 152911834 | 2 | 2 | 1-151083837 | NA |
| Tordoff3 | NaCl_pref225 | 2 | 79170267 | 8.6 | 79147916 | 81419825 | 9 | 10 | 2-79263487 | NA |
| Tordoff3 | NaCl_pref225 | 2 | 101620555 | 7.3 | 101620555 | 101620555 | 1 | 0 | 2-101713700 | NA |
| Tordoff3 | NaCl_pref225 | 4 | 27533558 | 7.7 | 27470533 | 27562783 | 1 | 5 | 4-27588741 | NA |
| Tordoff3 | NaCl_pref225 | 5 | 49088233 | 7.8 | 48553031 | 49167475 | 2 | 3 | 5-47428529 | NA |
| Tordoff3 | NaCl_pref225 | 6 | 71293068 | 8.1 | 71268782 | 71414925 | 1 | 3 | 6-71567727 | NA |
| Tordoff3 | NaCl_pref225 | 6 | 73627058 | 8.0 | 73522835 | 74139869 | 2 | 1 | rs3725176 | NA |
| Tordoff3 | NaCl_pref225 | 6 | 93615357 | 8.9 | 93615357 | 93885868 | 1 | 1 | rs13478899 | NA |
| Tordoff3 | NaCl_pref225 | 6 | 100771756 | 7.9 | 100771756 | 101237197 | 3 | 3 | 6-101195663 | NA |
| Tordoff3 | NaCl_pref225 | 6 | 109854605 | 9.4 | 109159365 | 109944006 | 16 | 8 | 6-110333693 | NA |
| Tordoff3 | NaCl_pref225 | 7 | 70534353 | 8.9 | 70534353 | 70534353 | 1 | 0 | 7-57698513 | NA |
| Tordoff3 | NaCl_pref225 | 8 | 52453032 | 8.7 | 52335908 | 54186231 | 8 | 19 | rs13479755 | NA |
| Tordoff3 | NaCl_pref225 | 9 | 22008421 | 7.5 | 22008421 | 22008887 | 4 | 0 | 9-22198800 | BC050092:235048:intron:2:24:4096 |
| Tordoff3 | NaCl_pref225 | 11 | 7418934 | 7.3 | 7418934 | 7418934 | 1 | 0 | 11-7413724 | NA |
| Tordoff3 | NaCl_pref225 | 11 | 66584027 | 8.6 | 66584027 | 66631044 | 1 | 1 | 11-66380380 | NA |
| Tordoff3 | NaCl_pref225 | 12 | 72169834 | 7.8 | 71986537 | 72169834 | 1 | 2 | 12-66520006 | NA |
| Tordoff3 | NaCl_pref225 | 13 | 31976505 | 7.7 | 31305427 | 34093571 | 5 | 19 | 13-31348722 | Gmds:218138:intron:7:131315:159752 |
| Tordoff3 | NaCl_pref225 | 13 | 111037136 | 7.3 | 110746416 | 111393176 | 1 | 7 | 13-106708398 | Pde4d:238871:intron:7:12568:30176 |
| Tordoff3 | NaCl_pref225 | 13 | 116295025 | 7.1 | 116295025 | 116295025 | 1 | 0 | 13-112125620 | NA |
| Tordoff3 | NaCl_pref225 | 14 | 35599216 | 8.0 | 35599216 | 35599216 | 1 | 0 | 14-32726544 | NA |
| Tordoff3 | NaCl_pref225 | 17 | 43548438 | 7.6 | 43548438 | 43682033 | 2 | 1 | 17-42235544 | Enpp5:83965:e20on:4:178:426 |
| Tordoff3 | NaCl_pref225 | 17 | 45630629 | 7.5 | 45630629 | 45911504 | 1 | 3 | 17-44323635 | Gtpbp2:56055:intron:9:396:571 |
| Tordoff3 | NaCl_pref225 | 17 | 92463158 | 7.6 | 92463158 | 92495144 | 1 | 1 | 17-91240251 | NA |
| Tordoff3 | NaCl_pref225 | 18 | 70043064 | 9.2 | 69245595 | 70403431 | 2 | 7 | rs13483425 | NA |
| Tordoff3 | NaCl_pref25 | 1 | 38542694 | 7.0 | 38542694 | 38615902 | 1 | 2 | 1-38918689 | Aff3:16764:intron:2:28939:88085 |
| Tordoff3 | NaCl_pref25 | 5 | 49167475 | 8.1 | 47183383 | 49738094 | 4 | 16 | 5-47507771 | NA |
| Tordoff3 | NaCl_pref25 | 14 | 71600590 | 7.4 | 71596271 | 71600590 | 1 | 1 | 14-64641474 | NA |
| Tordoff3 | NaCl_pref75 | 1 | 38542694 | 7.6 | 38542694 | 38542694 | 1 | 0 | 1-38918689 | Aff3:16764:intron:2:28939:88085 |
| Tordoff3 | NaCl_pref75 | 2 | 122001361 | 7.2 | 122001361 | 122052429 | 1 | 1 | 2-122058943 | BC019755:213696:intron:3:3010:4979 |
| Tordoff3 | NaCl_pref75 | 3 | 73855096 | 8.2 | 73653313 | 74132521 | 9 | 18 | 3-74189126 | NA |
| Tordoff3 | NaCl_pref75 | 5 | 47188785 | 7.9 | 47183383 | 49167475 | 8 | 12 | 5-45525697 | NA |
| Tordoff3 | NaCl_pref75 | 6 | 110741469 | 8.4 | 109159365 | 110759249 | 14 | 12 | 6-111224823 | Grm7:108073:intron:1:129304:267939 |
| Tordoff3 | NaCl_pref75 | 7 | 91169962 | 8.9 | 91169962 | 91169962 | 1 | 0 | 7-78527297 | Dlgh2:23859:intron:1:203720:358704 |
| Tordoff3 | NaCl_pref75 | 8 | 53849657 | 7.3 | 53841097 | 53849657 | 1 | 1 | 8-51518120 | NA |
| Tordoff3 | NaCl_pref75 | 8 | 66245359 | 7.8 | 65649352 | 66344690 | 3 | 3 | rs32894173 | Spock3:72902:intron:11:847:2892 |
| Tordoff3 | NaCl_pref75 | 9 | 75535008 | 7.2 | 75514387 | 75535008 | 1 | 1 | 9-76083583 | NA |
| Tordoff3 | NaCl_pref75 | 12 | 71986537 | 6.9 | 71986537 | 72451443 | 2 | 5 | 12-66334933 | Arid4a:238247:intron:16:593:2494 |
| Tordoff3 | NaCl_pref75 | 17 | 9894356 | 9.4 | 9837308 | 9894356 | 2 | 0 | 17-9466013 | NA |
| Tordoff3 | NaCl_pref75 | 18 | 70032539 | 7.3 | 69712454 | 70043064 | 1 | 3 | 18-70431947 | NA |
| Tordoff3 | NaLa_pref225 | 1 | 126518598 | 8.0 | 126518598 | 126714522 | 1 | 1 | 1-124502510 | NA |
| Tordoff3 | NaLa_pref225 | 2 | 66035737 | 7.2 | 66035526 | 66035963 | 1 | 3 | 2-66173534 | Ttc21b:73668:intron:11:143:3622 |
| Tordoff3 | NaLa_pref225 | 3 | 18917542 | 7.2 | 18917542 | 19083150 | 1 | 2 | rs13476999 | NA |
| Tordoff3 | NaLa_pref225 | 8 | 53390389 | 9.2 | 52335890 | 53849657 | 9 | 24 | 8-51060036 | NA |
| Tordoff3 | NaLa_pref225 | 10 | 28351460 | 7.4 | 28351460 | 28451138 | 1 | 1 | 10-28450661 | NA |
| Tordoff3 | NaLa_pref225 | 11 | 32950974 | 7.5 | 32950974 | 32950974 | 1 | 0 | 11-32946545 | NA |
| Tordoff3 | NaLa_pref225 | 19 | 50805817 | 7.3 | 50805817 | 50864114 | 1 | 2 | 19-50100452 | NA |
| Tordoff3 | NaLa_pref25 | 3 | 39305743 | 6.8 | 39247513 | 39521871 | 4 | 4 | 3-38913483 | NA |
| Tordoff3 | NaLa_pref25 | 6 | 109908830 | 6.9 | 109159365 | 109944006 | 1 | 18 | 6-110387918 | NA |
| Tordoff3 | NaLa_pref25 | 13 | 43254455 | 6.6 | 43158487 | 44118673 | 1 | 6 | rs3719648 | Gfod1:328232:intron:1:59761:101999 |
| Tordoff3 | NaLa_pref75 | 1 | 187484931 | 7.1 | 187484931 | 187484931 | 1 | 0 | 1-185422547 | NA |
| Tordoff3 | NaLa_pref75 | 3 | 18917542 | 7.2 | 18019992 | 19083150 | 4 | 6 | rs13476999 | NA |
| Tordoff3 | NaLa_pref75 | 3 | 39313571 | 7.6 | 39286009 | 40515484 | 4 | 8 | 3-38921311 | NA |
| Tordoff3 | NaLa_pref75 | 3 | 74132521 | 7.1 | 73855096 | 74132521 | 1 | 1 | 3-74466815 | NA |
| Tordoff3 | NaLa_pref75 | 4 | 24755301 | 7.3 | 24755301 | 24755301 | 1 | 0 | 4-24590581 | 6430524H05Rik:212390:intron:5:17382:31786 |
| Tordoff3 | NaLa_pref75 | 6 | 109159365 | 7.1 | 109159365 | 109854605 | 1 | 17 | 6-109637868 | NA |
| Tordoff3 | NaLa_pref75 | 8 | 51296393 | 8.2 | 51296393 | 54186231 | 10 | 39 | 8-48960400 | NA |
| Tordoff3 | NaLa_pref75 | 9 | 35265822 | 7.0 | 35265822 | 35265822 | 1 | 0 | 9-35486704 | NA |
| Tordoff3 | NaLa_pref75 | 13 | 3300355 | 7.0 | 3300355 | 3754054 | 1 | 1 | 13-3053402 | NA |
| Tordoff3 | NaLa_pref75 | 13 | 70633951 | 7.6 | 70633694 | 70637624 | 1 | 2 | 13-66606181 | NA |
| Tordoff3 | NaLa_pref75 | 18 | 70032539 | 8.4 | 69746427 | 70043064 | 2 | 2 | 18-70431947 | NA |
| Tordoff3 | NaLa_pref75 | 19 | 50805817 | 8.1 | 50805817 | 51373539 | 1 | 5 | 19-50100452 | NA |
| Tordoff3 | pct_fat | 1 | 34773259 | 7.6 | 34766566 | 34773259 | 1 | 2 | 1-35118389 | BC043098:214469:intron:3:52:8755 |
| Tordoff3 | pct_fat | 6 | 134947686 | 7.4 | 134853664 | 135165769 | 1 | 2 | 6-135033592 | NA |
| Tordoff3 | pct_fat | 8 | 58788222 | 8.4 | 58660356 | 60555271 | 8 | 4 | 8-54702286 | Adam29:244486:intron:1:10771:33310 |
| Tordoff3 | pct_fat | 8 | 84224181 | 7.6 | 84224181 | 84224181 | 1 | 0 | 8-80505135 | NA |
| Tordoff3 | pct_fat | 9 | 39981538 | 8.2 | 39981538 | 39981538 | 1 | 0 | 9-40256356 | NA |
| Tordoff3 | pct_fat | 9 | 87659598 | 7.4 | 87659598 | 87659598 | 1 | 0 | 9-88301925 | NA |
| Tordoff3 | pct_fat | 9 | 89769476 | 7.5 | 89327681 | 89769476 | 1 | 2 | 9-89798999 | Rasgrf1:19417:intron:8:495:3676 |
| Tordoff3 | pct_fat | 14 | 48536850 | 8.9 | 48496711 | 49504894 | 1 | 19 | 14-42291623 | NA |
| Tordoff3 | pct_fat | 15 | 84657099 | 7.4 | 84657099 | 84657099 | 1 | 0 | 15-85184941 | Phf21b:271305:intron:2:25405:46578 |
| Tordoff3 | pct_fat | 19 | 56322363 | 8.7 | 56139401 | 56364527 | 7 | 7 | 19-55652762 | NA |
| Tordoff3 | pct_lean | 1 | 34773259 | 7.4 | 34766566 | 34773259 | 1 | 2 | 1-35118389 | BC043098:214469:intron:3:52:8755 |
| Tordoff3 | pct_lean | 6 | 134947686 | 7.3 | 134831808 | 135165769 | 1 | 4 | 6-135033592 | NA |
| Tordoff3 | pct_lean | 8 | 58788222 | 8.2 | 58660356 | 60555271 | 8 | 4 | 8-54702286 | Adam29:244486:intron:1:10771:33310 |
| Tordoff3 | pct_lean | 8 | 84224181 | 7.5 | 84224181 | 84224181 | 1 | 0 | 8-80505135 | NA |
| Tordoff3 | pct_lean | 9 | 34706768 | 7.1 | 34682780 | 34706768 | 1 | 1 | 9-34936615 | Kirrel3:67703:intron:2:12576:46414 |
| Tordoff3 | pct_lean | 9 | 39981538 | 8.3 | 39981538 | 39981538 | 1 | 0 | 9-40256356 | NA |
| Tordoff3 | pct_lean | 9 | 48235042 | 6.9 | 48235042 | 48235042 | 1 | 0 | 9-48588921 | NA |
| Tordoff3 | pct_lean | 9 | 87659598 | 7.7 | 87477460 | 89769476 | 3 | 3 | 9-88301925 | NA |
| Tordoff3 | pct_lean | 14 | 48536850 | 8.9 | 48496711 | 49504894 | 4 | 17 | 14-42291623 | NA |
| Tordoff3 | pct_lean | 14 | 52736601 | 6.9 | 52736601 | 52736601 | 1 | 0 | 14-45756503 | NA |
| Tordoff3 | pct_lean | 15 | 84657099 | 7.3 | 84657099 | 84659216 | 1 | 1 | 15-85184941 | Phf21b:271305:intron:2:25405:46578 |
| Tordoff3 | pct_lean | 19 | 56322363 | 8.7 | 56139401 | 56410189 | 9 | 7 | 19-55652762 | NA |
| Tordoff3 | pct_lean | 20 | 66120325 | 6.9 | 66120325 | 66120325 | 1 | 0 | 20-61426007 | Aff2:14266:intron:15:131:4390 |
| Tordoff3 | pH | 1 | 98232027 | 7.7 | 98232027 | 98232027 | 1 | 0 | 1-96281262 | NA |
| Tordoff3 | pH | 18 | 75081888 | 9.0 | 75081888 | 75081888 | 1 | 0 | rs6302629 | Lipg:16891:intron:2:673:1597 |
| Tordoff3 | total_calcium | 1 | 174074829 | 9.4 | 173028365 | 174315872 | 20 | 63 | 1-172309466 | NA |
| Tordoff3 | total_calcium | 9 | 78912126 | 8.7 | 78893625 | 79768947 | 1 | 21 | 9-79526967 | NA |
| Tordoff3 | total_calcium | 15 | 27753212 | 7.9 | 27422444 | 27808343 | 1 | 4 | 15-27847351 | Trio:223435:intron:33:9439:12188 |
| Willott1 | ASR_100 | 1 | 4508893 | 6.5 | 4014866 | 4508893 | 1 | 1 | 1-4531465 | NA |
| Willott1 | ASR_100 | 1 | 179177709 | 6.9 | 179177709 | 179177709 | 1 | 0 | 1-177474164 | NA |
| Willott1 | ASR_100 | 1 | 181395604 | 7.2 | 181395604 | 181442629 | 2 | 0 | 1-179582173 | 9630058J23Rik:226744:intron:1:12312:32719 |
| Willott1 | ASR_100 | 2 | 7996254 | 7.2 | 7996254 | 8039711 | 2 | 2 | 2-8029506 | NA |
| Willott1 | ASR_100 | 2 | 179190927 | 7.1 | 179161451 | 179560373 | 1 | 4 | 2-178949726 | NA |
| Willott1 | ASR_100 | 3 | 149025859 | 6.2 | 149025859 | 149025859 | 1 | 0 | 3-149675133 | NA |
| Willott1 | ASR_100 | 4 | 147531281 | 6.4 | 147531281 | 147806872 | 1 | 1 | 4-146573423 | NA |
| Willott1 | ASR_100 | 5 | 73621851 | 6.4 | 72757428 | 73621851 | 1 | 6 | rs3708666 | NA |
| Willott1 | ASR_100 | 6 | 115519215 | 7.3 | 115251599 | 116211040 | 5 | 2 | 6-115943521 | Tsen2:381802:intron:3:3601:3995 |
| Willott1 | ASR_100 | 12 | 16389090 | 6.6 | 16275111 | 17074648 | 2 | 4 | 12-16493537 | NA |
| Willott1 | ASR_100 | 13 | 82585196 | 6.7 | 82585196 | 82585196 | 1 | 0 | 13-78728885 | NA |
| Willott1 | ASR_100 | 14 | 26715446 | 7.0 | 26715446 | 26715446 | 1 | 0 | 14-23929759 | D14Ertd171e:238988:intron:4:11899:21902 |
| Willott1 | ASR_100 | 14 | 45114218 | 7.0 | 44832603 | 45114218 | 1 | 1 | 14-38853582 | NA |
| Willott1 | ASR_100 | 14 | 73955530 | 6.4 | 72100859 | 74289982 | 3 | 6 | 14-66983506 | Lcp1:18826:intron:14:4912:7607 |
| Willott1 | ASR_100 | 14 | 122210799 | 7.1 | 122172937 | 123778489 | 10 | 15 | 14-115405073 | NA |
| Willott1 | ASR_100 | 15 | 14110410 | 6.3 | 13693622 | 14110410 | 1 | 1 | 15-14069163 | NA |
| Willott1 | ASR_100 | 15 | 17930730 | 6.5 | 17930730 | 17930730 | 1 | 0 | 15-18005612 | NA |
| Willott1 | ASR_100 | 16 | 29529580 | 7.3 | 29529580 | 29529580 | 1 | 0 | 16-29461640 | Opa1:74143:intron:9:1366:2200 |
| Willott1 | ASR_100 | 18 | 59885647 | 7.3 | 59885647 | 61174703 | 2 | 0 | 18-60275868 | NA |
| Willott1 | ASR_100 | 19 | 23933846 | 7.2 | 23933846 | 24419557 | 1 | 1 | 19-23139421 | Apba1:319924:intron:1:107657:133642 |
| Willott1 | ASR_100 | 20 | 138523152 | 6.8 | 138523152 | 138523152 | 1 | 0 | 20-133167907 | NA |
| Willott1 | ASR_70 | 1 | 73923494 | 6.9 | 72189171 | 75756999 | 3 | 8 | 1-74433463 | Tns1:21961:intron:12:23:3933 |
| Willott1 | ASR_70 | 1 | 140663039 | 7.7 | 140663039 | 140663039 | 1 | 0 | 1-138730345 | Lh209:16876:intron:1:546:4729 |
| Willott1 | ASR_70 | 7 | 30029980 | 7.3 | 29834410 | 30092266 | 2 | 6 | 7-19298291 | NA |
| Willott1 | ASR_70 | 8 | 78108390 | 6.3 | 78108390 | 78108390 | 1 | 0 | 8-74321197 | NA |
| Willott1 | ASR_70 | 9 | 84914257 | 6.1 | 84914257 | 84914257 | 1 | 0 | 9-85546504 | NA |
| Willott1 | ASR_70 | 10 | 124947366 | 6.2 | 124364549 | 124947366 | 1 | 4 | 10-125330369 | NA |
| Willott1 | ASR_70 | 11 | 40694517 | 6.4 | 39627152 | 40694612 | 1 | 13 | 11-40476933 | NA |
| Willott1 | ASR_70 | 12 | 35003462 | 6.1 | 34983589 | 35003462 | 1 | 1 | 12-29439828 | Hdac9:79221:intron:6:11281:21598 |
| Willott1 | ASR_70 | 13 | 31599567 | 6.6 | 31176015 | 34104408 | 8 | 11 | 13-30971784 | NA |
| Willott1 | ASR_70 | 13 | 91132731 | 7.2 | 91132478 | 91610129 | 2 | 2 | 13-87331201 | NA |
| Willott1 | ASR_70 | 16 | 64505613 | 6.7 | 62952275 | 64555834 | 2 | 18 | 16-64855966 | NA |
| Willott1 | ASR_70 | 16 | 75768208 | 6.2 | 75701538 | 75790048 | 1 | 4 | 16-76242881 | Samsn1:67742:intron:2:3211:4711 |
| Willott1 | ASR_70 | 19 | 27543922 | 6.8 | 27493125 | 28562707 | 4 | 6 | 19-26753246 | NA |
| Willott1 | ASR_70 | 20 | 117184931 | 6.6 | 117184931 | 117308718 | 1 | 2 | 20-112399014 | NA |
| Willott1 | ASR_80 | 1 | 9013536 | 7.3 | 8932764 | 9014287 | 2 | 6 | rs3714728 | Sntg1:71096:intron:2:179575:203928 |
| Willott1 | ASR_80 | 3 | 65563597 | 8.1 | 65479298 | 65564847 | 3 | 2 | 3-65713814 | NA |
| Willott1 | ASR_80 | 6 | 115508181 | 7.4 | 115508181 | 115519475 | 1 | 1 | 6-115932106 | NA |
| Willott1 | ASR_80 | 14 | 73955530 | 8.9 | 73653536 | 74386266 | 3 | 5 | 14-66983506 | Lcp1:18826:intron:14:4912:7607 |
| Willott1 | ASR_80 | 15 | 14581983 | 7.3 | 14291562 | 14581983 | 3 | 2 | 15-14542151 | NA |
| Willott1 | ASR_90 | 1 | 4508893 | 7.0 | 4014866 | 5281856 | 3 | 0 | 1-4531465 | NA |
| Willott1 | ASR_90 | 1 | 181395604 | 8.2 | 181395604 | 181608911 | 2 | 1 | 1-179582173 | 9630058J23Rik:226744:intron:1:12312:32719 |
| Willott1 | ASR_90 | 2 | 7996254 | 6.9 | 7996254 | 8815700 | 2 | 6 | 2-8029506 | NA |
| Willott1 | ASR_90 | 2 | 161431812 | 6.1 | 161431812 | 161737100 | 1 | 1 | rs6204920 | Ptprt:19281:intron:12:25852:33307 |
| Willott1 | ASR_90 | 2 | 179190927 | 6.5 | 179190927 | 179560373 | 1 | 1 | 2-178949726 | NA |
| Willott1 | ASR_90 | 3 | 149025859 | 6.5 | 149025859 | 149025859 | 1 | 0 | 3-149675133 | NA |
| Willott1 | ASR_90 | 5 | 73621851 | 7.9 | 73117963 | 73713197 | 2 | 8 | rs3708666 | NA |
| Willott1 | ASR_90 | 6 | 115508181 | 7.0 | 115503567 | 116211040 | 5 | 7 | 6-115932106 | NA |
| Willott1 | ASR_90 | 12 | 16389090 | 6.4 | 16275111 | 16607820 | 1 | 4 | 12-16493537 | NA |
| Willott1 | ASR_90 | 14 | 26715446 | 6.6 | 26715446 | 26715446 | 1 | 0 | 14-23929759 | D14Ertd171e:238988:intron:4:11899:21902 |
| Willott1 | ASR_90 | 14 | 45114218 | 6.4 | 45114218 | 45114218 | 1 | 0 | 14-38853582 | NA |
| Willott1 | ASR_90 | 14 | 72132035 | 6.9 | 71740249 | 74289982 | 7 | 6 | 14-65168960 | NA |
| Willott1 | ASR_90 | 14 | 122456924 | 6.3 | 122172937 | 122787050 | 6 | 7 | 14-115654691 | Vgcnl1:338370:intron:19:1555:1747 |
| Willott1 | ASR_90 | 15 | 14110410 | 6.2 | 13693622 | 14581983 | 1 | 6 | 15-14069163 | NA |
| Willott1 | ASR_90 | 16 | 38504721 | 6.4 | 37998387 | 38504721 | 1 | 2 | rs4174648 | Tmem39a:67846:exon:6:212:341 |
| Willott1 | ASR_90 | 18 | 59885647 | 7.7 | 59885647 | 61174703 | 2 | 0 | 18-60275868 | NA |
| Willott1 | ASR_90 | 19 | 23933846 | 6.2 | 23933846 | 24419557 | 1 | 1 | 19-23139421 | Apba1:319924:intron:1:107657:133642 |
| Willott1 | ASR_90 | 20 | 94479835 | 6.2 | 94126733 | 94479835 | 1 | 1 | rs3672400 | Ar:11835:intron:2:25277:34749 |
| Willott1 | ASR_90 | 20 | 138523152 | 6.5 | 138523152 | 138601136 | 1 | 2 | 20-133167907 | NA |
| Willott1 | ASR_habituation | 10 | 89703068 | 5.8 | 88783017 | 89703068 | 1 | 3 | 10-90037262 | Anks1b:77531:intron:10:12450:46748 |
| Willott1 | ASR_habituation | 16 | 30420370 | 6.8 | 30318785 | 30438348 | 3 | 1 | 16-30364993 | NA |
| Willott1 | ASR_latency | 4 | 92039712 | 6.6 | 91969038 | 92039712 | 1 | 1 | 4-90877969 | NA |
| Willott1 | ASR_latency | 5 | 73394098 | 6.9 | 73149945 | 73915057 | 2 | 15 | 5-71754151 | 9030227G01Rik:320336:intron:11:3520:5629 |
| Willott1 | ASR_latency | 8 | 117729911 | 6.5 | 117720557 | 117729911 | 1 | 3 | 8-114214218 | Wwo20:80707:intron:8:131682:639384 |
| Willott1 | ASR_latency | 13 | 54988098 | 6.6 | 54988098 | 54988098 | 1 | 0 | 13-54068109 | Unc5a:107448:intron:1:28743:41146 |
| Willott1 | bw | 1 | 109410577 | 7.2 | 108589634 | 109411688 | 1 | 8 | 1-107588251 | NA |
| Willott1 | bw | 5 | 91884740 | 6.4 | 91884740 | 91935904 | 1 | 2 | 5-89620357 | NA |
| Willott1 | bw | 7 | 68289848 | 6.6 | 68289848 | 68289848 | 1 | 0 | 7-55449189 | NA |
| Willott1 | bw | 14 | 75459644 | 6.4 | 75459644 | 75514248 | 1 | 1 | 14-68487280 | NA |
| Willott1 | bw | 15 | 82862314 | 7.9 | 82046556 | 82862314 | 1 | 5 | 15-83391809 | NA |
| Willott1 | bw | 16 | 75701538 | 6.7 | 75674118 | 75768208 | 2 | 4 | 16-76175895 | NA |
| Willott1 | PPI_12 | 1 | 81967960 | 7.9 | 81425338 | 82024106 | 4 | 10 | 1-82607541 | NA |
| Willott1 | PPI_12 | 2 | 179190927 | 7.1 | 178727616 | 179560373 | 1 | 6 | 2-178949726 | NA |
| Willott1 | PPI_12 | 5 | 107067585 | 6.9 | 107067585 | 107067585 | 1 | 0 | 5-104260866 | NA |
| Willott1 | PPI_12 | 8 | 72759434 | 6.2 | 72005293 | 73973097 | 1 | 19 | 8-68863655 | Edg4:53978:UTR:4:2517:3174 |
| Willott1 | PPI_12 | 12 | 16389090 | 6.8 | 16274798 | 16573197 | 1 | 4 | 12-16493537 | NA |
| Willott1 | PPI_12 | 12 | 90592192 | 6.7 | 90574980 | 90629217 | 1 | 7 | 12-84778433 | Nr20n3:18191:intron:16:18074:30054 |
| Willott1 | PPI_20 | 1 | 73672586 | 6.8 | 73672586 | 73884001 | 1 | 1 | 1-74178183 | NA |
| Willott1 | PPI_20 | 1 | 79795005 | 8.2 | 79795005 | 81967999 | 7 | 5 | 1-80429528 | LOC433326:433326:intron:2:6292:14319 |
| Willott1 | PPI_20 | 5 | 107067585 | 8.1 | 107067585 | 107223509 | 1 | 3 | 5-104260866 | NA |
| Willott1 | PPI_20 | 12 | 90579085 | 7.3 | 90579085 | 90625963 | 1 | 4 | 12-84765326 | Nr20n3:18191:intron:16:4967:30054 |
| Willott1 | PPI_20 | 15 | 103204481 | 7.0 | 103204481 | 103204481 | 1 | 0 | 15-103860972 | NA |
| Willott1 | PPI_4 | 2 | 129350400 | 5.9 | 129197313 | 130232802 | 3 | 24 | 2-129407982 | NA |
| Willott1 | PPI_4 | 6 | 78412502 | 6.1 | 78306976 | 78412715 | 1 | 2 | 6-78720850 | NA |
| Willott1 | PPI_4 | 6 | 80799010 | 6.8 | 80795891 | 80799010 | 2 | 0 | 6-81093621 | NA |
| Willott1 | PPI_4 | 7 | 16903755 | 6.8 | 16359361 | 17067974 | 2 | 6 | 7-9392343 | Psg30:73250:intron:2:30098:30248 |
| Willott1 | PPI_4 | 7 | 122413490 | 6.1 | 122413490 | 122413490 | 1 | 0 | 7-109920872 | Prkcb1:18751:intron:13:21510:26823 |
| Willott1 | PPI_4 | 8 | 87319021 | 6.6 | 86220194 | 87344761 | 4 | 1 | 8-83699203 | Cacna1a:12286:intron:1:12972:45544 |
| Willott1 | PPI_4 | 9 | 7639429 | 6.2 | 7639429 | 7639429 | 1 | 0 | 9-7615890 | Mmp20:30800:intron:3:67:3333 |
| Willott1 | PPI_4 | 11 | 22078750 | 6.7 | 21864258 | 22114857 | 1 | 4 | 11-22074317 | Ehbp1:216565:intron:4:53253:58993 |
| Willott1 | PPI_tot | 1 | 77146929 | 6.5 | 77146929 | 77146929 | 1 | 0 | 1-77720622 | NA |
| Willott1 | PPI_tot | 1 | 79225628 | 6.5 | 79225628 | 79795005 | 2 | 2 | rs3698264 | NA |
| Willott1 | PPI_tot | 1 | 81967960 | 8.2 | 81425338 | 81967999 | 4 | 3 | 1-82607541 | NA |
| Willott1 | PPI_tot | 5 | 107067585 | 6.7 | 107067585 | 107067585 | 1 | 0 | 5-104260866 | NA |
| Willott1 | PPI_tot | 8 | 72759434 | 6.5 | 72005119 | 75517572 | 1 | 60 | 8-68863655 | Edg4:53978:UTR:4:2517:3174 |
| Willott1 | PPI_tot | 8 | 95187863 | 6.2 | 95187863 | 95187863 | 1 | 0 | 8-91646693 | NA |
| Willott1 | PPI_tot | 11 | 21944967 | 6.5 | 21864258 | 22114857 | 1 | 3 | 11-21940534 | Ehbp1:216565:intron:20:8466:38475 |
| Willott1 | PPI_tot | 12 | 90586900 | 6.7 | 90579085 | 90625963 | 2 | 3 | 12-84773141 | Nr20n3:18191:intron:16:12783:30054 |
| Willott1 | PPI_tot | 13 | 47358818 | 6.7 | 47358818 | 47686862 | 1 | 4 | rs3670228 | NA |
| Willott1 | PPI_tot | 19 | 14259794 | 6.6 | 14259652 | 14388349 | 2 | 2 | 19-13469751 | NA |

NA: non-genic regions
