# Supplementary figures and images for: Large-Scale In Silico Mapping of Complex Quantitative Traits in Inbred Mice
Source: PLoS One. 2007 Jul 25;2(7):e651. doi: 10.1371/journal.pone.0000651 (PMC1920557; doi:10.1371/journal.pone.0000651)

**A**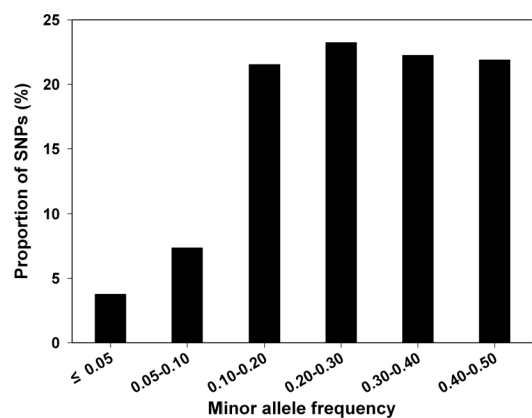**B**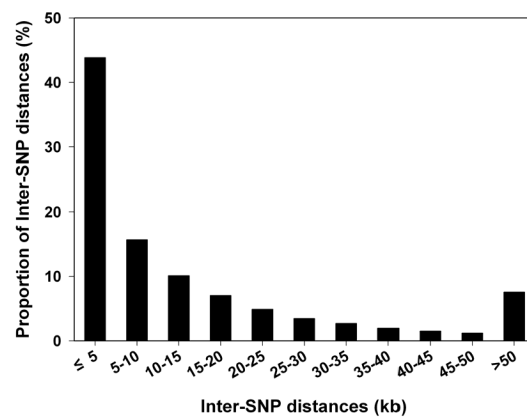**C**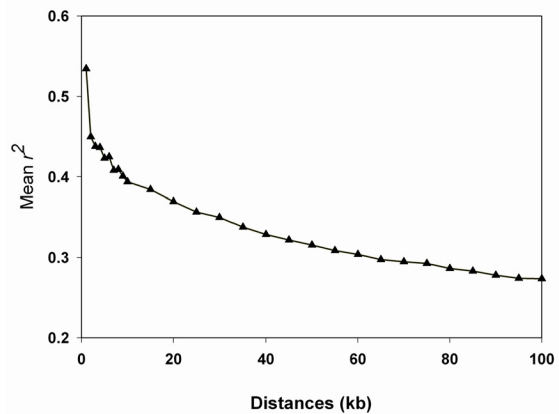**D**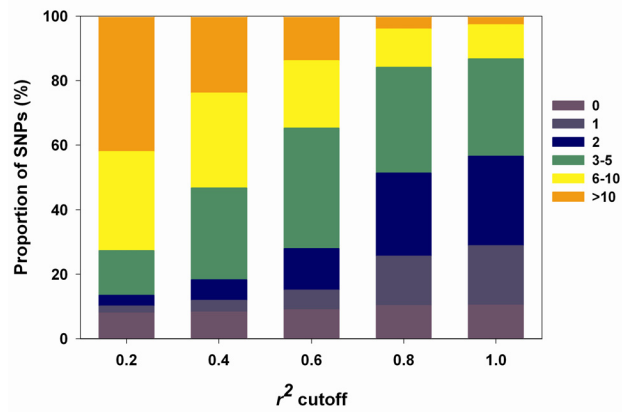

Supplement: Figure S2 — Characteristics of the mouse SNP map. (A) Distribution of SNP allele frequency. (B) Distribution of inter-SNP distances. (C) LD decay as a function of physical distance. (D) Number of proxies per SNP in a window size of 500 kb, as a function of the threshold for correlation (rˆ2). (0.90 MB PDF) [file pone.0000651.s005.pdf]

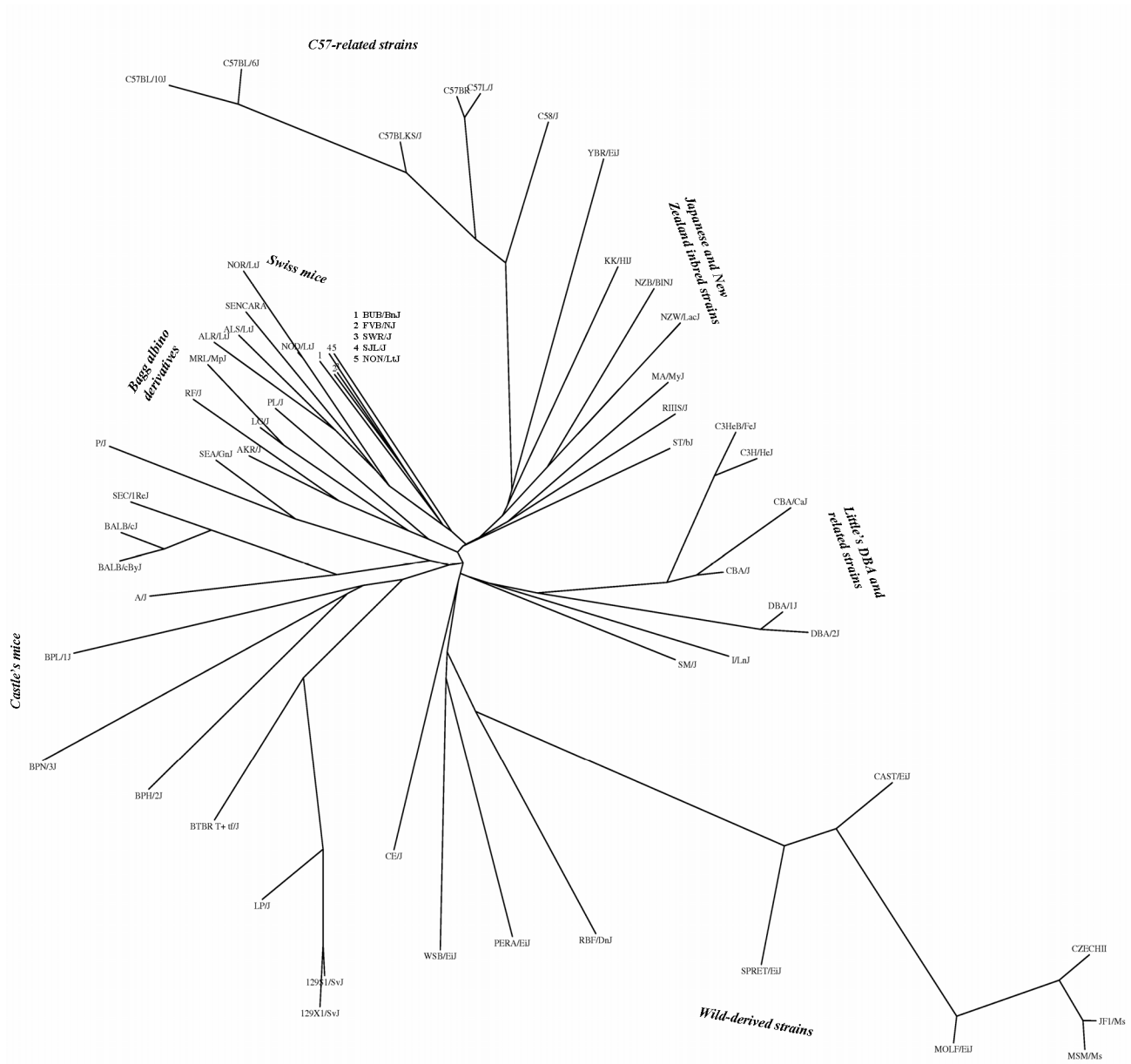

Supplement: Figure S3 — Phylogenetic tree of 59 inbred mouse strains. The phylogenetic tree was constructed with a total of 148,062 SNPs from the WTCHG and Broad Institute, implemented in the dnadist program in the PHYLIP 3.66 package (http://evolution.genetics.washington.edu/phylip.html). The branch length information was used to plot evolutionary distance between strains in the drawtree program. 59 inbred mouse strains are organized into seven groups: Bagg albino derivatives, C57-related strains, Castle's mice, Japanese and New Zealand inbred strains, Little's DBA and related strains, Swiss mice, and wild-derived strains. (1.41 MB PDF) [file pone.0000651.s006.pdf]

**A**

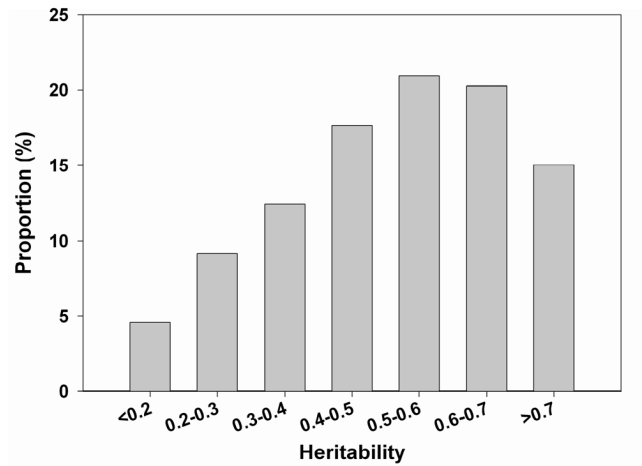

**B**

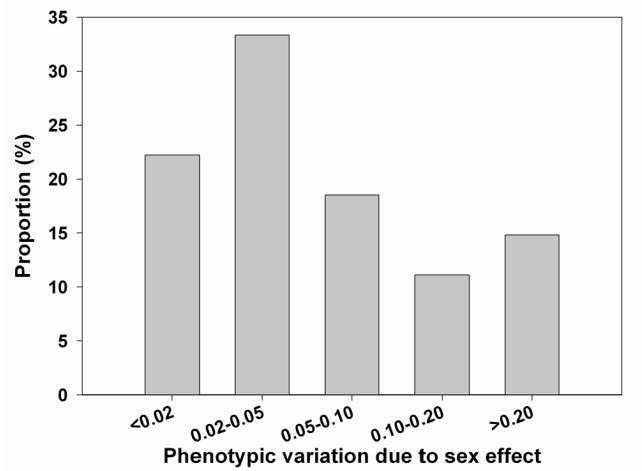

Supplement: Figure S4 — Characteristics of 173 complex quantitative traits in inbred mice. (A) Heritability of quantitative traits. (B) Phenotypic variation due to sex effects. (0.70 MB PDF) [file pone.0000651.s007.pdf]

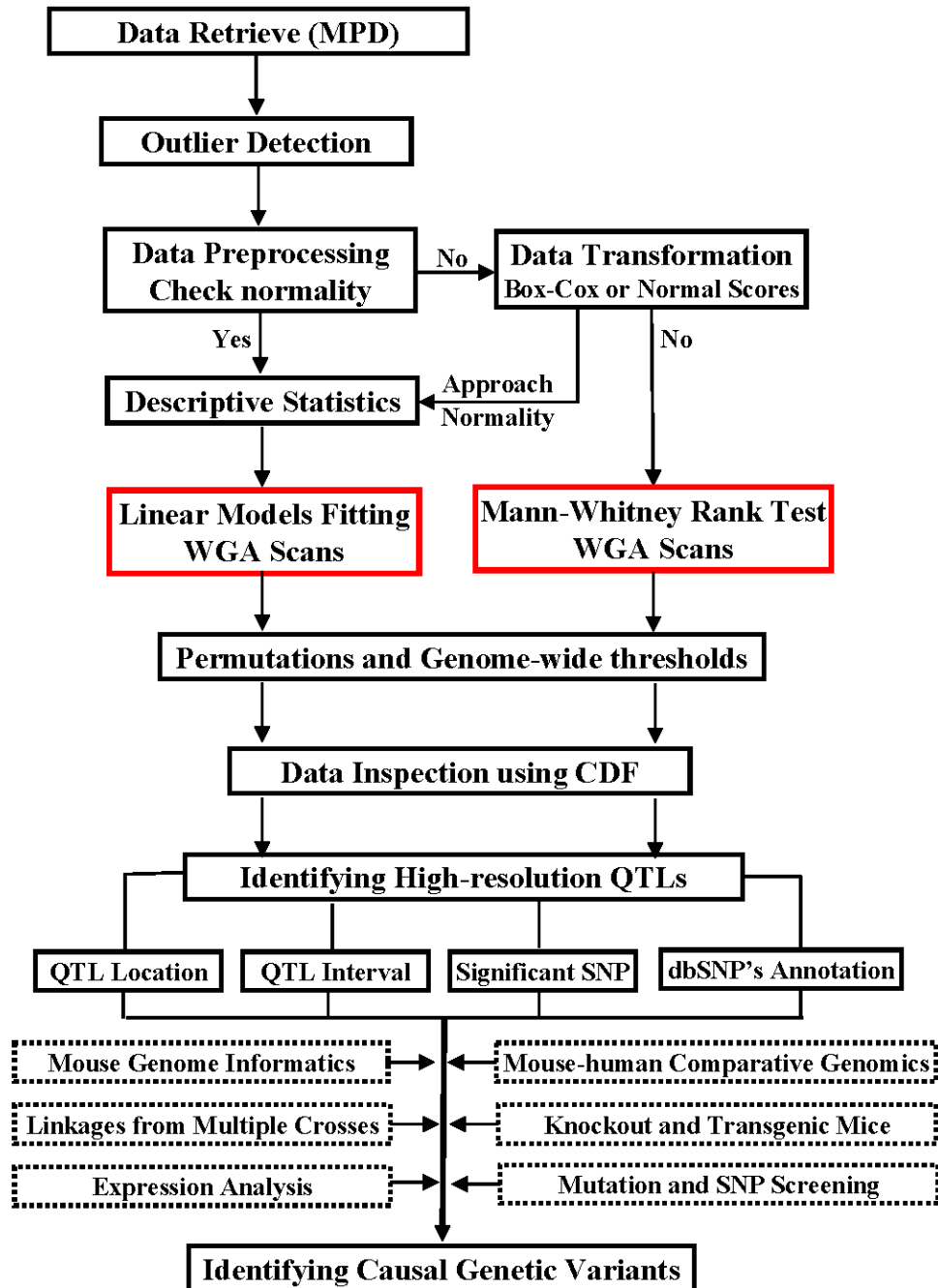

Supplement: Figure S5 — An in silico strategy for high-throughput gene discovery in inbred mice. GWA scans were implemented in an automatic processing pipeline which constitutes data retrieving, outlier detection, data preprocessing, hypothesis testing, permutations and QTL identification. (0.21 MB PDF) [file pone.0000651.s008.pdf]

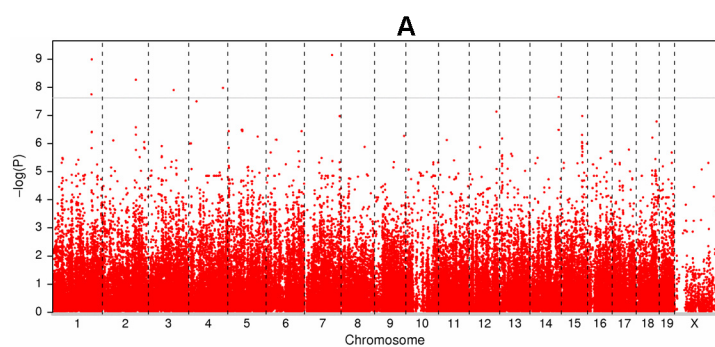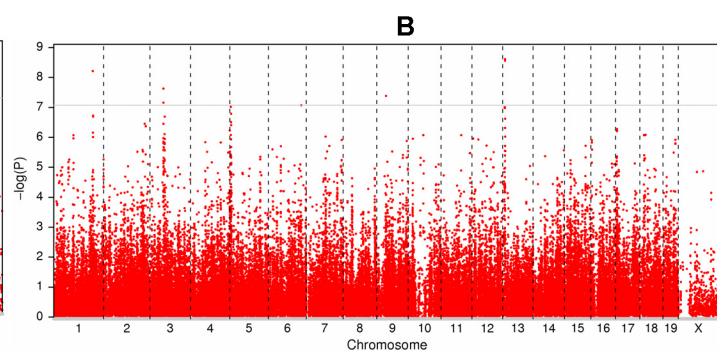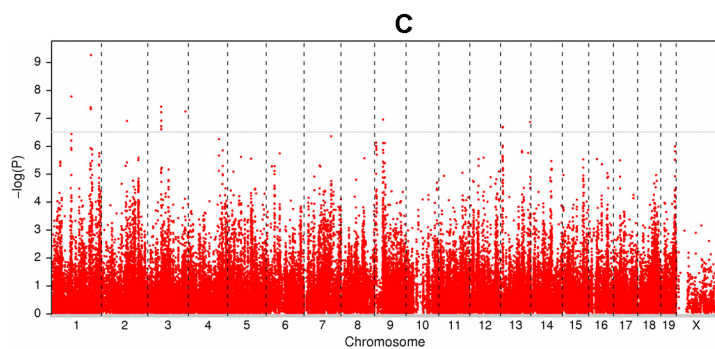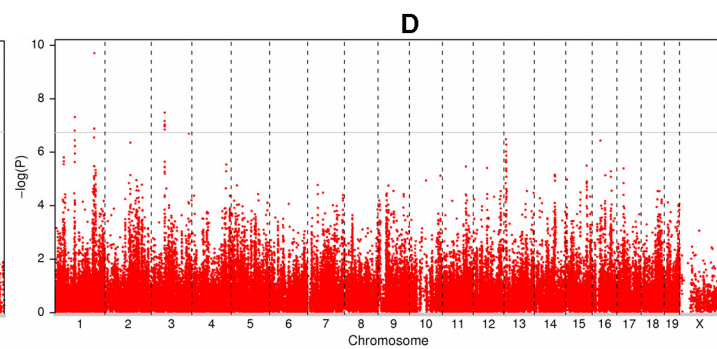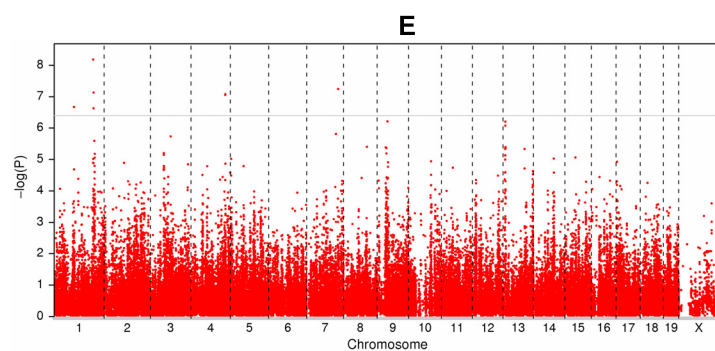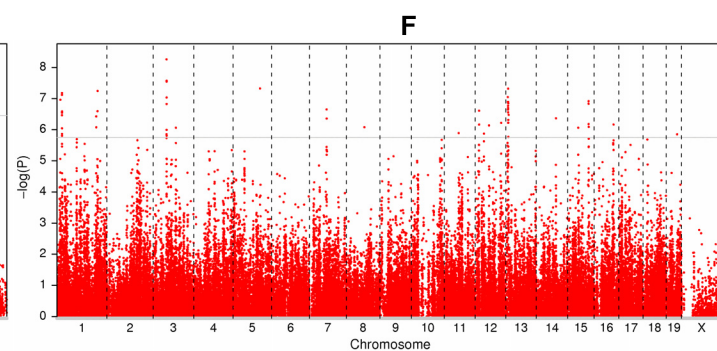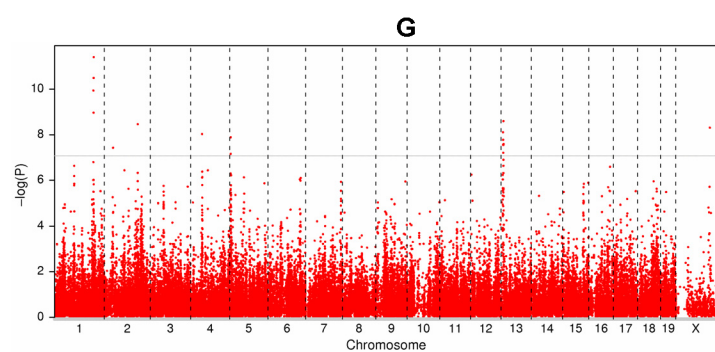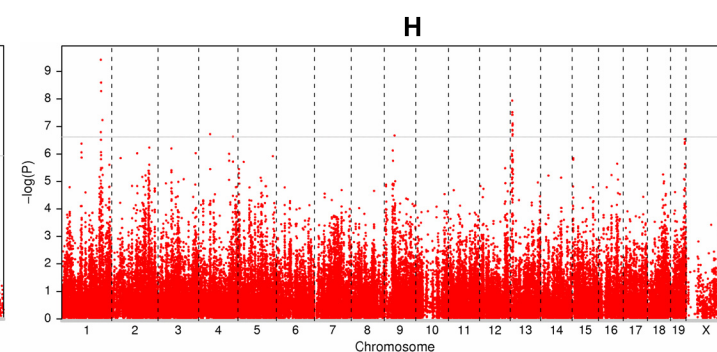

Supplement: Figure S6 — Genome-wide association analysis of several obesity-related phenotypes in inbred mice. The obesity-relaed phenotypes are (A) body weight at the start of testing (8 weeks) (Tordoff3_bw_start); (B) calculated weight of lean tissue (14 weeks) (Tordoff3_lean_wt); (C) body weight after 8 weeks on an atherogenic diet (Naggert1_bw_fat8); (D) total tissue mass after 8 weeks on an atherogenic diet (Naggert1_tissuemass_fat8); (E) weight of lean portion of tissue mass after 8 weeks on an atherogenic diet (Naggert1_leanwt_fat8); (F) bone mineral content after 8 weeks on an atherogenic diet (Naggert1_BMC_fat8); (G) initial body weight (7–9 weeks), day 0 of an atherogenic diet (Paigen1_initbw); and (H) final body weight after 8 weeks on an atherogenic diet (Paigen1_finalbw). In Paigen1 and Naggert1 projects, mice at 7–9 weeks of age were weighed and then administered a high fat, high cholesterol atherogenic diet. The scatter plots were drawn for -log (P) against the SNP position in the chromosomes. The horizontal gray lines indicate genome-wide empirical thresholds (global p value = 0.05). The horizontal coordinates were plotted using physical distance (Mb) in each panel. (7.96 MB PDF) [file pone.0000651.s009.pdf]

**A**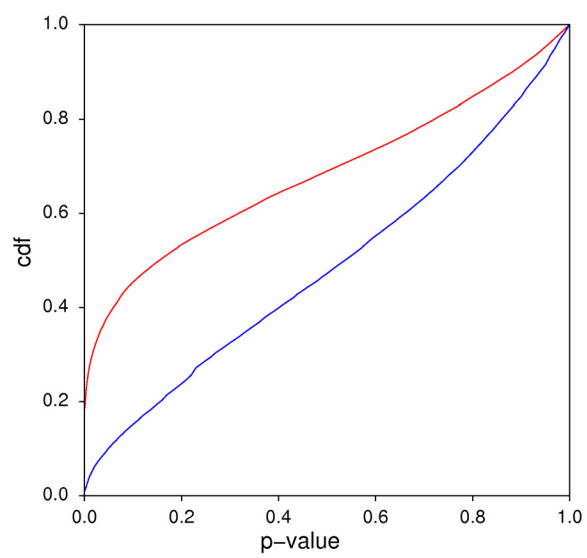**B**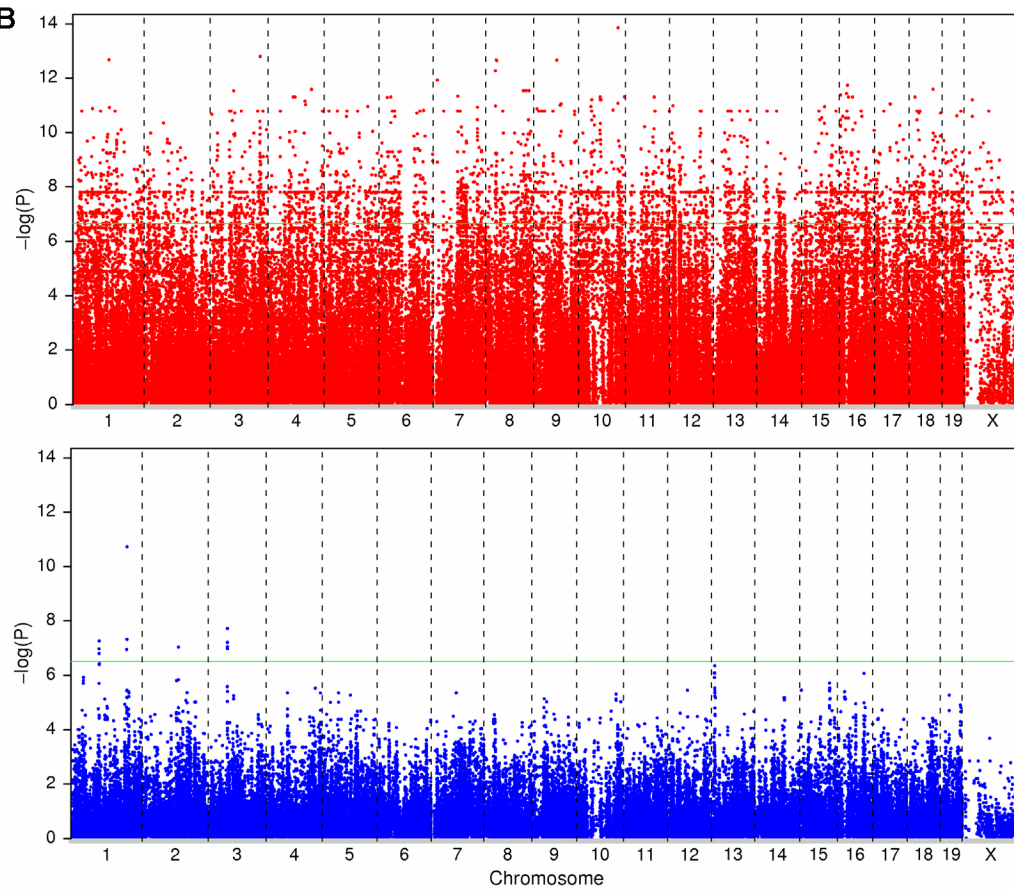

Supplement: Figure S7 — Potential population structure in inbred mice. Body weight after 8 weeks on altherogenic diet (Naggert1_bw_fat8) was used to illustrate the population structure in inbred mice. (A) Cummlative distribution of p values from GWA analysis of samples with (red line) and without (blue line) wild-derived inbred strains. cdf, cumulative distribution function. (B) Comparison of GWA analysis of samples with (upper, red) and without (lower, blue) wild-derived inbred strains. The two green horizontal lines are genome-wide thresholds (a global p value of 0.05). Spurious assocaitons were largely reduced in the analysis after the removal of wild-derived inbred mouse strains. (1.78 MB PDF) [file pone.0000651.s010.pdf]

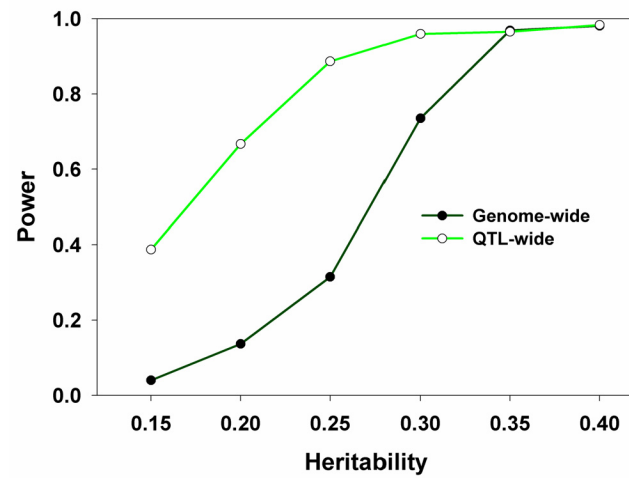

Supplement: Figure S8 — Power of association analysis in inbred mice. Power was estimated for different trait heritabilities under genome-wide and QTL-wide thresholds (P = 0.05). The genome-wide threshold is used to declare a significant association on the genome without any prior genetic evidence; while the QTL-wide threshold is used to declare a significant association on a privious linkage-defined region. (0.48 MB PDF) [file pone.0000651.s011.pdf]
