# Supplementary material for: Large-Scale In Silico Mapping of Complex Quantitative Traits in Inbred Mice
Source: PLoS One. 2007 Jul 25;2(7):e651. doi: 10.1371/journal.pone.0000651 (PMC1920557; doi:10.1371/journal.pone.0000651)
Supplement: Figure S1 — Distribution of SNP positions and LD structure across the mouse genome. For each chromosome, the top panel shows number of SNPs in a window size of 500 kb. The bottom panel shows number of proxies (red lines for rˆ2>0.5 and blue lines for rˆ2>0.8) per SNP in a window size of 500 kb. (0.41 MB PDF) [file pone.0000651.s004.pdf]

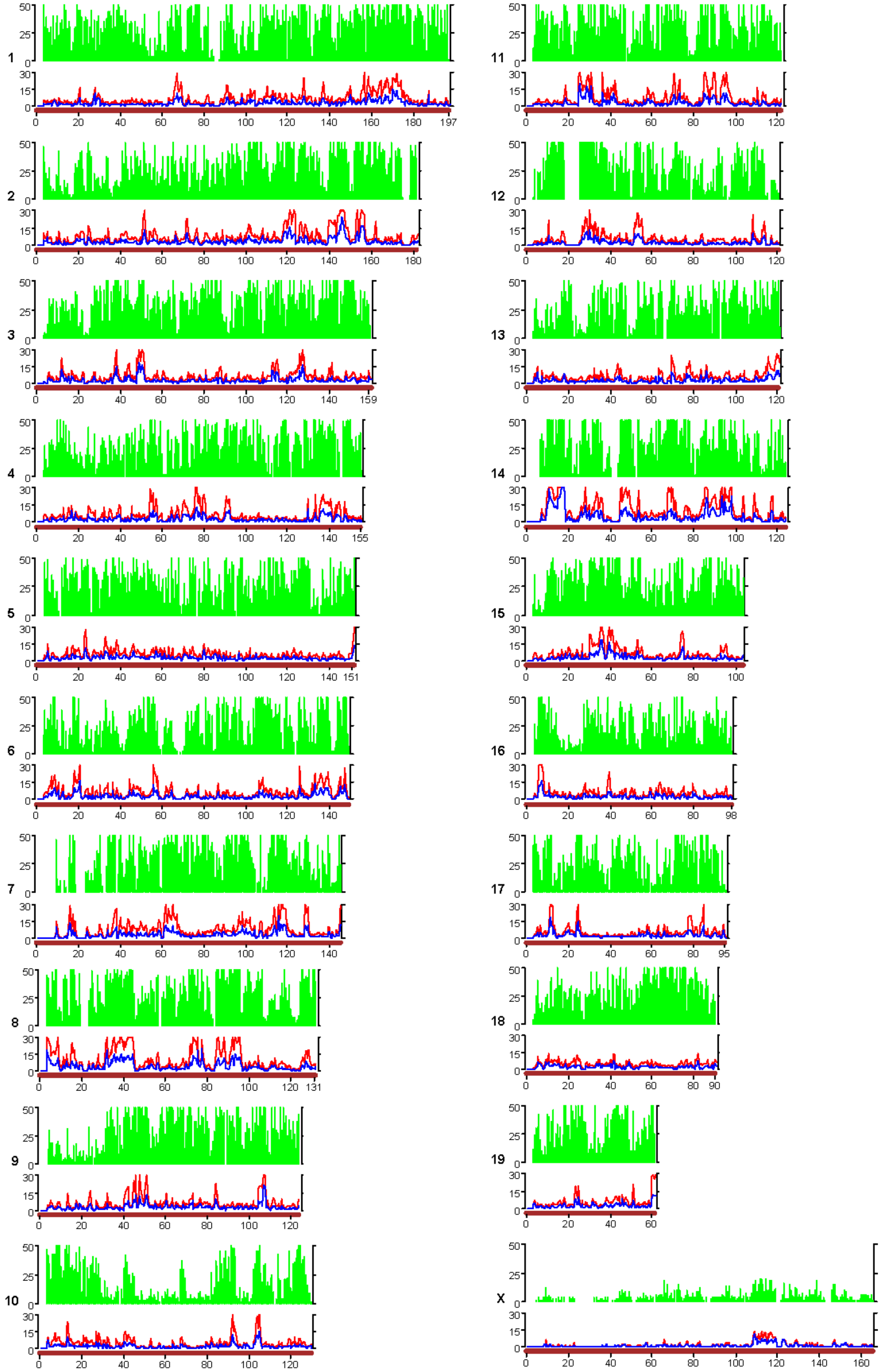
1
2
3
4
5
6
7
8
9
10
11
12
13
14
15
16
17
18
19
X
